# Supplementary material for: Ecological niche divergence or ecological niche partitioning in a widespread Neotropical bird lineage
Source: PeerJ. 2024 Apr 30;12:e17345. doi: 10.7717/peerj.17345 (PMC11067898; doi:10.7717/peerj.17345)
Supplement: Supplemental Information 1 — Additional figures related to niche differentiation tests and to niche evolution analyses. [file peerj-12-17345-s001.pdf]

Appendix 1

Jacob C. Cooper

2024-02-27

Concatenated here are all additional figures. Please note that some figures are missing, as the code is written to 'skip' figures that are identity plots or repeated plots. All unique and relevant figures are shown.

```
set.seed(2024)

library(tidyverse)
library(data.table)
library(rasterVis)
library(lattice)
```

Visualizing differences

```
files=list.files(filepath,pattern="*mve_comparisons.csv")

sp.comp.files=files[files%like%"Progne"]
subis.comp.files=files[-which(files%like%"Progne")]
```

A: Species comparisons

```
for(i in 1:length(sp.comp.files)){
  sp=strsplit(sp.comp.files[i],"_")[[1]][2]
  x1=read_csv(paste0(filepath,sp.comp.files[i]))
  for(ii in 1:ncol(x1)){
    comp.sp=strsplit(colnames(x1)[ii],"_")[[1]][2]
    list.1=x1[,ii]

    if(comp.sp==sp){next}
    x2=read_csv(paste0(filepath,sp.comp.files[sp.comp.files%like%comp.sp]))
    list.2=x2[-1,which(colnames(x2)%like%sp)]

    list.1$ID=paste0(sp,'_',comp.sp)
    list.2$ID=paste0(comp.sp,'_',sp)

    test_val=list.1[1,1]
    list.1=list.1[-1,]

    colnames(list.1)=colnames(list.2)=c("Value","ID")

    val.list=as.data.frame(rbind(list.1,list.2))

    a=ggplot(data=val.list,aes(x=Value,fill=ID))
```

```
    b=scale_x_continuous(limits=c(0,1))
    c=geom_density(alpha=0.6)
    d=theme_classic()
    d2=geom_vline(xintercept = as.numeric(test_val),colour="black",
                  linetype="dashed")
    d3=ggtitle(paste0("Figure A.",i,ii))

    print(a+b+c+d+d2+d3)

    IDs=unique(val.list$ID)

    for(iii in 1:2){
      ID=IDs[iii]
      print(ID)
      sub.val=val.list[which(val.list$ID==ID),]
      mu=mean(sub.val$Value)
      sigma=sd(sub.val$Value)
      n=nrow(sub.val)

      xbar=as.numeric(test_val)

      z=(xbar-mu)/(sigma/sqrt(n))

      lowcrit=qnorm(p=0.025,mean=mu,sd=sigma)
      hicrit=qnorm(p=0.975,mean=mu,sd=sigma)

      if(is.na(lowcrit)){
        print("ERROR: NA values detected.")
        next
      }
      if(is.na(hicrit)){
        print("ERROR: NA values detected.")
        next
      }


      print(paste0("P value for ",sp," vs. ",comp.sp,
                   " = ",pnorm(xbar,
                               mean=mu,sd=sigma)))

      if(xbar<lowcrit){
        print("Test statistic below low critical value.")
        print(paste0(lowcrit,"; statistic = ",xbar))
        next
      }
      if(xbar>hicrit){
        print("Test statistic above high critical value.")
        print(paste0(hicrit,"; statistic = ",xbar))
      }
    }
  }
}
```

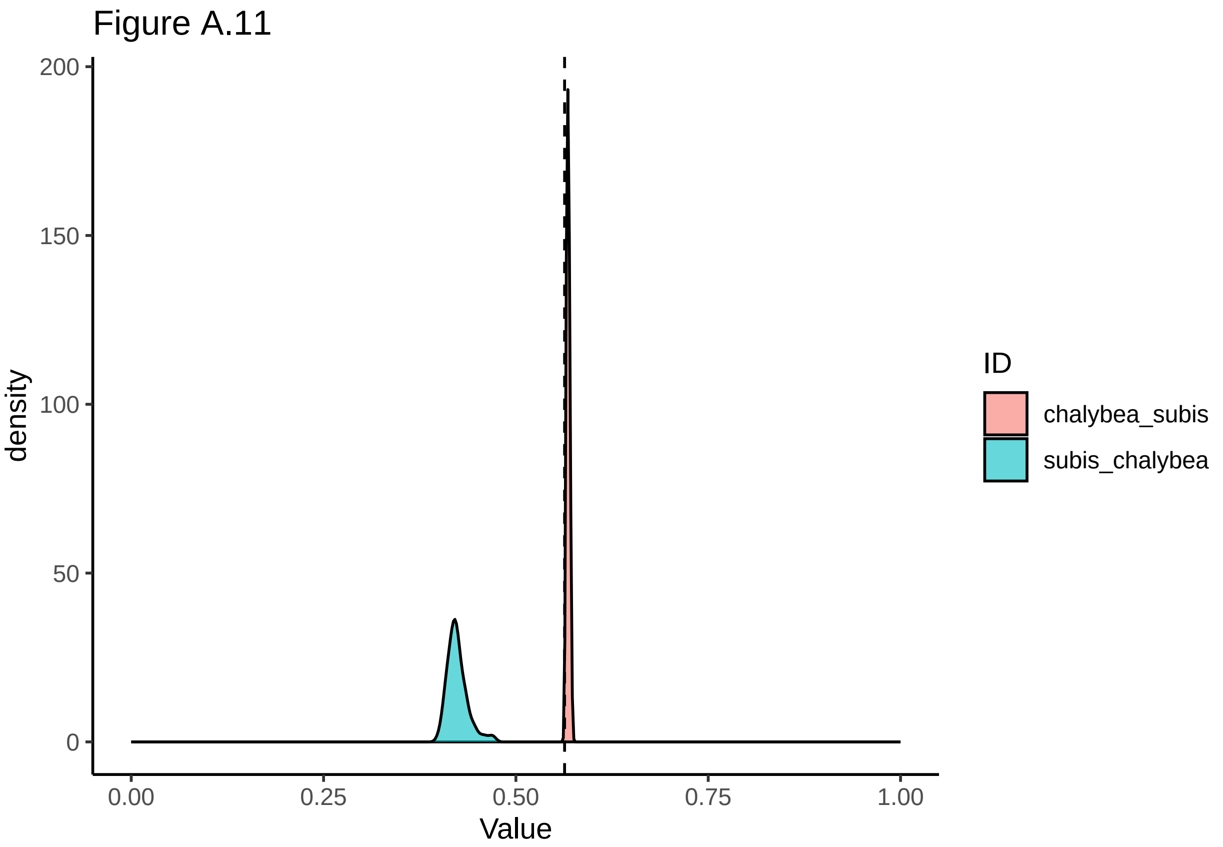


```
## [1] "chalybea_subis"
## [1] "P value for chalybea vs. subis = 0.00874160329770293"
## [1] "Test statistic below low critical value."
## [1] "0.564118732714916; statistic = 0.563313689881235"
## [1] "subis_chalybea"
## [1] "P value for chalybea vs. subis = 1"
## [1] "Test statistic above high critical value."
## [1] "0.449945704057096; statistic = 0.563313689881235"
```

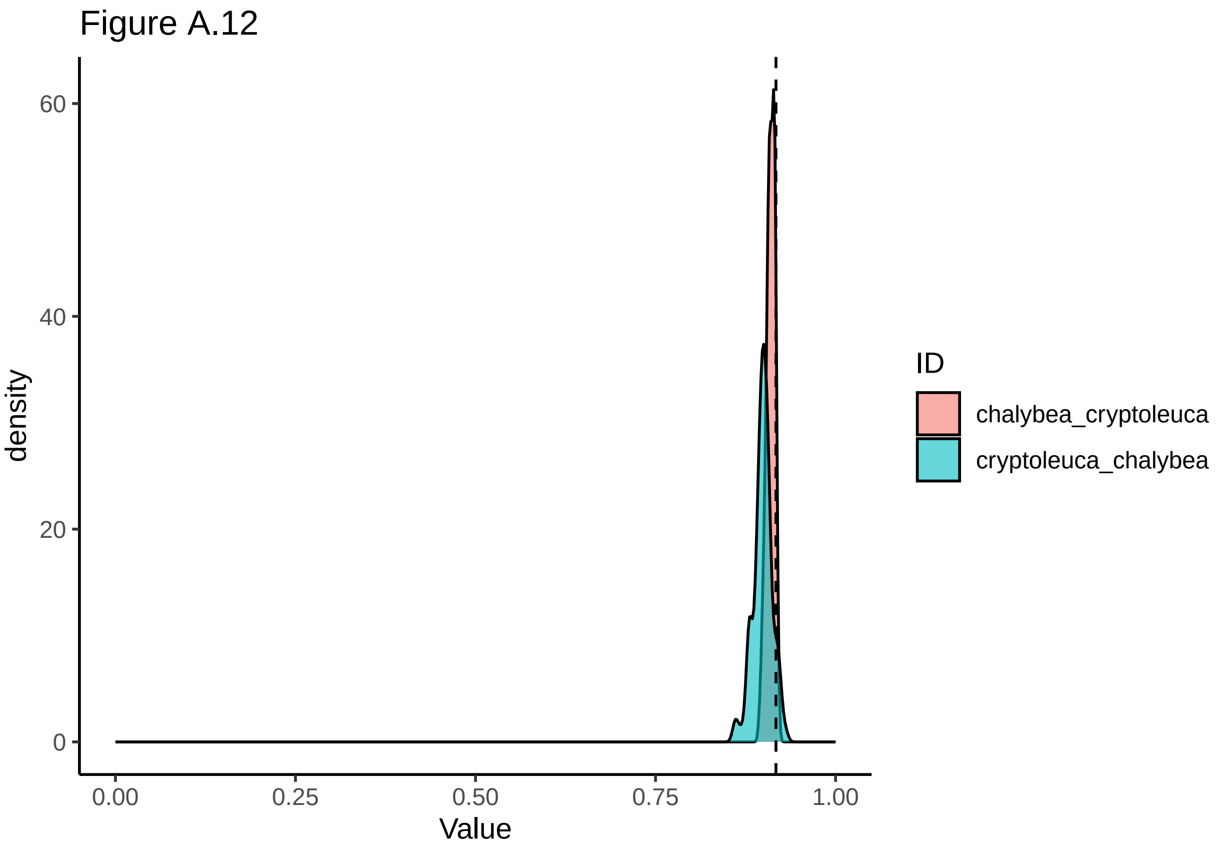


```
## [1] "chalybea_cryptoleuca"
## [1] "P value for chalybea vs. cryptoleuca = 0.905112084577193"
## [1] "cryptoleuca_chalybea"
## [1] "P value for chalybea vs. cryptoleuca = 0.923949851048234"
```

Figure A.13

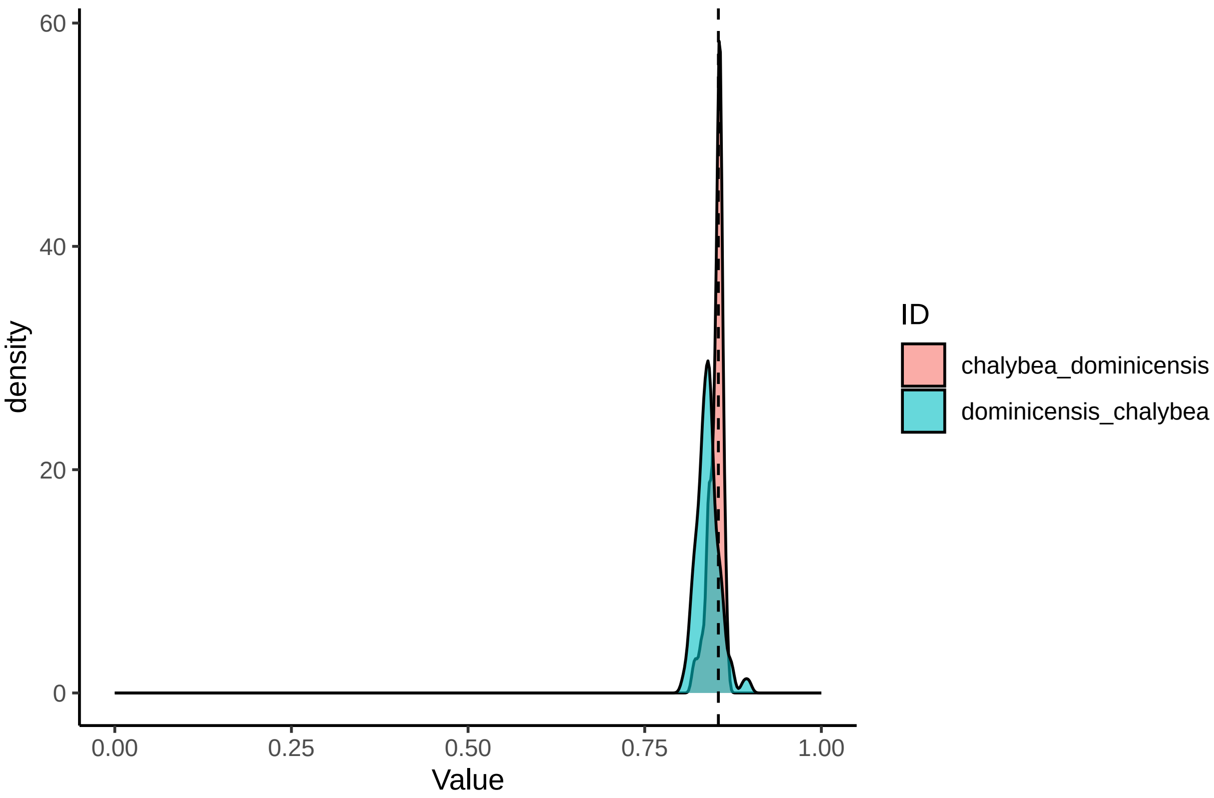


```
## [1] "chalybea_dominicensis"
## [1] "P value for chalybea vs. dominicensis = 0.630975743510612"
## [1] "dominicensis_chalybea"
## [1] "P value for chalybea vs. dominicensis = 0.822508483083474"
```

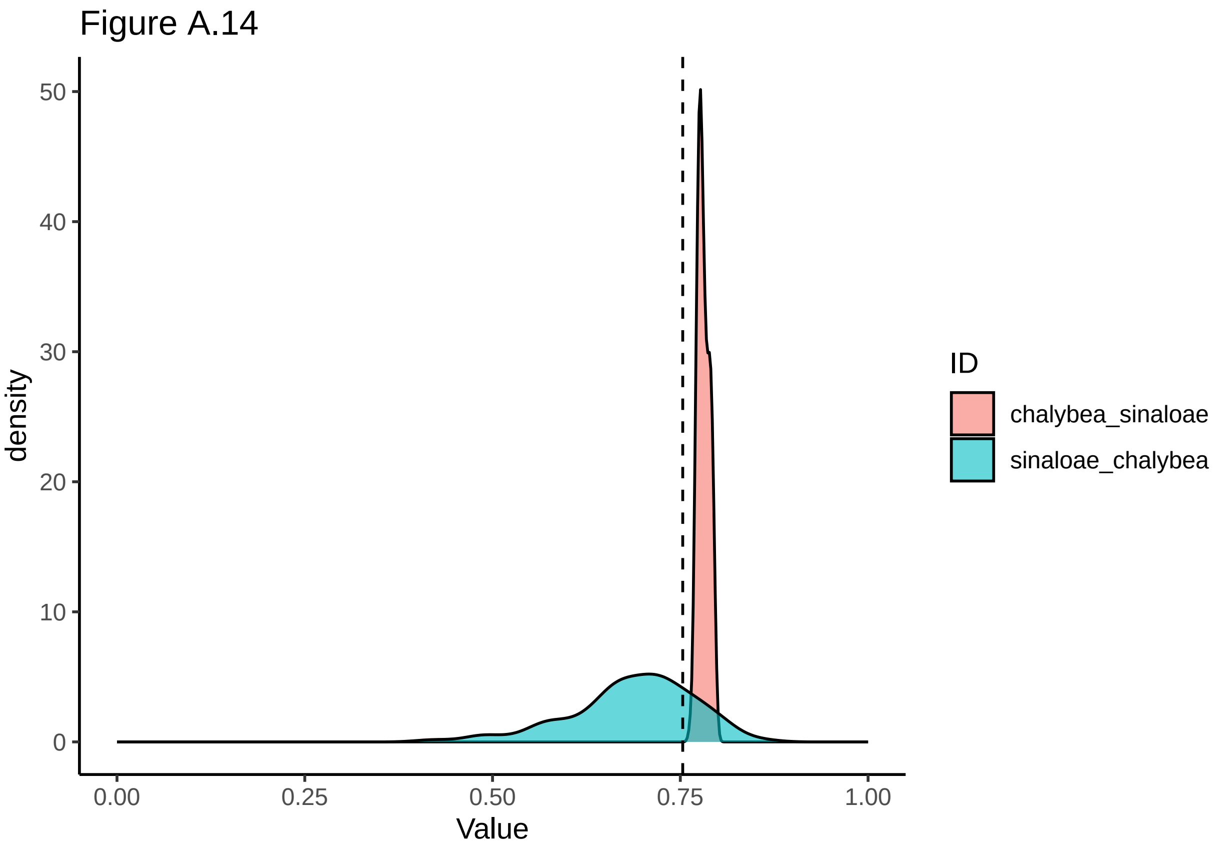


```
## [1] "chalybea_sinaloae"
## [1] "P value for chalybea vs. sinaloae = 0.000142331531800325"
## [1] "Test statistic below low critical value."
## [1] "0.765954690487922; statistic = 0.753219351057136"
## [1] "sinaloae_chalybea"
## [1] "P value for chalybea vs. sinaloae = 0.790200660181079"
```

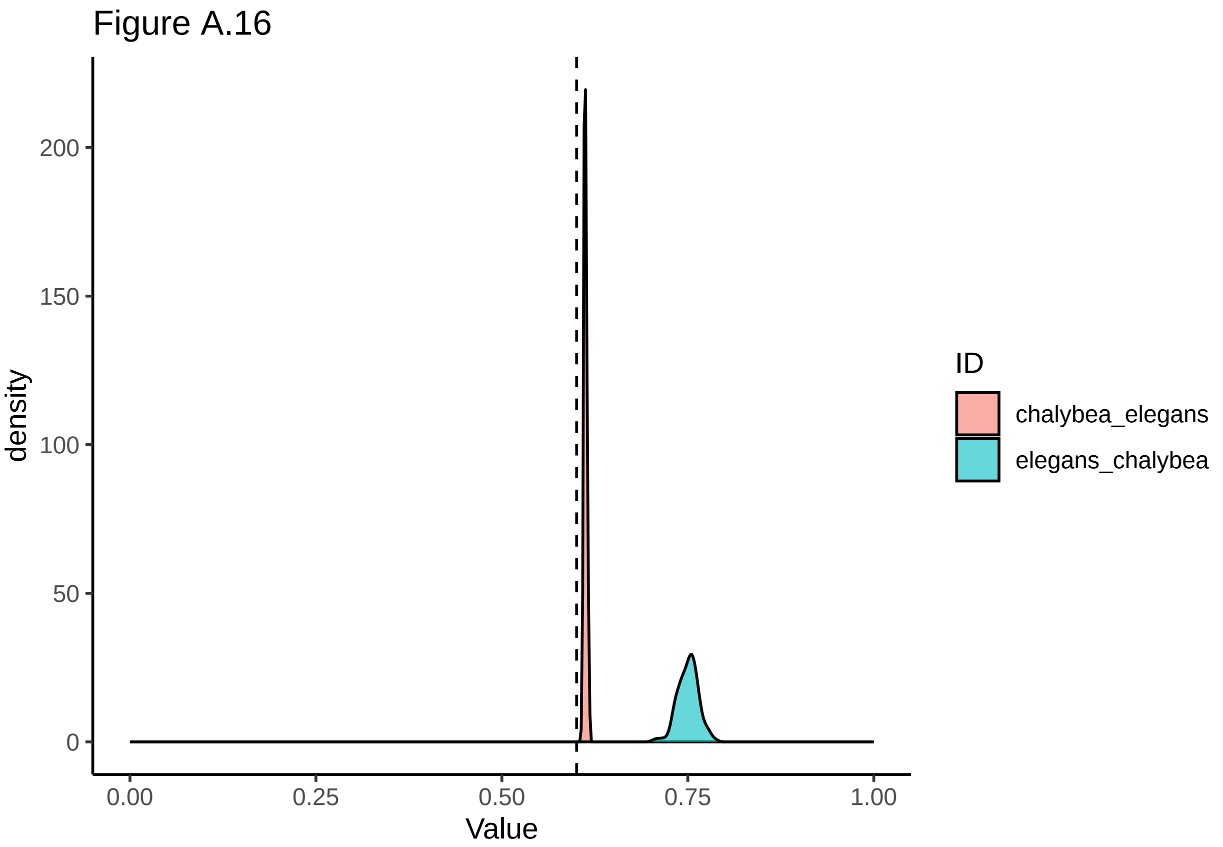


```
## [1] "chalybea_elegans"
## [1] "P value for chalybea vs. elegans = 1.1268673669308e-10"
## [1] "Test statistic below low critical value."
## [1] "0.608673933067226; statistic = 0.600536008828745"
## [1] "elegans_chalybea"
## [1] "P value for chalybea vs. elegans = 3.06799382017121e-28"
## [1] "Test statistic below low critical value."
## [1] "0.723481951512536; statistic = 0.600536008828745"
```

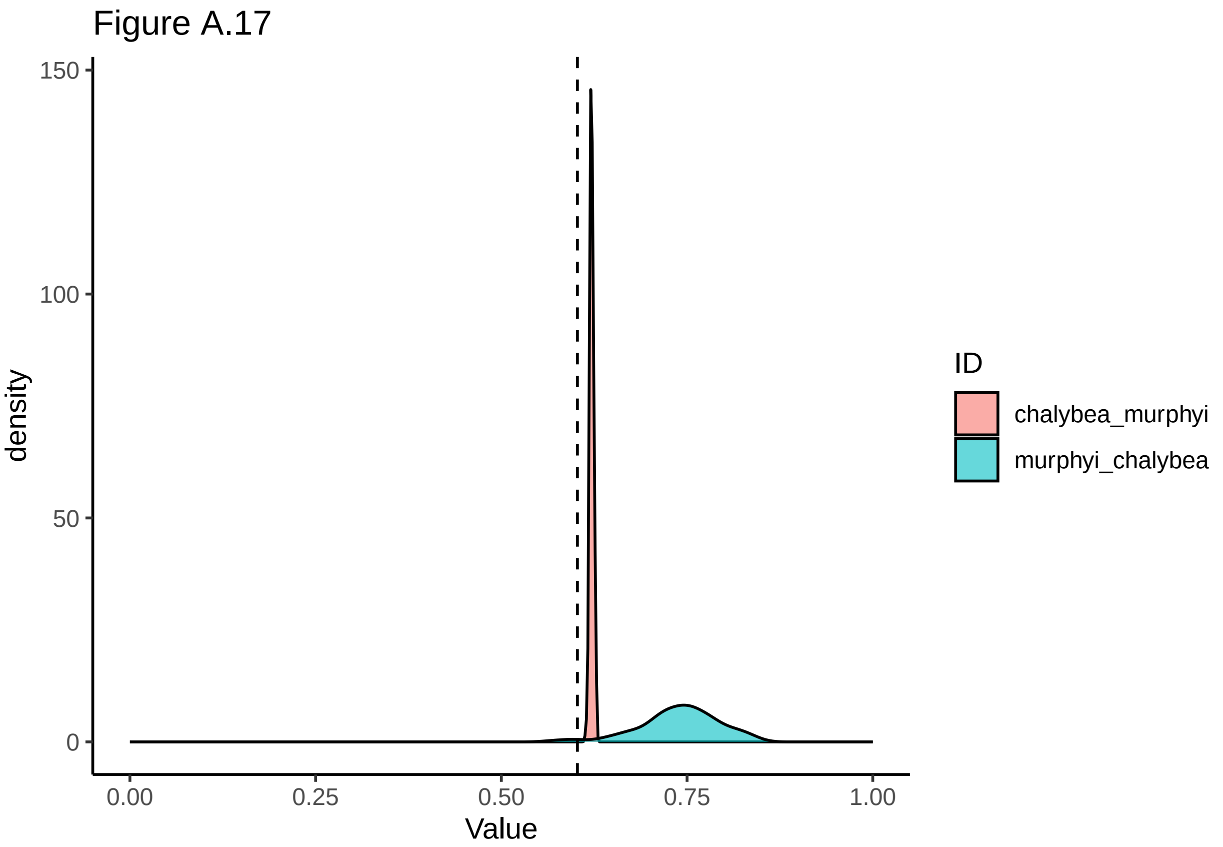


```
## [1] "chalybea_murphyi"
## [1] "P value for chalybea vs. murphyi = 2.63731972570716e-15"
## [1] "Test statistic below low critical value."
## [1] "0.616780352612352; statistic = 0.60239972901452"
## [1] "murphyi_chalybea"
## [1] "P value for chalybea vs. murphyi = 0.00437438262692634"
## [1] "Test statistic below low critical value."
## [1] "0.637154158297721; statistic = 0.60239972901452"
```

Figure A.18

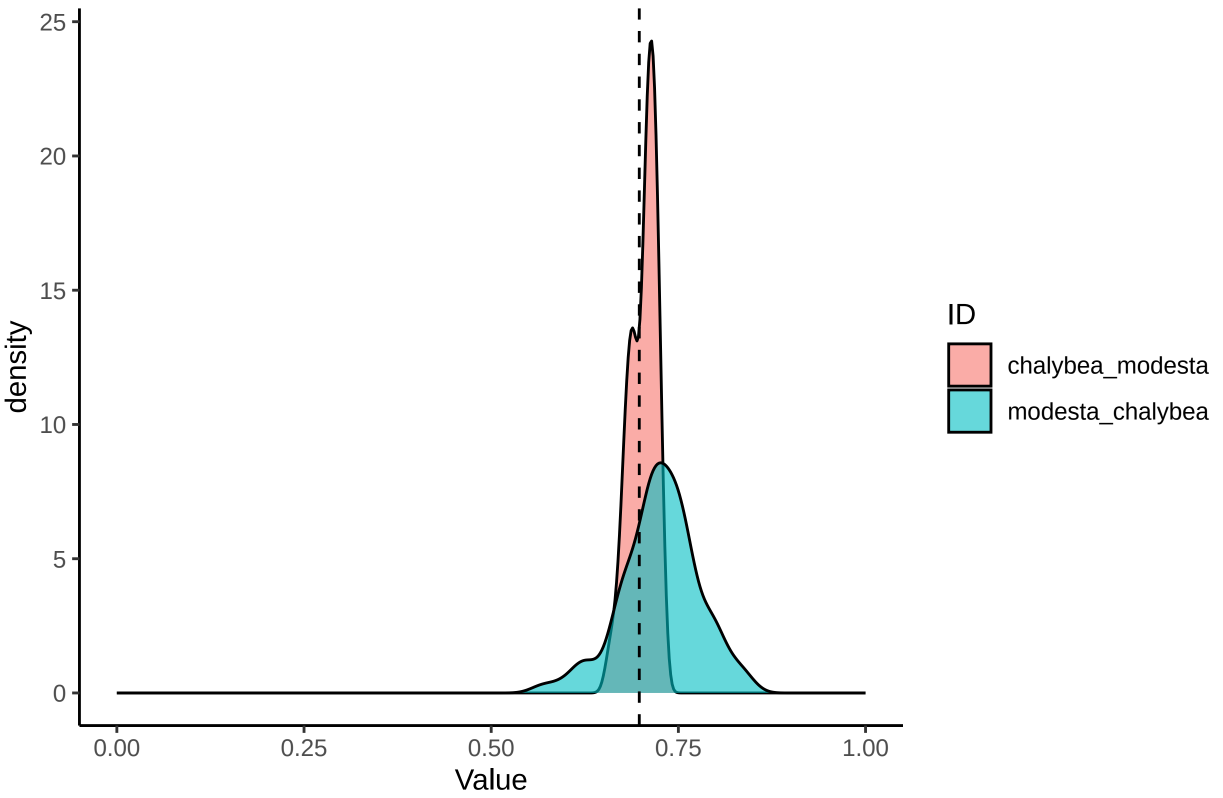


```
## [1] "chalybea_modesta"
## [1] "P value for chalybea vs. modesta = 0.389634581657439"
## [1] "modesta_chalybea"
## [1] "P value for chalybea vs. modesta = 0.294789673096894"
```

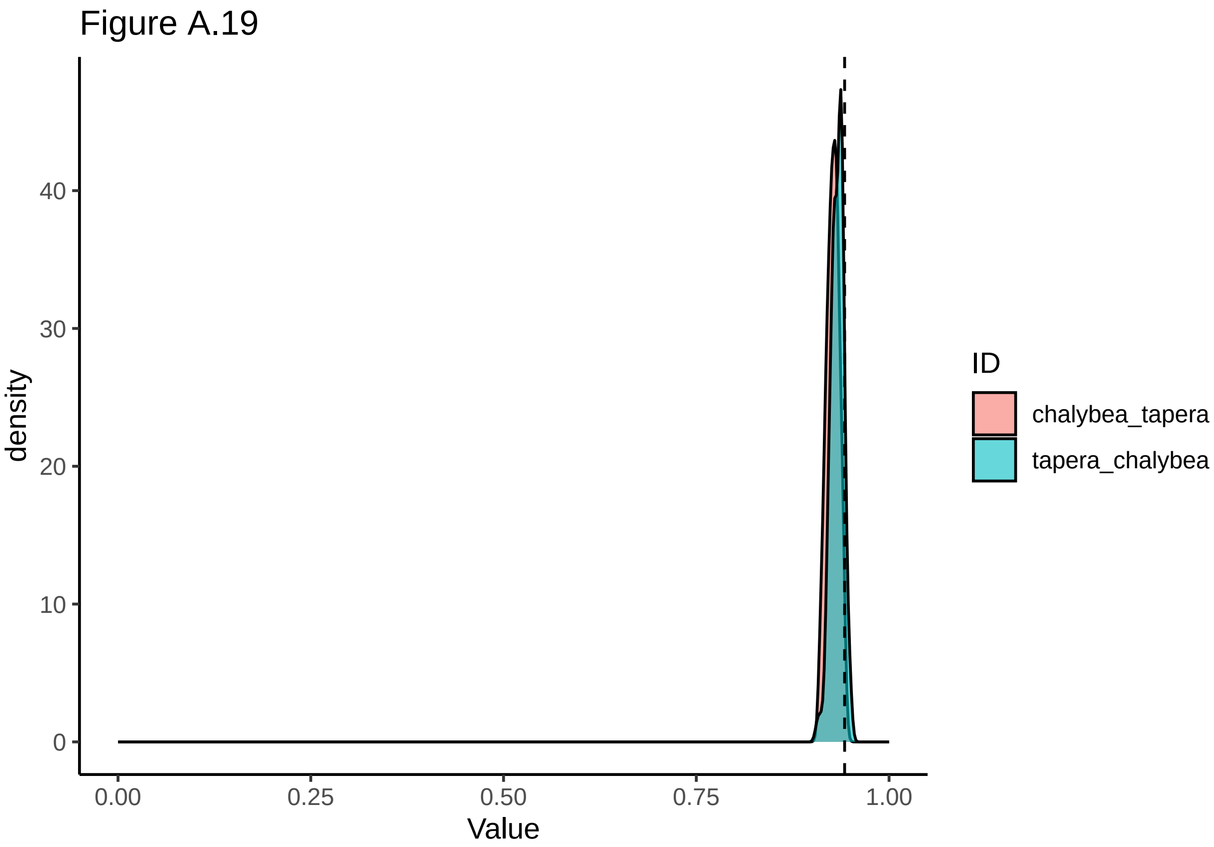


```
## [1] "chalybea_tapera"
## [1] "P value for chalybea vs. tapera = 0.972516514513643"
## [1] "tapera_chalybea"
## [1] "P value for chalybea vs. tapera = 0.886159914910166"
```

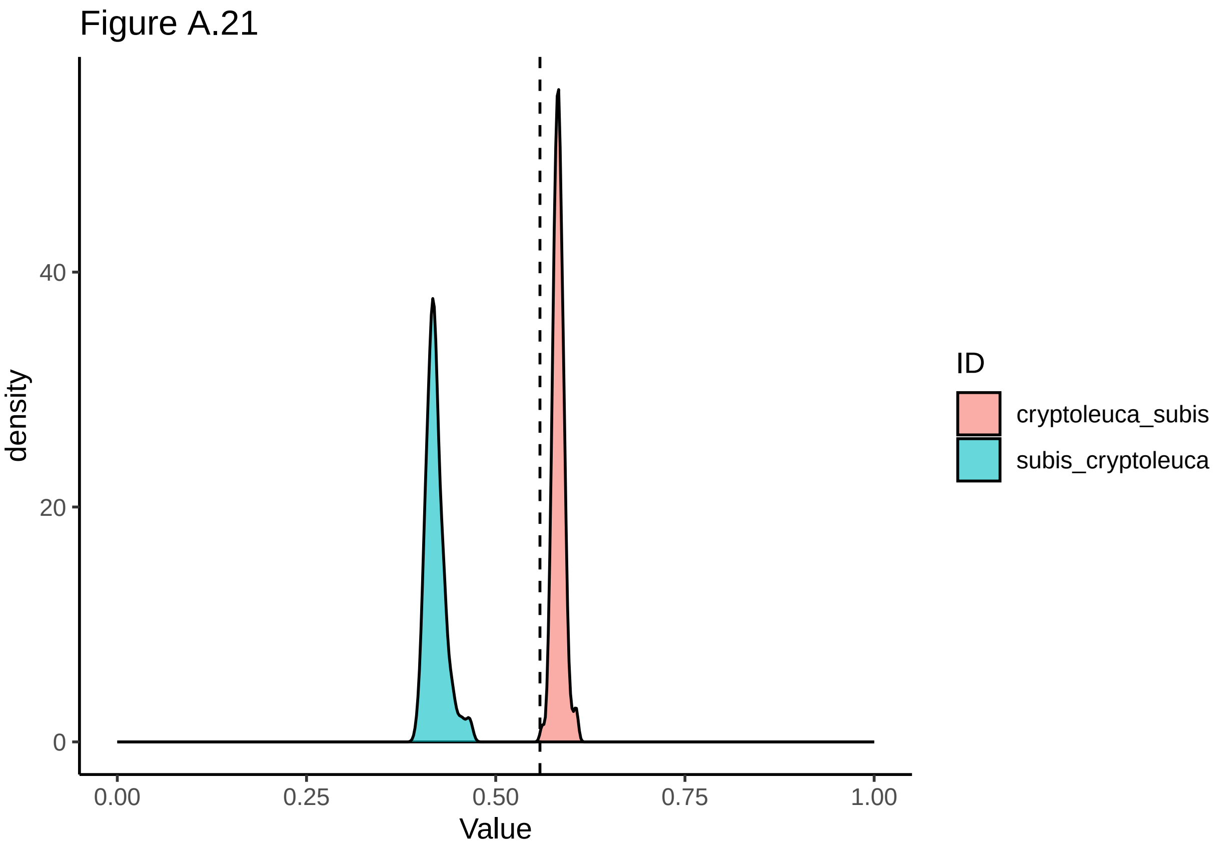


```
## [1] "cryptoleuca_subis"
## [1] "P value for cryptoleuca vs. subis = 0.000451736307097324"
## [1] "Test statistic below low critical value."
## [1] "0.568482313562568; statistic = 0.558444925984436"
## [1] "subis_cryptoleuca"
## [1] "P value for cryptoleuca vs. subis = 1"
## [1] "Test statistic above high critical value."
## [1] "0.446182291694113; statistic = 0.558444925984436"
```

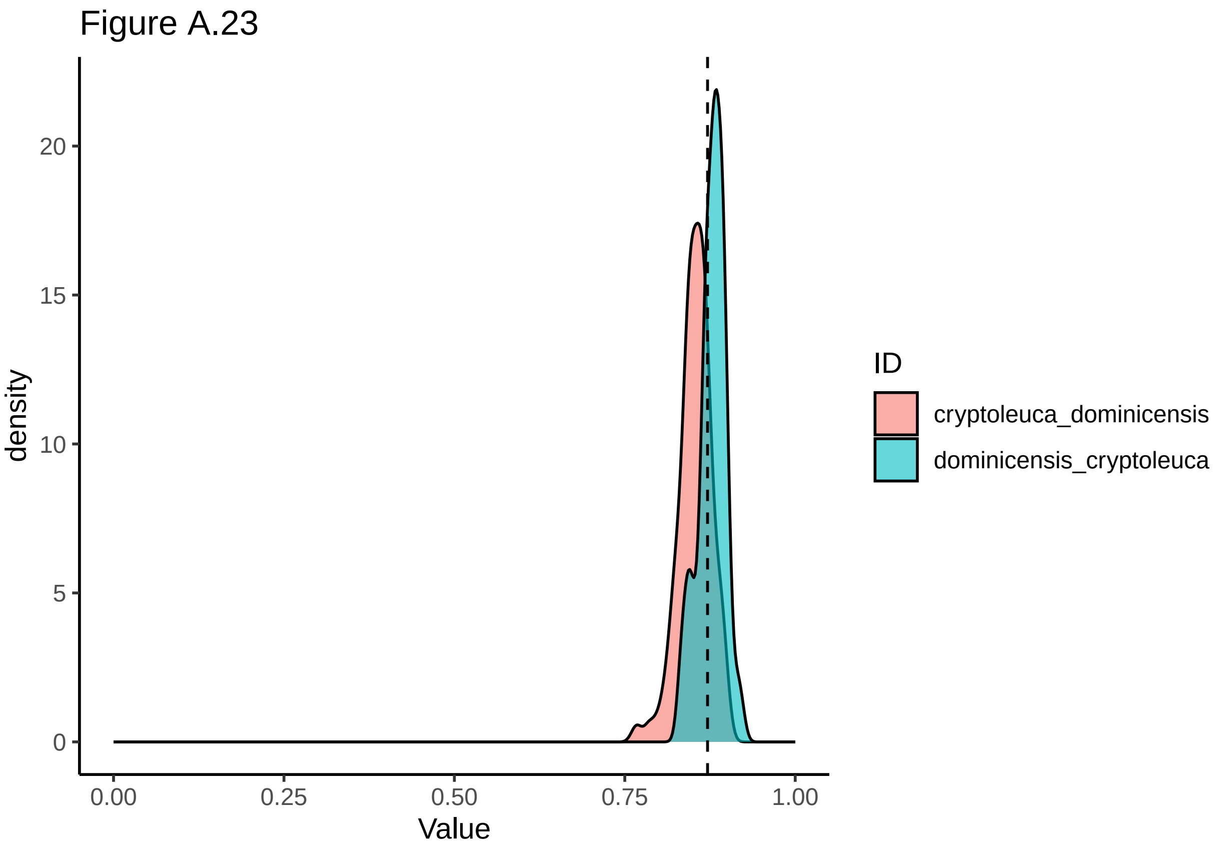


```
## [1] "cryptoleuca_dominicensis"
## [1] "P value for cryptoleuca vs. dominicensis = 0.779539496090278"
## [1] "dominicensis_cryptoleuca"
## [1] "P value for cryptoleuca vs. dominicensis = 0.369108677163184"
```

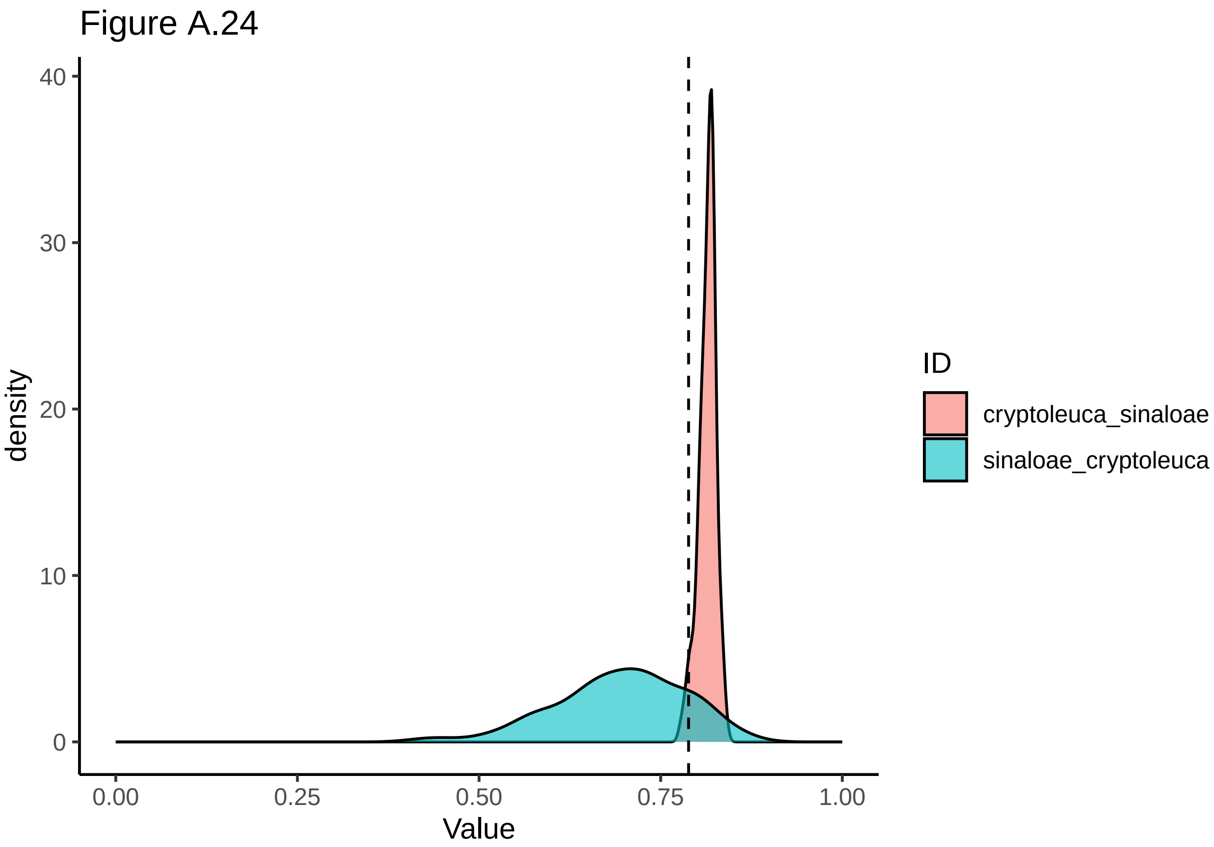


```
## [1] "cryptoleuca_sinaloae"
## [1] "P value for cryptoleuca vs. sinaloae = 0.013094146575736"
## [1] "Test statistic below low critical value."
## [1] "0.791481323974456; statistic = 0.788410073141191"
## [1] "sinaloae_cryptoleuca"
## [1] "P value for cryptoleuca vs. sinaloae = 0.854437149651724"
```

Figure A.25

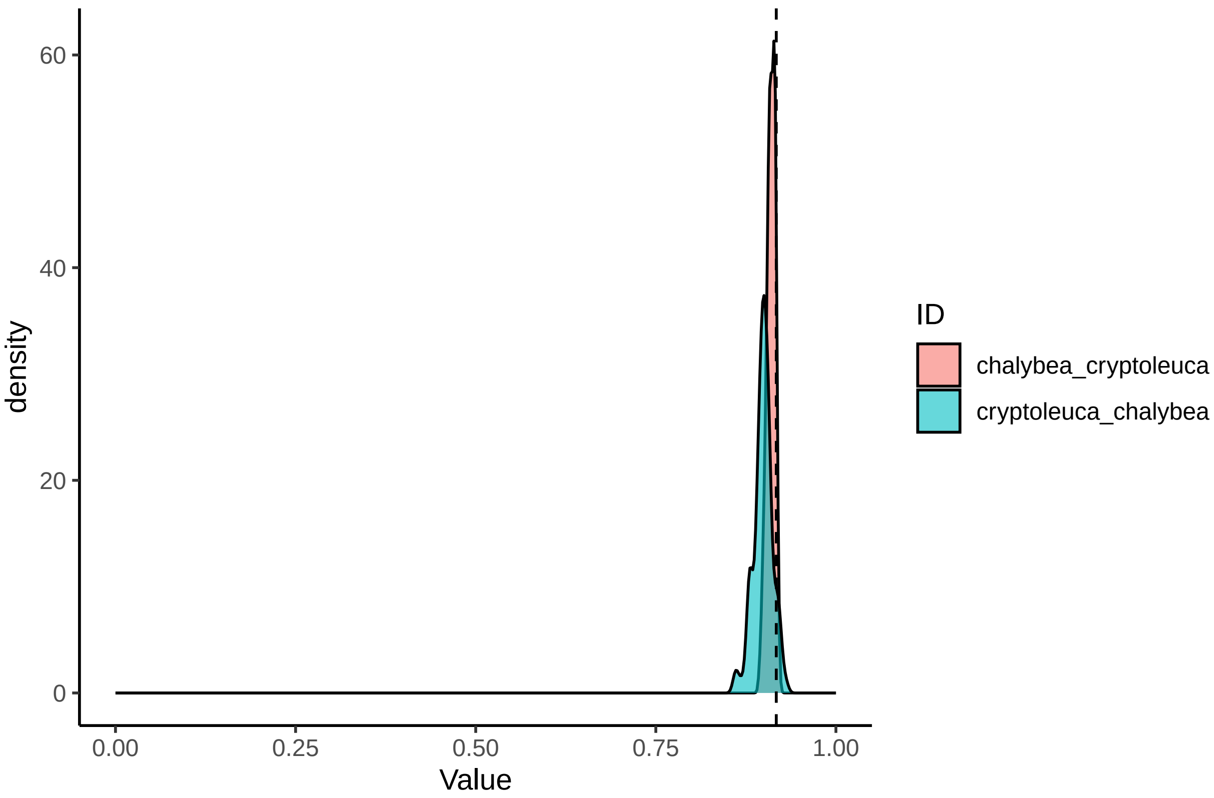


```
## [1] "cryptoleuca_chalybea"
## [1] "P value for cryptoleuca vs. chalybea = 0.923949851048234"
## [1] "chalybea_cryptoleuca"
## [1] "P value for cryptoleuca vs. chalybea = 0.905112084577193"
```

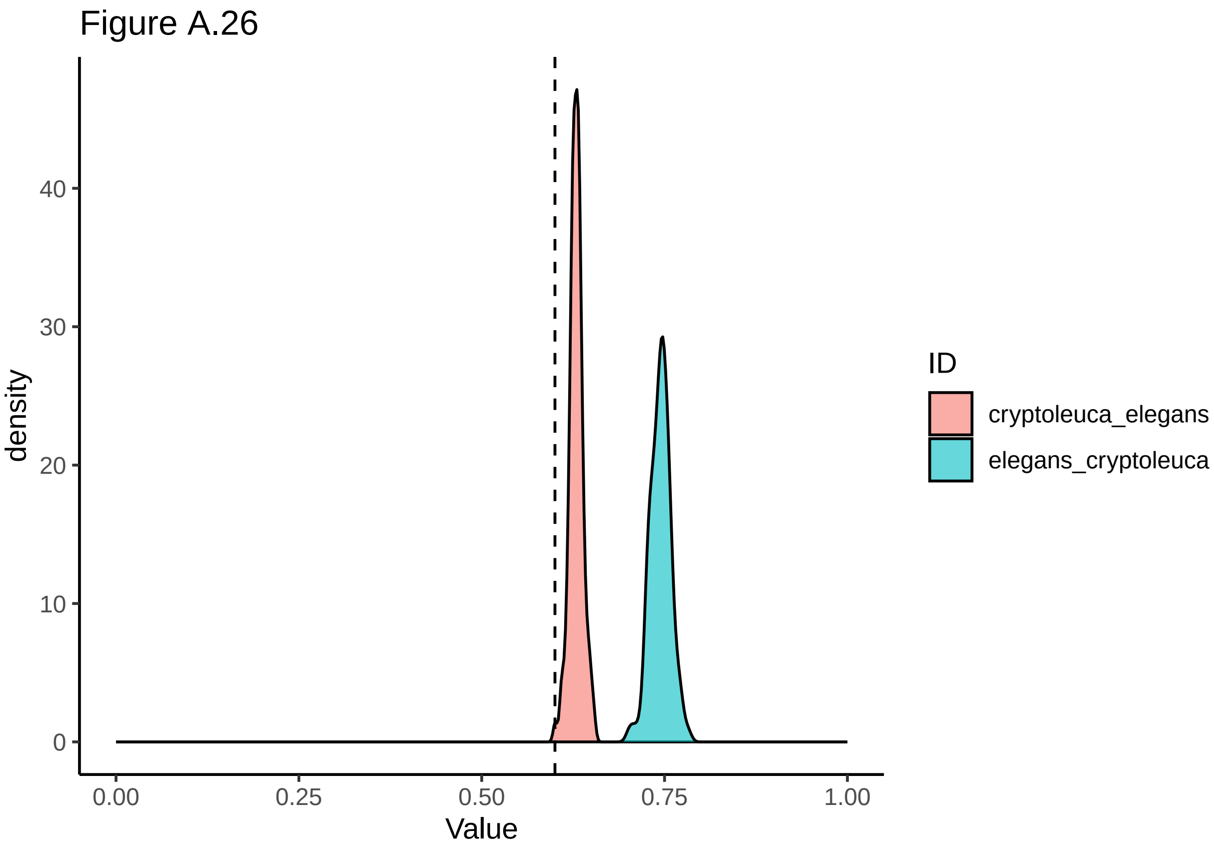


```
## [1] "cryptoleuca_elegans"
## [1] "P value for cryptoleuca vs. elegans = 0.00061633324265485"
## [1] "Test statistic below low critical value."
## [1] "0.611546019196136; statistic = 0.600134558565549"
## [1] "elegans_cryptoleuca"
## [1] "P value for cryptoleuca vs. elegans = 4.89834878089961e-26"
## [1] "Test statistic below low critical value."
## [1] "0.717360041838917; statistic = 0.600134558565549"
```

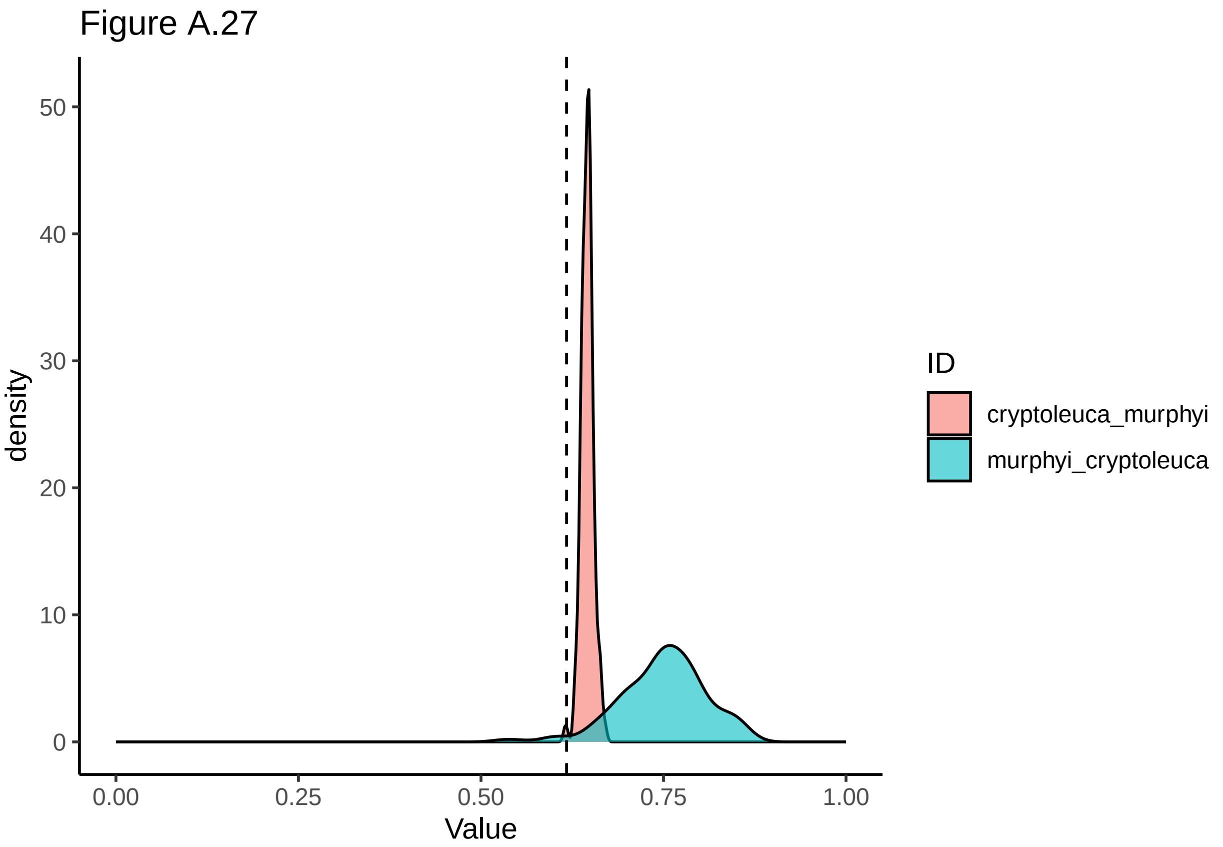


```
## [1] "cryptoleuca_murphyi"
## [1] "P value for cryptoleuca vs. murphyi = 0.000307285776401752"
## [1] "Test statistic below low critical value."
## [1] "0.629372014246327; statistic = 0.617227887259557"
## [1] "murphyi_cryptoleuca"
## [1] "P value for cryptoleuca vs. murphyi = 0.0109638774769042"
## [1] "Test statistic below low critical value."
## [1] "0.636418105864818; statistic = 0.617227887259557"
```

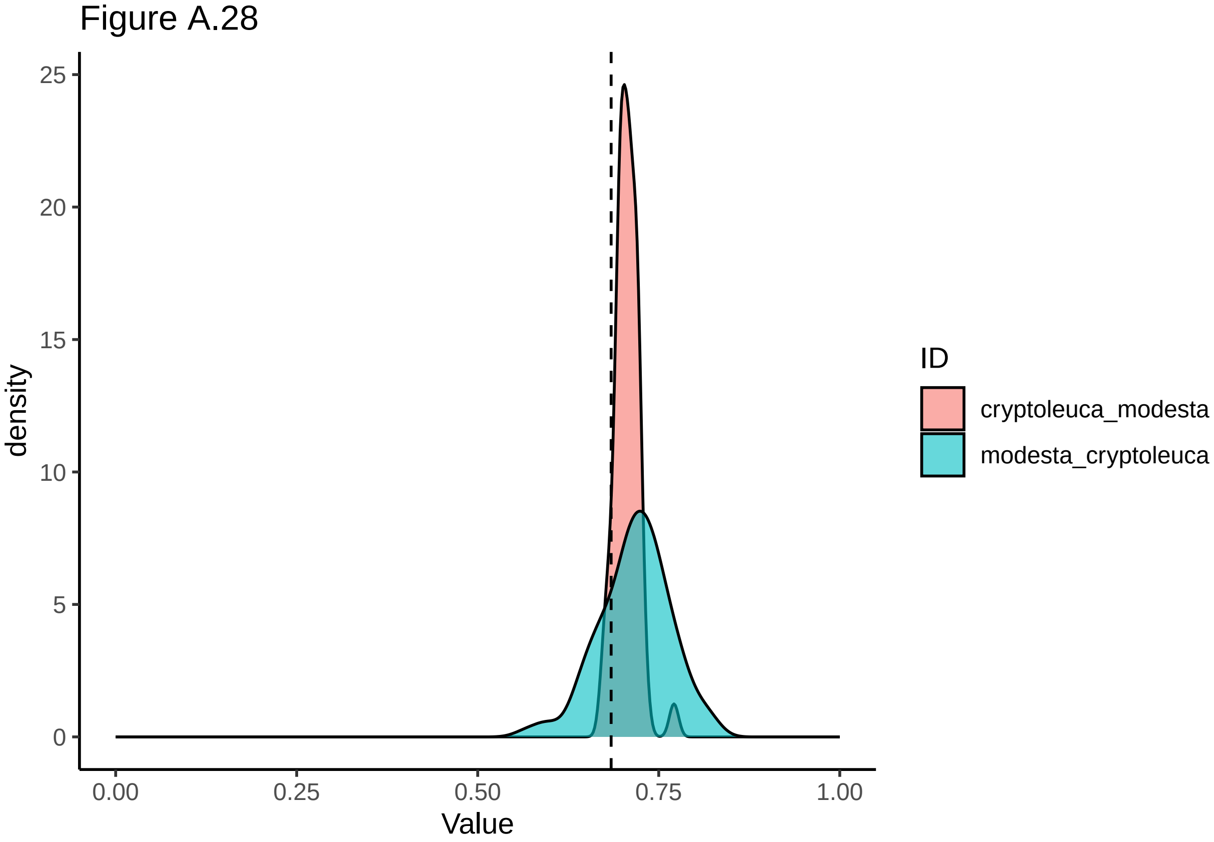


```
## [1] "cryptoleuca_modesta"
## [1] "P value for cryptoleuca vs. modesta = 0.092096150558271"
## [1] "modesta_cryptoleuca"
## [1] "P value for cryptoleuca vs. modesta = 0.251702770103792"
```

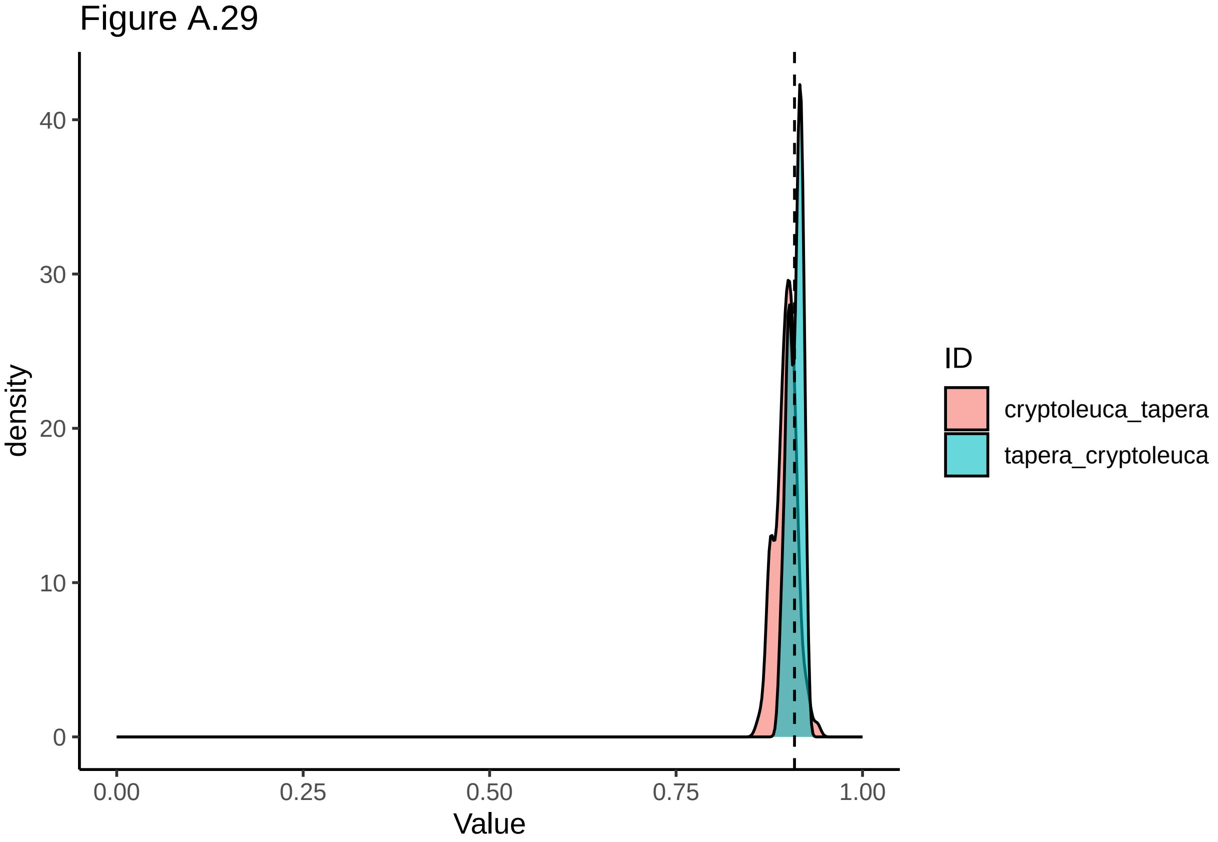


```
## [1] "cryptoleuca_tapera"
## [1] "P value for cryptoleuca vs. tapera = 0.793627816390721"
## [1] "tapera_cryptoleuca"
## [1] "P value for cryptoleuca vs. tapera = 0.454750151764007"
```

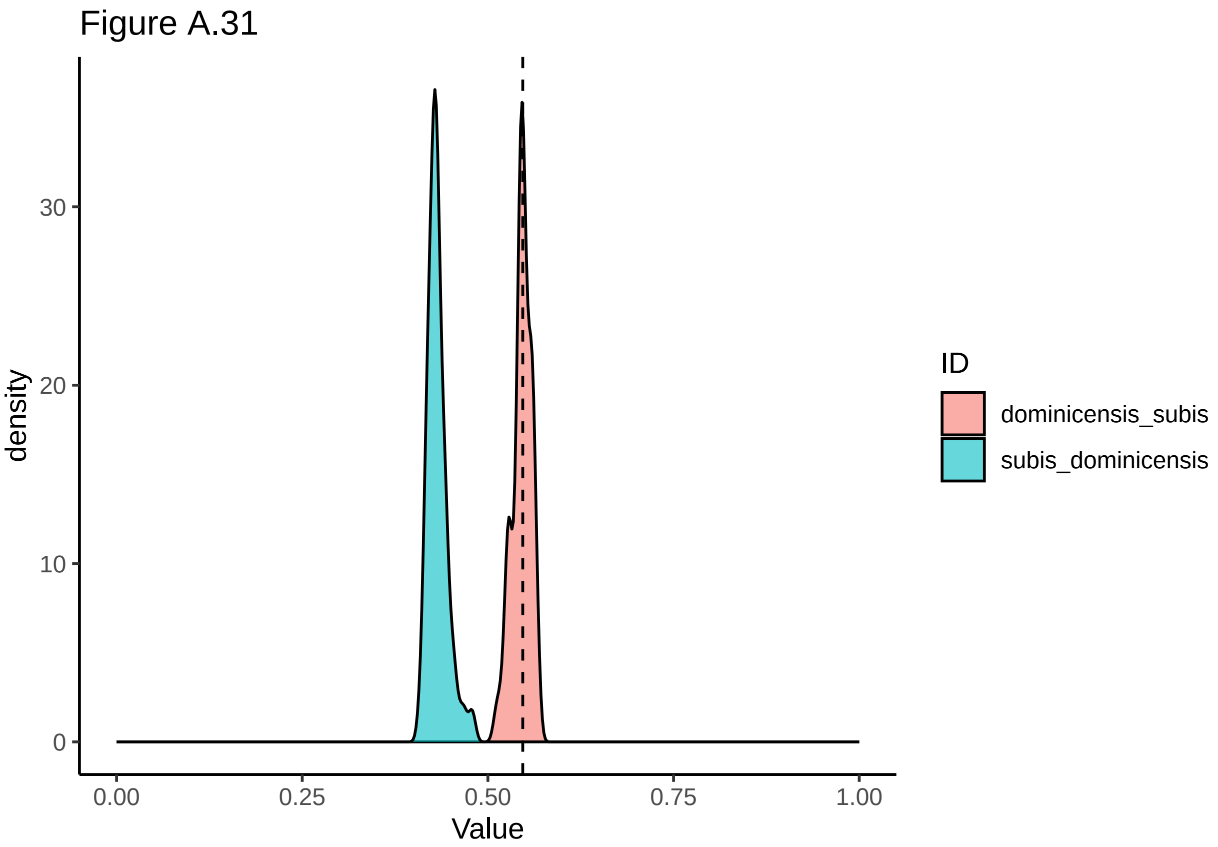


```
## [1] "dominicensis_subis"
## [1] "P value for dominicensis vs. subis = 0.541592052165962"
## [1] "subis_dominicensis"
## [1] "P value for dominicensis vs. subis = 1"
## [1] "Test statistic above high critical value."
## [1] "0.458350885803299; statistic = 0.546931108217863"
```

Figure A.32

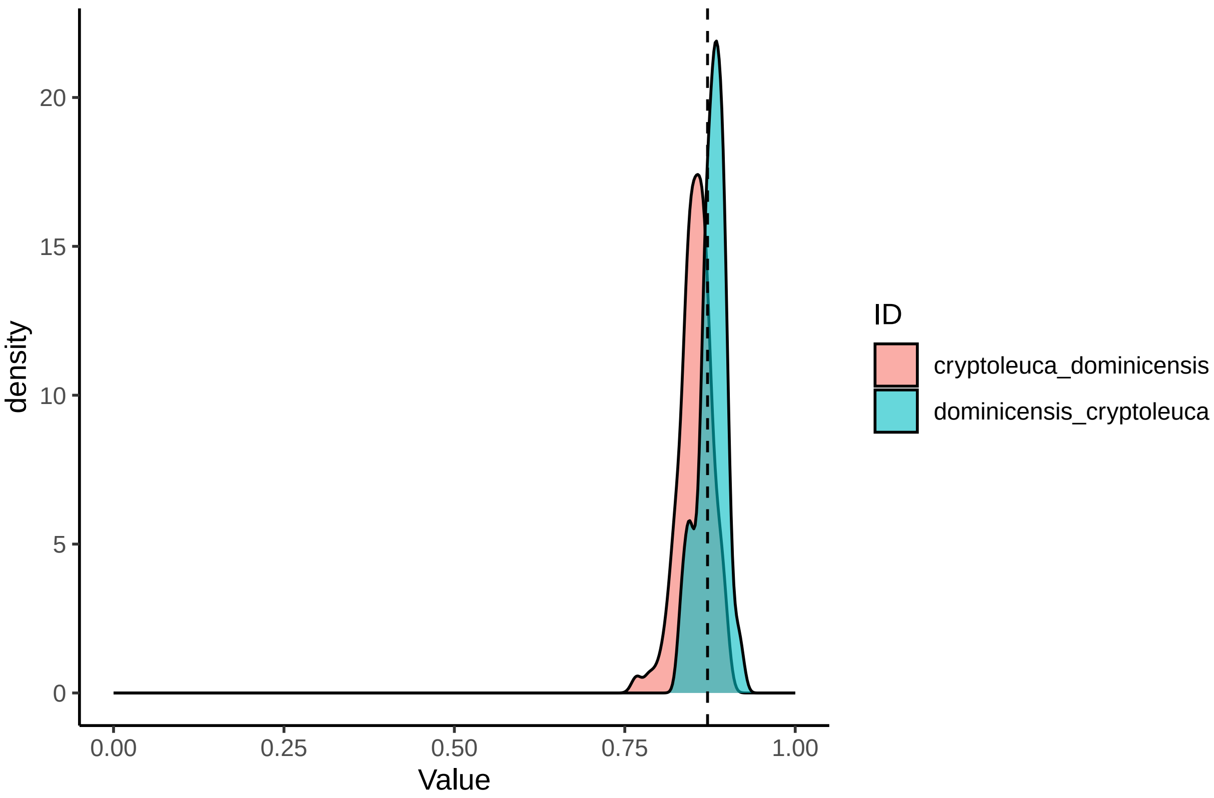


```
## [1] "dominicensis_cryptoleuca"
## [1] "P value for dominicensis vs. cryptoleuca = 0.369108677163184"
## [1] "cryptoleuca_dominicensis"
## [1] "P value for dominicensis vs. cryptoleuca = 0.779539496090278"
```

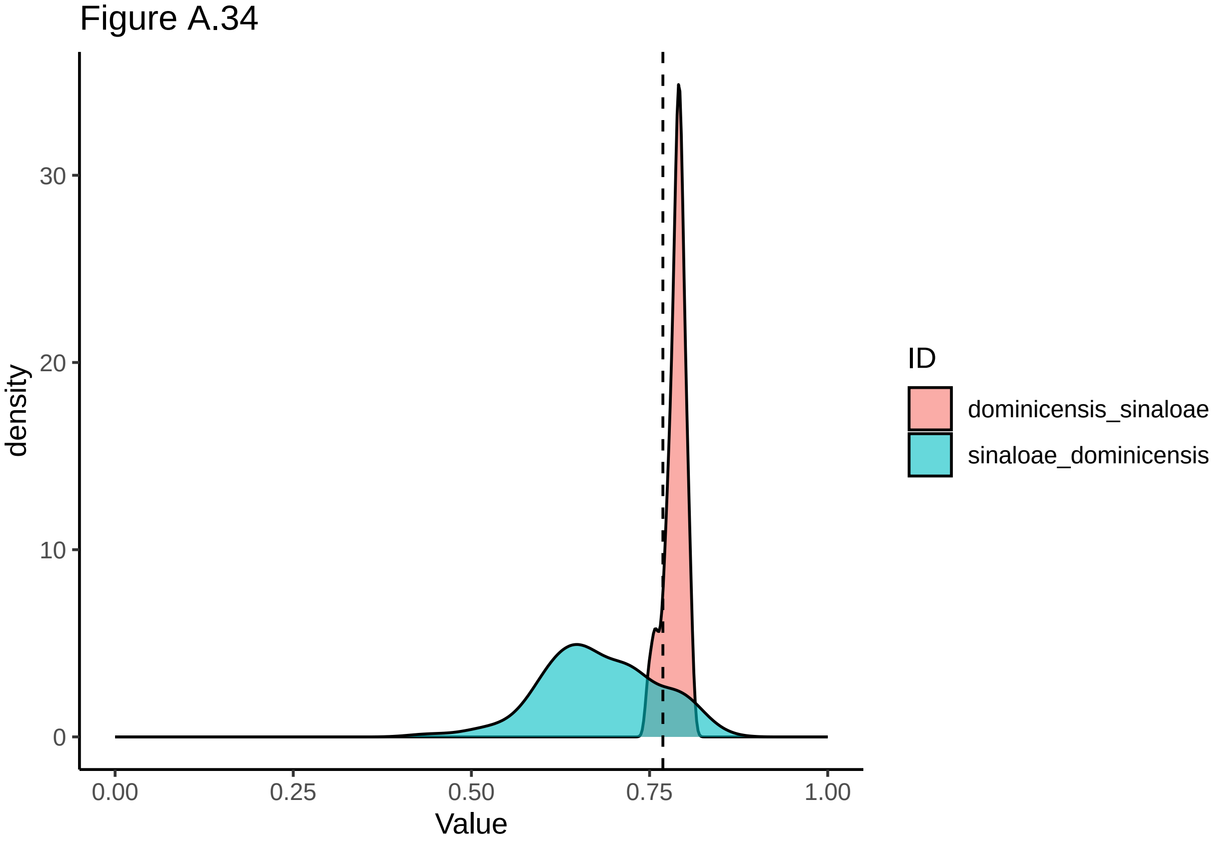


```
## [1] "dominicensis_sinaloae"
## [1] "P value for dominicensis vs. sinaloae = 0.107596749406277"
## [1] "sinaloae_dominicensis"
## [1] "P value for dominicensis vs. sinaloae = 0.873365587875063"
```

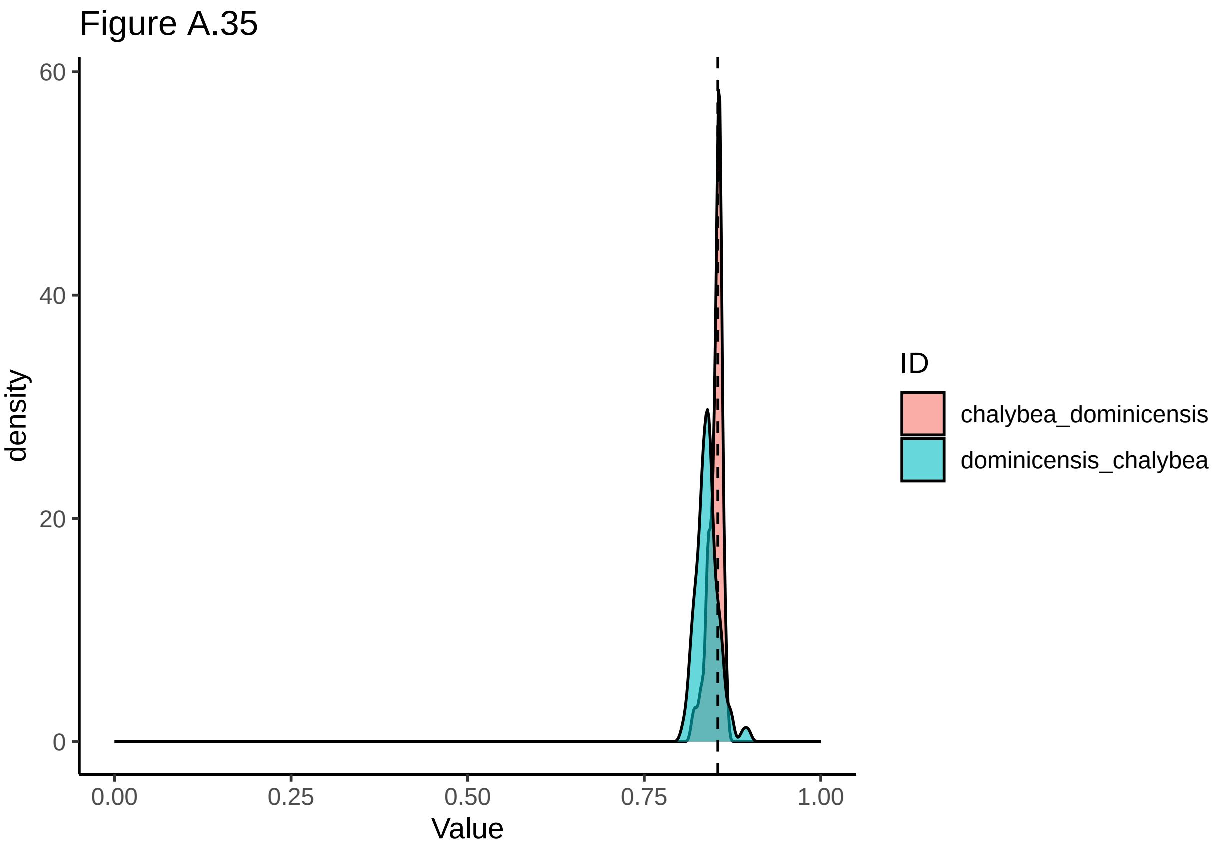


```
## [1] "dominicensis_chalybea"
## [1] "P value for dominicensis vs. chalybea = 0.822508483083474"
## [1] "chalybea_dominicensis"
## [1] "P value for dominicensis vs. chalybea = 0.630975743510612"
```

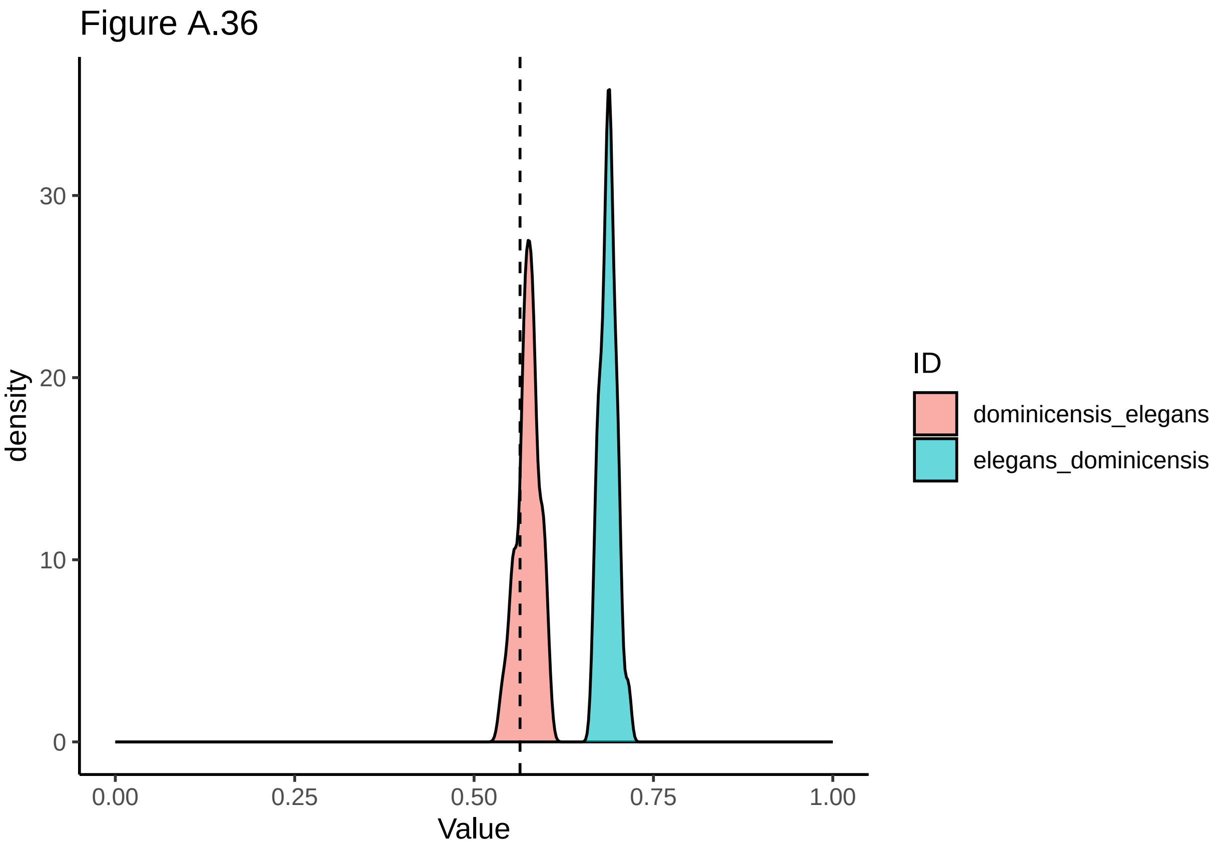


```
## [1] "dominicensis_elegans"
## [1] "P value for dominicensis vs. elegans = 0.234480173997123"
## [1] "elegans_dominicensis"
## [1] "P value for dominicensis vs. elegans = 2.41081677418463e-28"
## [1] "Test statistic below low critical value."
## [1] "0.665239752965937; statistic = 0.564055703589358"
```

Figure A.37

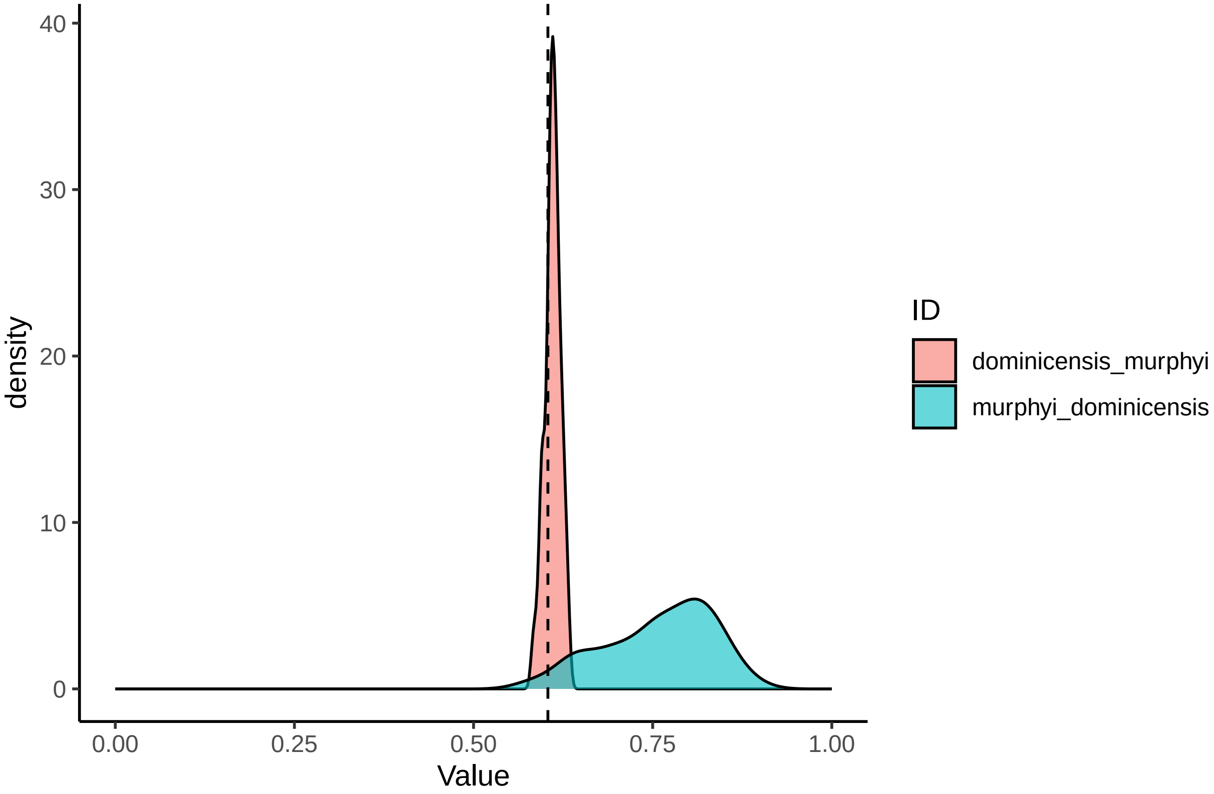


```
## [1] "dominicensis_murphyi"
## [1] "P value for dominicensis vs. murphyi = 0.269514182507919"
## [1] "murphyi_dominicensis"
## [1] "P value for dominicensis vs. murphyi = 0.0192757153826417"
## [1] "Test statistic below low critical value."
## [1] "0.61194638994795; statistic = 0.603802914312001"
```

Figure A.38

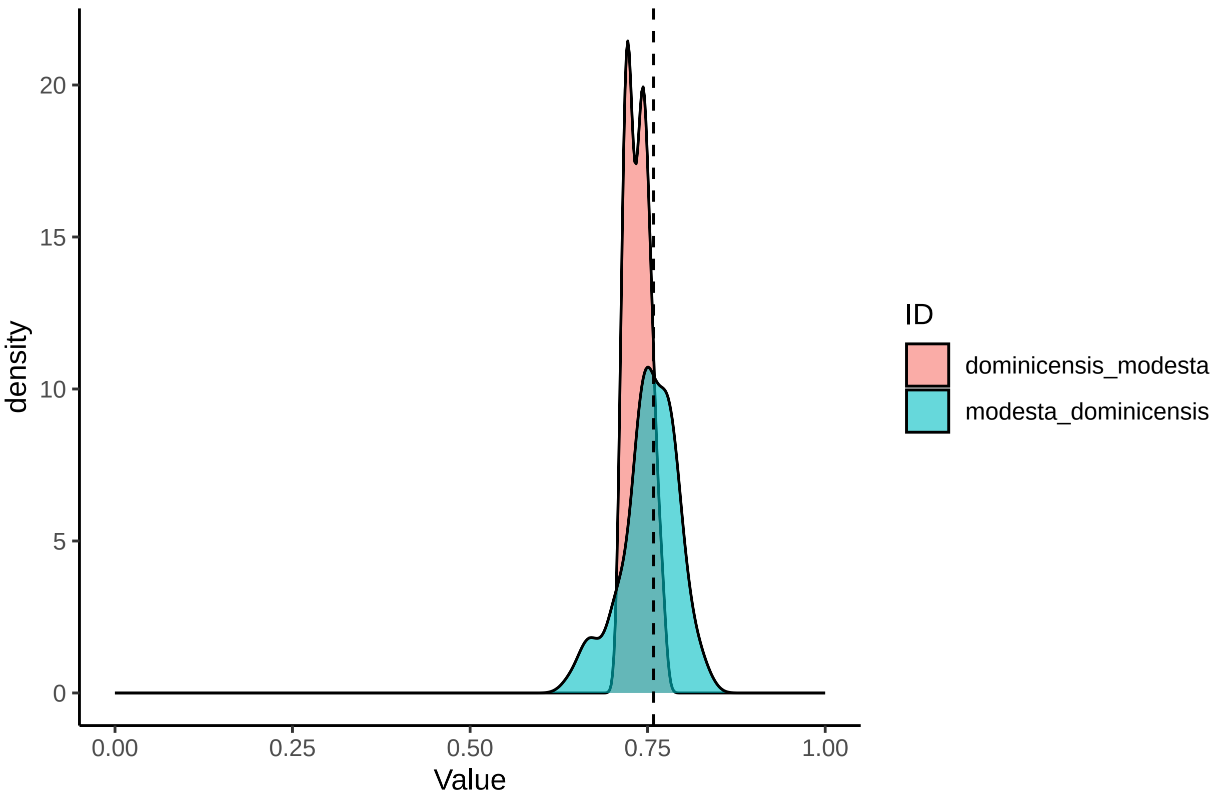


```
## [1] "dominicensis_modesta"
## [1] "P value for dominicensis vs. modesta = 0.912132791878"
## [1] "modesta_dominicensis"
## [1] "P value for dominicensis vs. modesta = 0.560153596534554"
```

Figure A.39

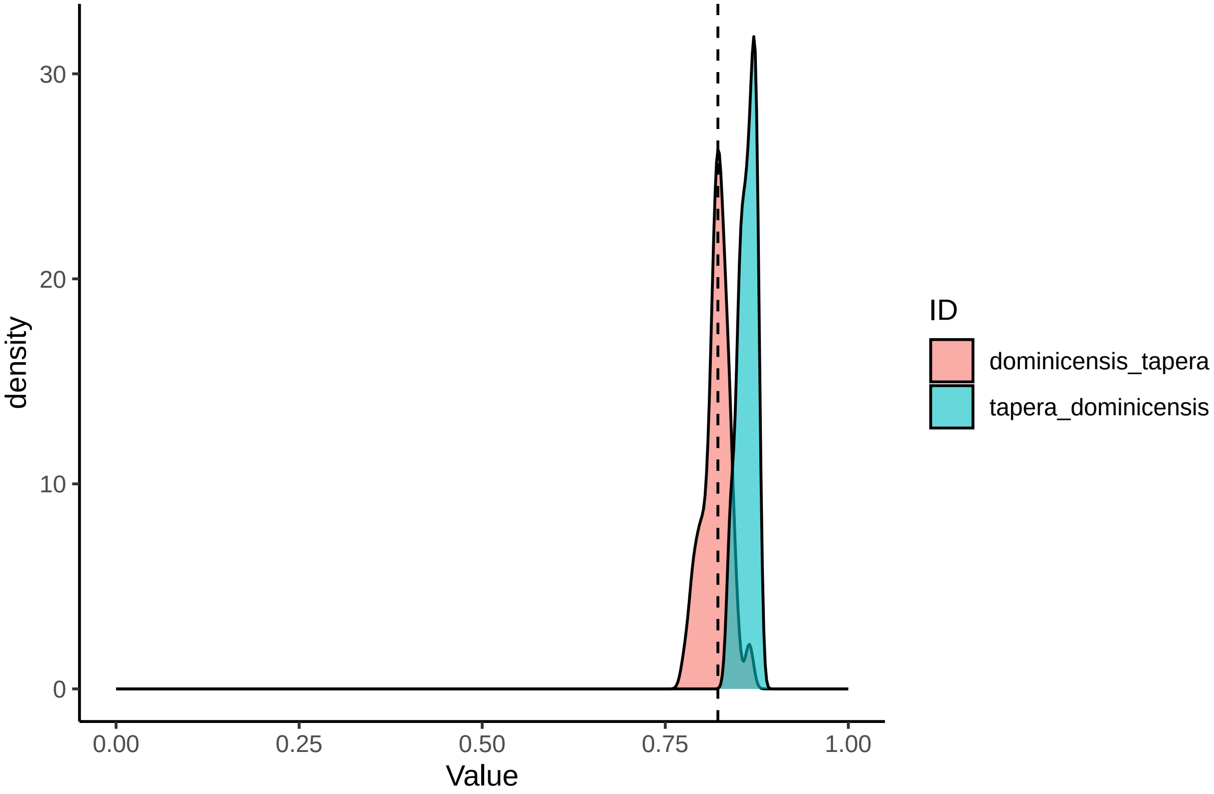


```
## [1] "dominicensis_tapera"
## [1] "P value for dominicensis vs. tapera = 0.53343858396198"
## [1] "tapera_dominicensis"
## [1] "P value for dominicensis vs. tapera = 0.000351440909227224"
## [1] "Test statistic below low critical value."
## [1] "0.83855636419014; statistic = 0.821881855815174"
```

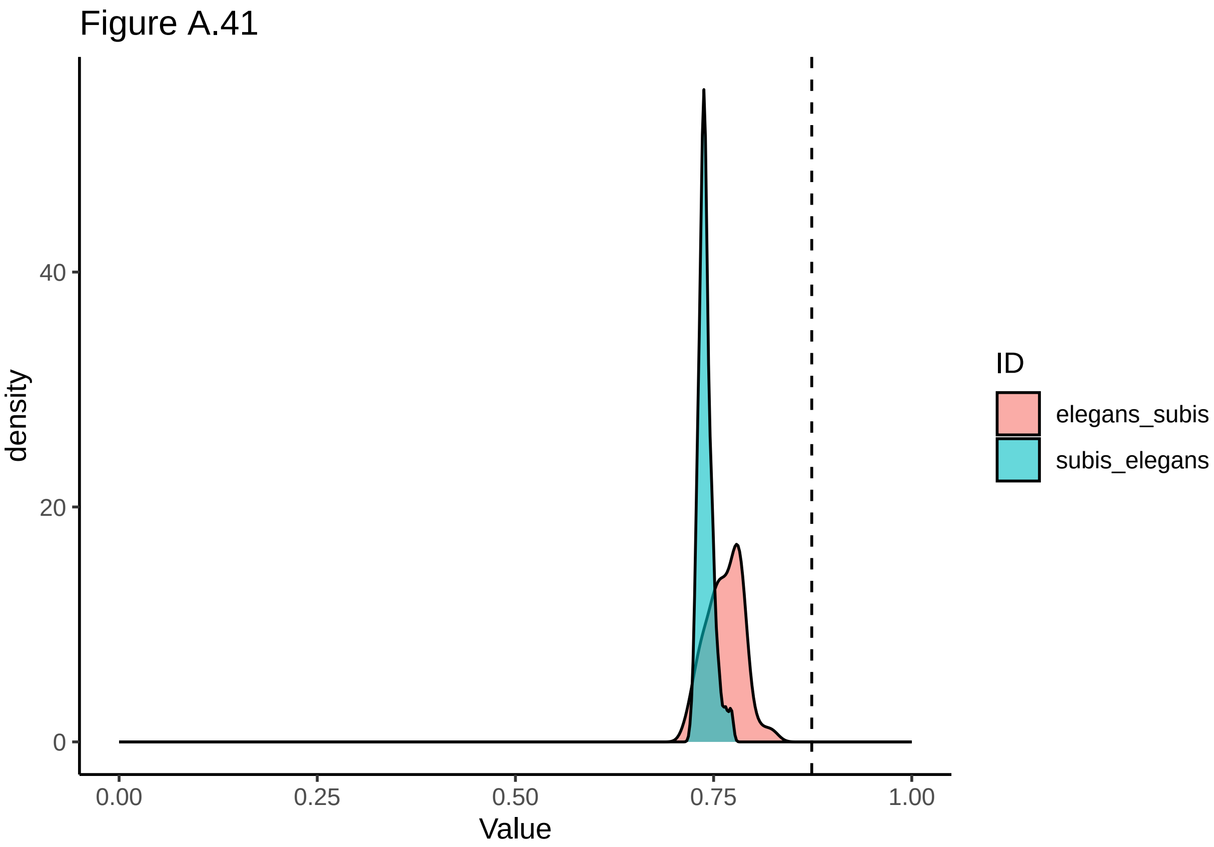


```
## [1] "elegans_subis"
## [1] "P value for elegans vs. subis = 0.999999380362749"
## [1] "Test statistic above high critical value."
## [1] "0.808541951133876; statistic = 0.873820627396604"
## [1] "subis_elegans"
## [1] "P value for elegans vs. subis = 1"
## [1] "Test statistic above high critical value."
## [1] "0.758251033354595; statistic = 0.873820627396604"
```

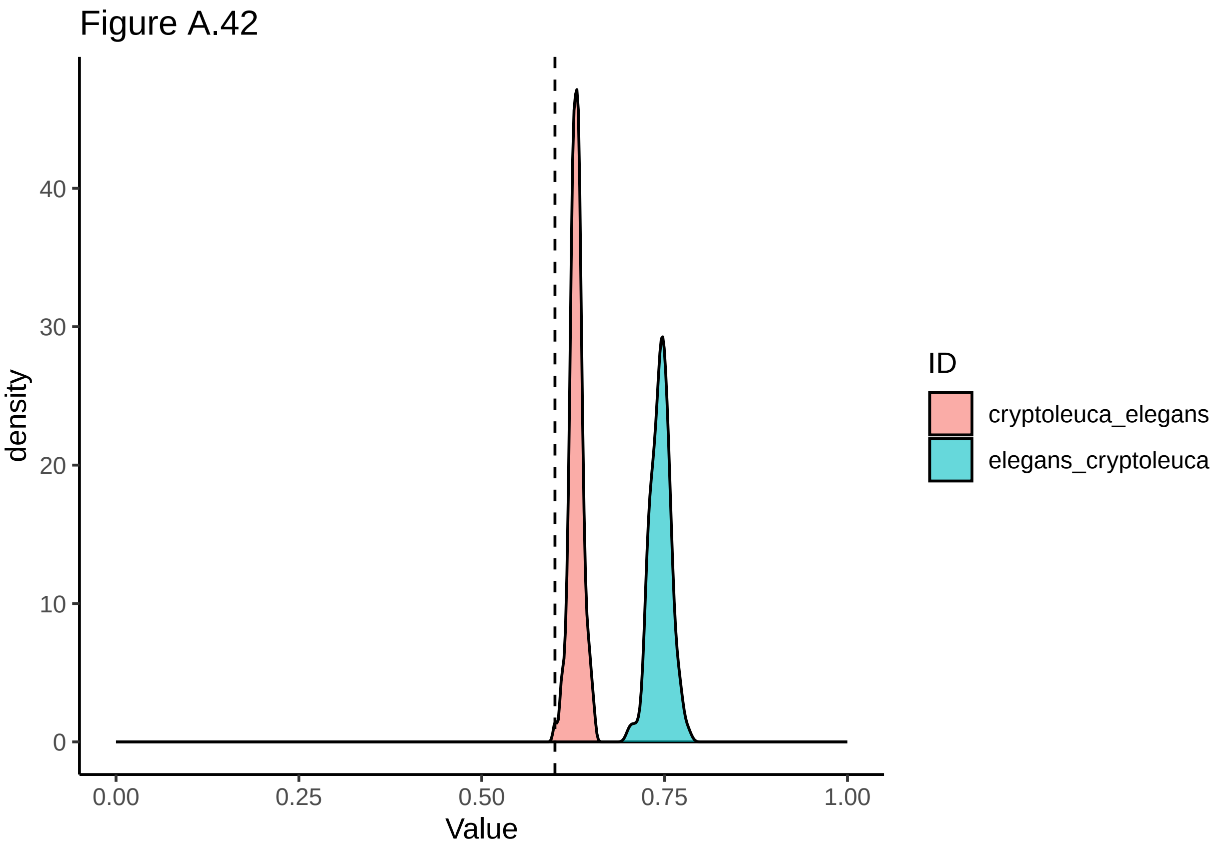


```
## [1] "elegans_cryptoleuca"
## [1] "P value for elegans vs. cryptoleuca = 4.89834878089961e-26"
## [1] "Test statistic below low critical value."
## [1] "0.717360041838917; statistic = 0.600134558565549"
## [1] "cryptoleuca_elegans"
## [1] "P value for elegans vs. cryptoleuca = 0.00061633324265485"
## [1] "Test statistic below low critical value."
## [1] "0.611546019196136; statistic = 0.600134558565549"
```

Figure A.43

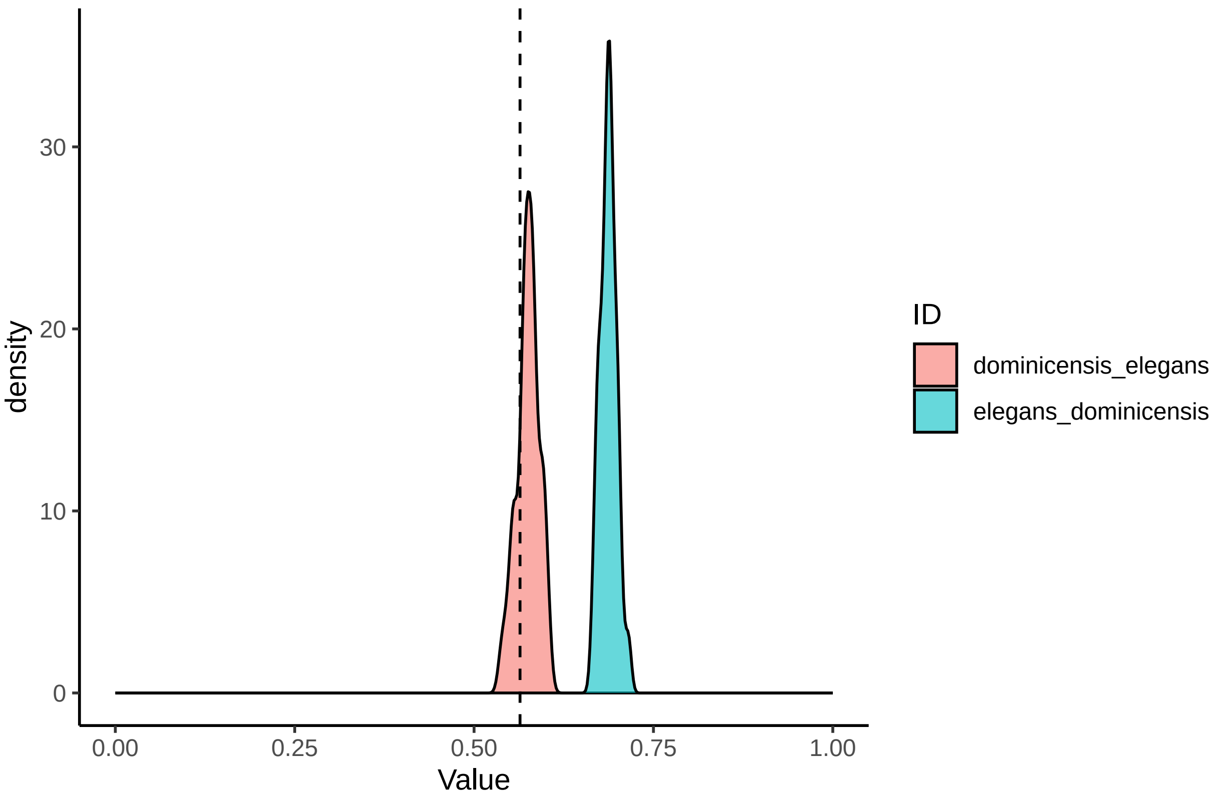


```
## [1] "elegans_dominicensis"
## [1] "P value for elegans vs. dominicensis = 2.41081677418463e-28"
## [1] "Test statistic below low critical value."
## [1] "0.665239752965937; statistic = 0.564055703589358"
## [1] "dominicensis_elegans"
## [1] "P value for elegans vs. dominicensis = 0.234480173997123"
```

Figure A.44

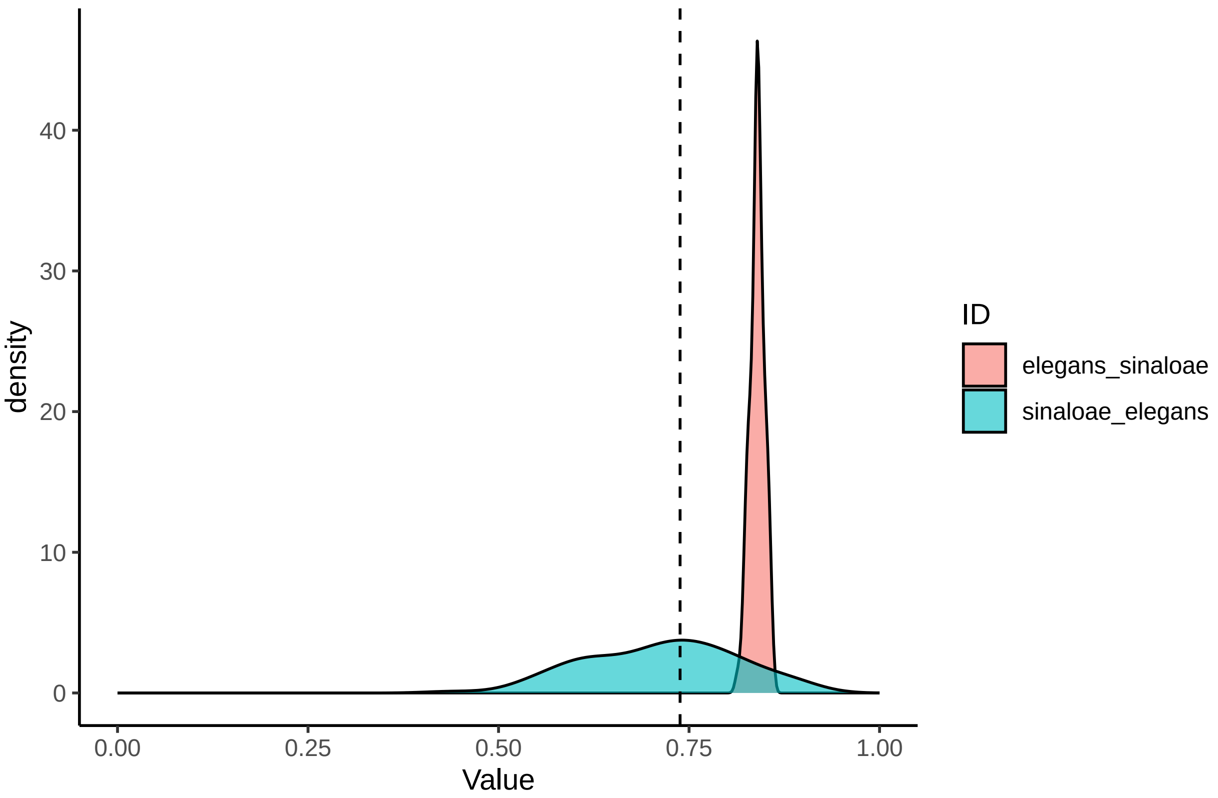


```
## [1] "elegans_sinaloae"
## [1] "P value for elegans vs. sinaloae = 1.7424162547499e-27"
## [1] "Test statistic below low critical value."
## [1] "0.821080281493838; statistic = 0.738253600383395"
## [1] "sinaloae_elegans"
## [1] "P value for elegans vs. sinaloae = 0.597446716031685"
```

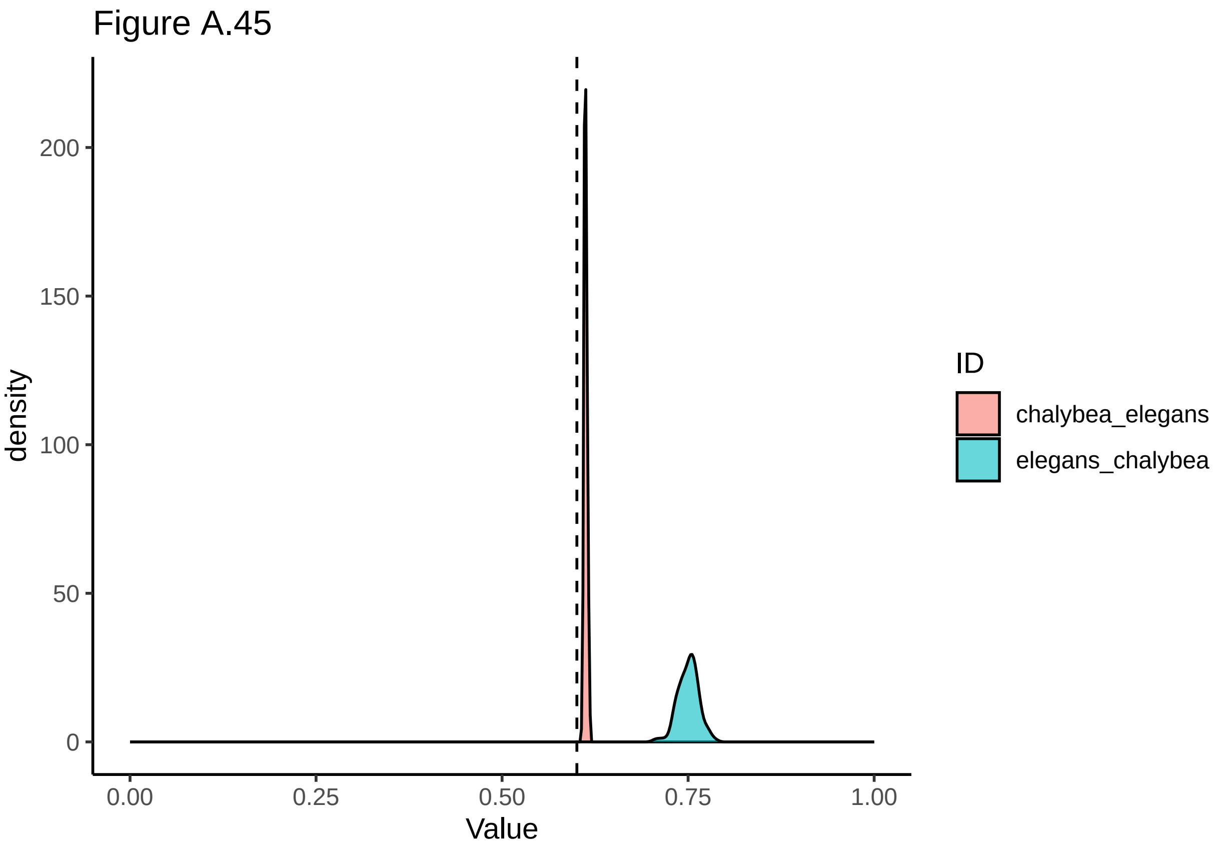


```
## [1] "elegans_chalybea"
## [1] "P value for elegans vs. chalybea = 3.06799382017121e-28"
## [1] "Test statistic below low critical value."
## [1] "0.723481951512536; statistic = 0.600536008828745"
## [1] "chalybea_elegans"
## [1] "P value for elegans vs. chalybea = 1.1268673669308e-10"
## [1] "Test statistic below low critical value."
## [1] "0.608673933067226; statistic = 0.600536008828745"
```

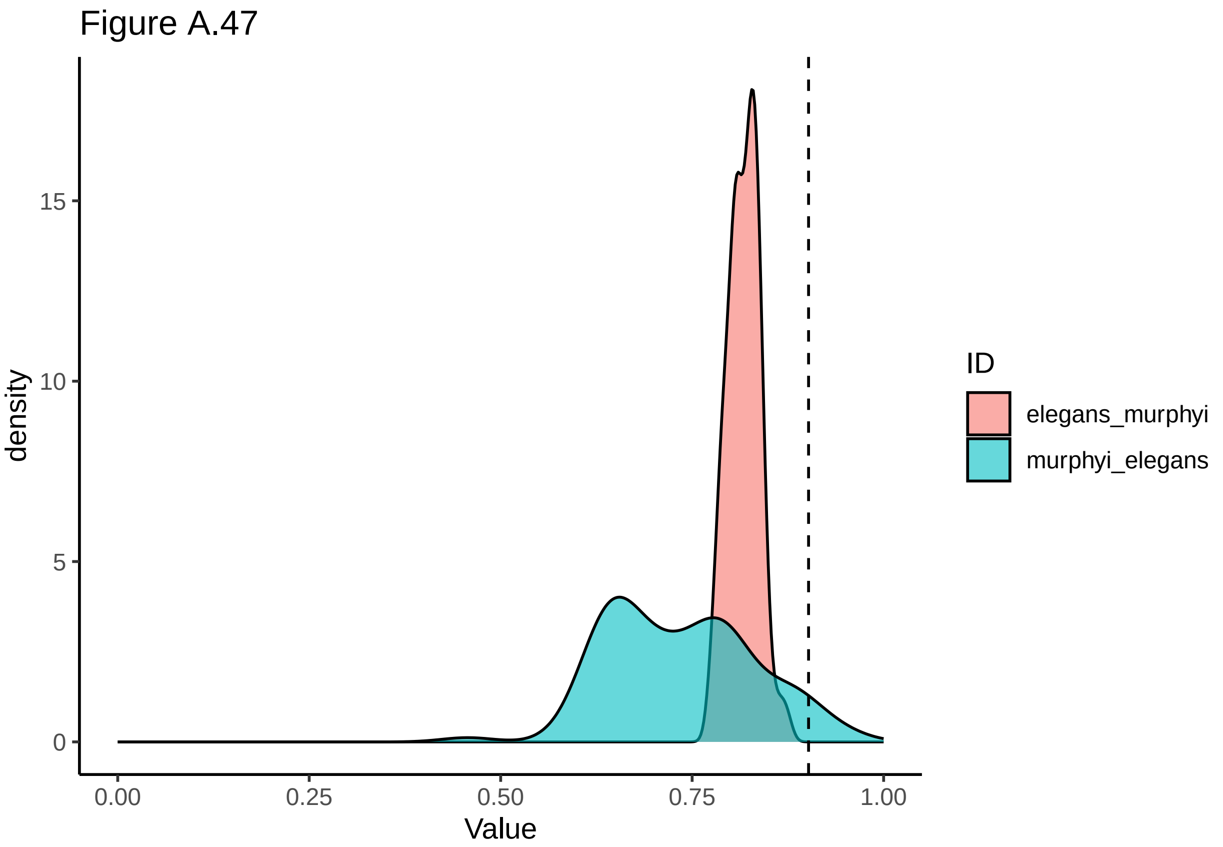


```
## [1] "elegans_murphyi"
## [1] "P value for elegans vs. murphyi = 0.999987304433576"
## [1] "Test statistic above high critical value."
## [1] "0.85663781619806; statistic = 0.901990509419277"
## [1] "murphyi_elegans"
## [1] "P value for elegans vs. murphyi = 0.961251333736766"
```

Figure A.48

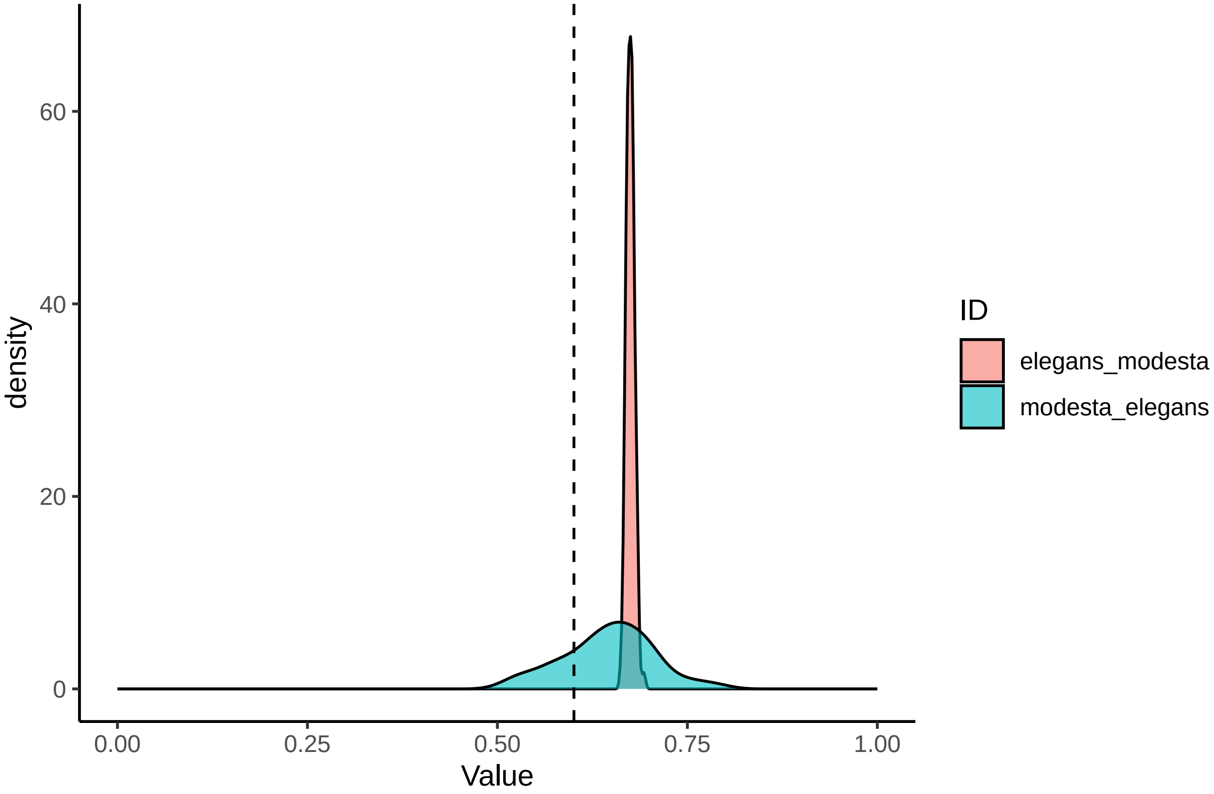


```
## [1] "elegans_modesta"
## [1] "P value for elegans vs. modesta = 6.59915408259048e-47"
## [1] "Test statistic below low critical value."
## [1] "0.66486893233368; statistic = 0.600700504205202"
## [1] "modesta_elegans"
## [1] "P value for elegans vs. modesta = 0.22031065896722"
```

Figure A.49

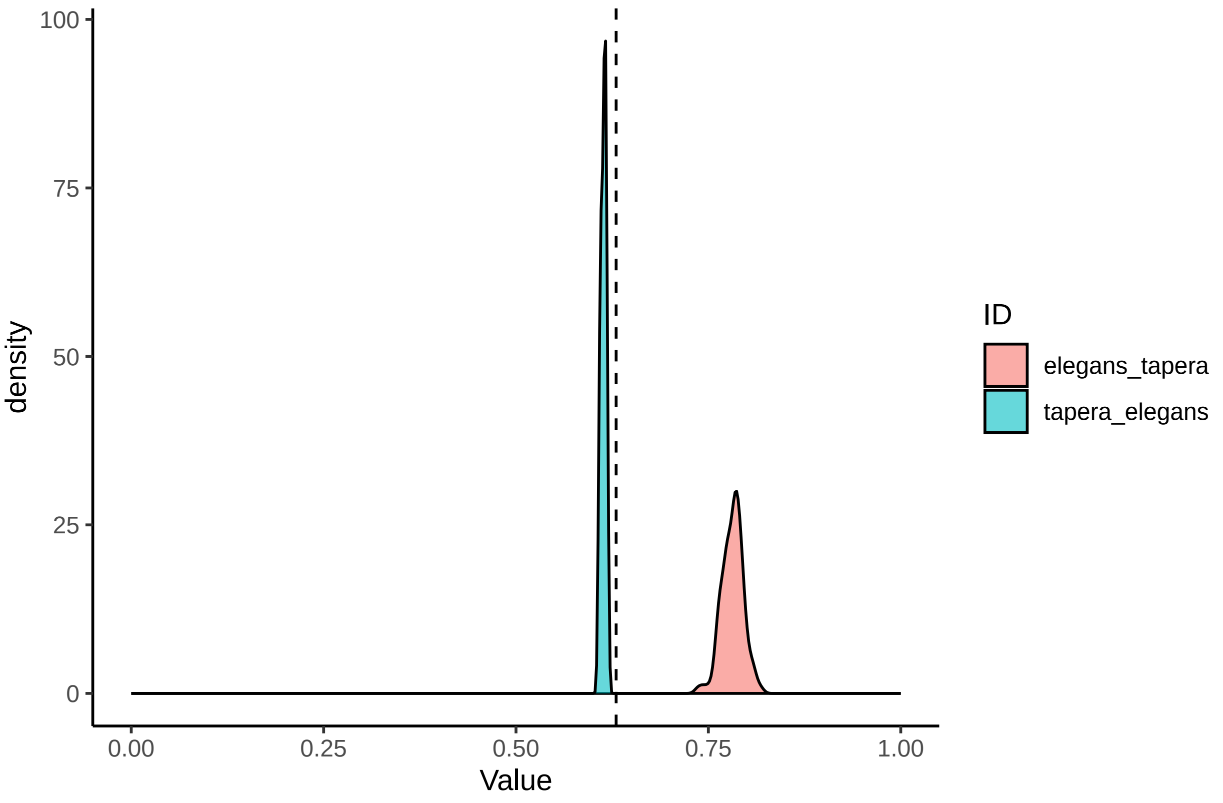


```
## [1] "elegans_tapera"
## [1] "P value for elegans vs. tapera = 2.28619337933981e-28"
## [1] "Test statistic below low critical value."
## [1] "0.754411253629397; statistic = 0.63014437319718"
## [1] "tapera_elegans"
## [1] "P value for elegans vs. tapera = 0.999999492809445"
## [1] "Test statistic above high critical value."
## [1] "0.620340813315704; statistic = 0.63014437319718"
```

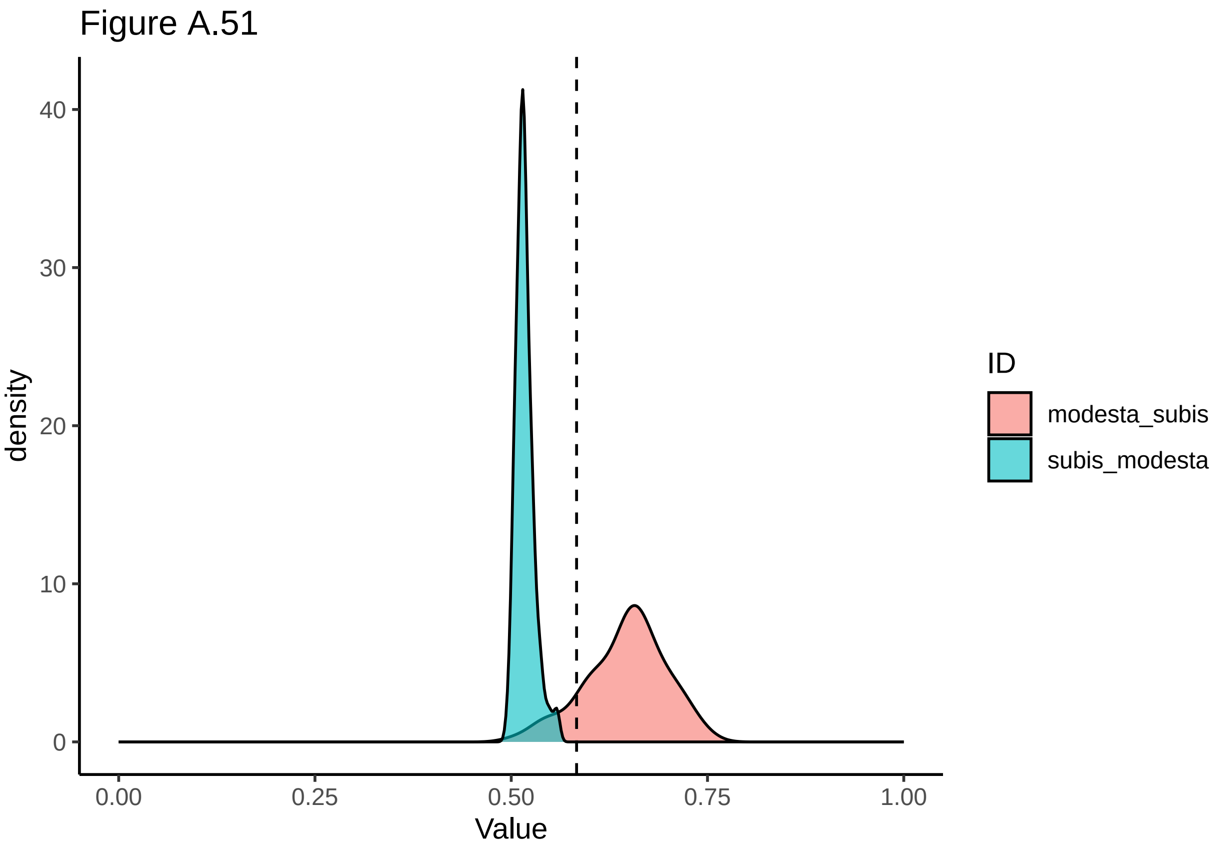


```
## [1] "modesta_subis"
## [1] "P value for modesta vs. subis = 0.1078831536929"
## [1] "subis_modesta"
## [1] "P value for modesta vs. subis = 0.999999988800046"
## [1] "Test statistic above high critical value."
## [1] "0.540528555485547; statistic = 0.583264877616411"
```

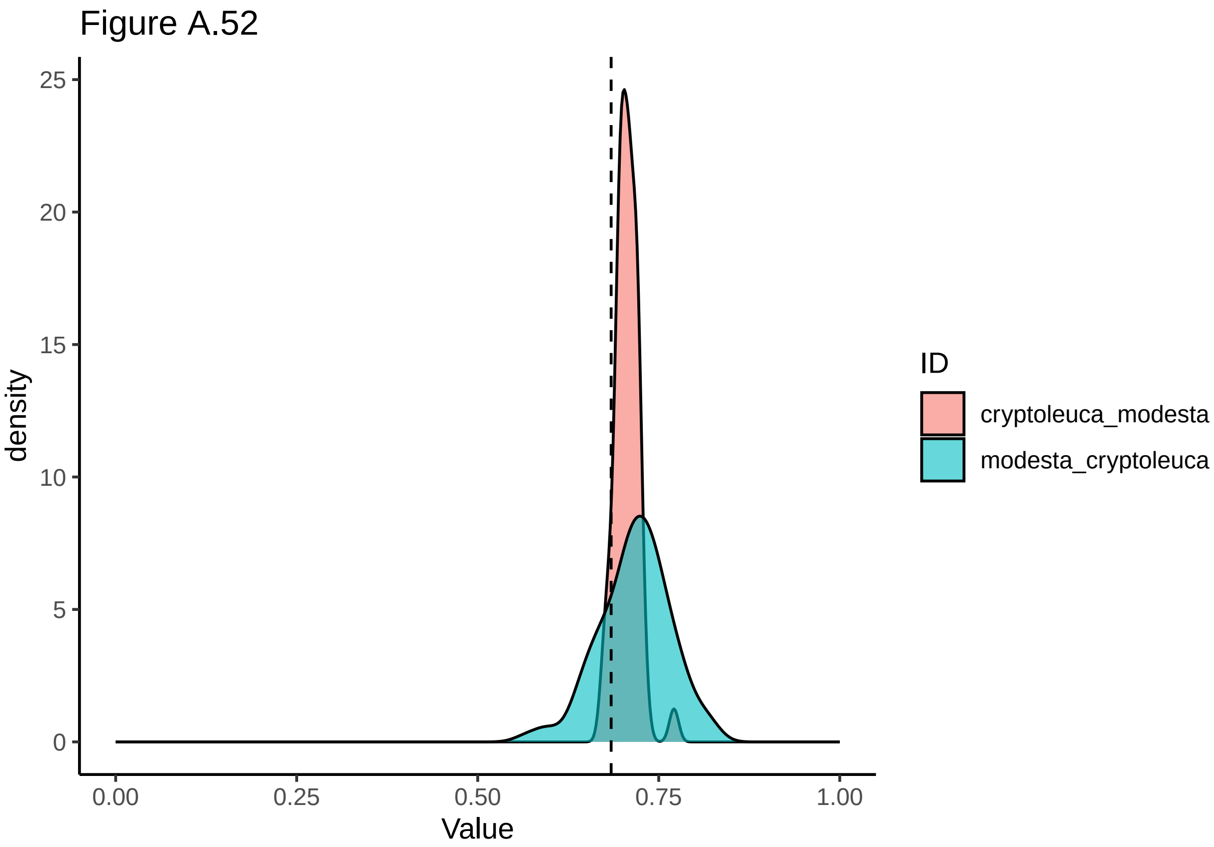


```
## [1] "modesta_cryptoleuca"
## [1] "P value for modesta vs. cryptoleuca = 0.251702770103792"
## [1] "cryptoleuca_modesta"
## [1] "P value for modesta vs. cryptoleuca = 0.092096150558271"
```

Figure A.53

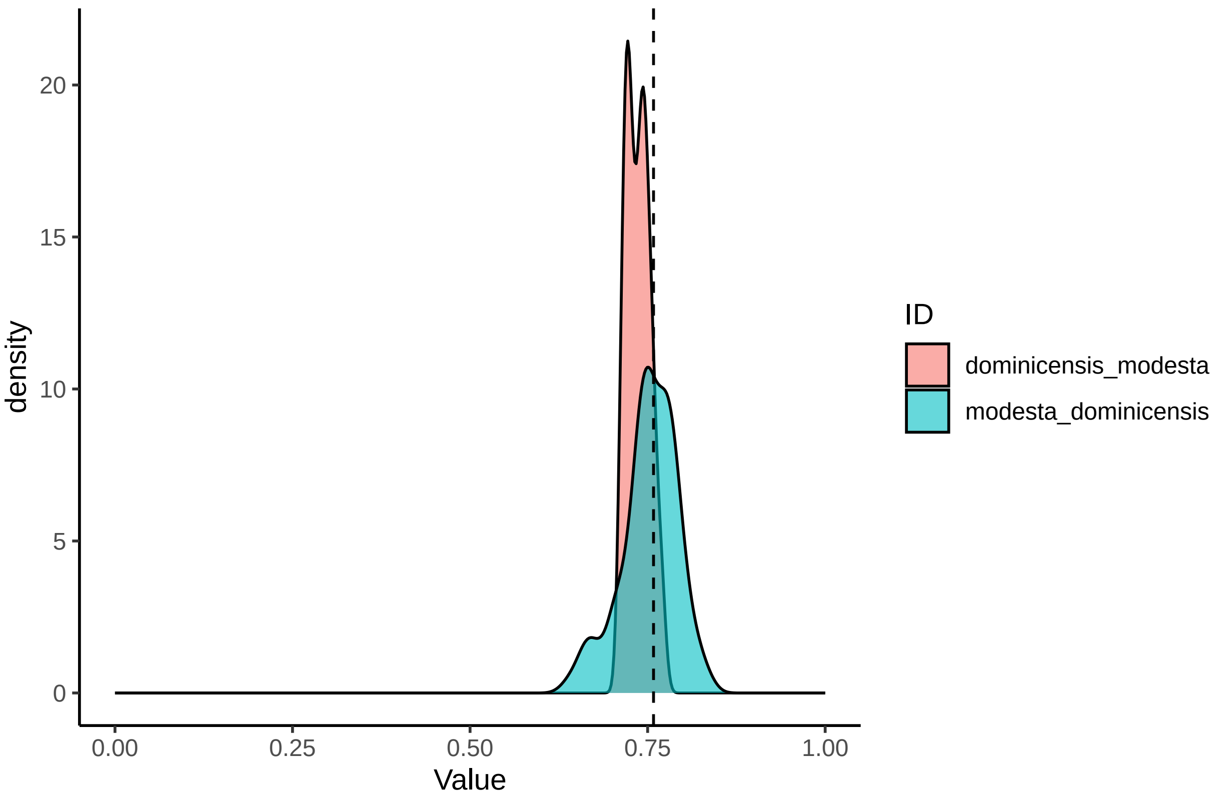


```
## [1] "modesta_dominicensis"
## [1] "P value for modesta vs. dominicensis = 0.560153596534548"
## [1] "dominicensis_modesta"
## [1] "P value for modesta vs. dominicensis = 0.912132791877994"
```

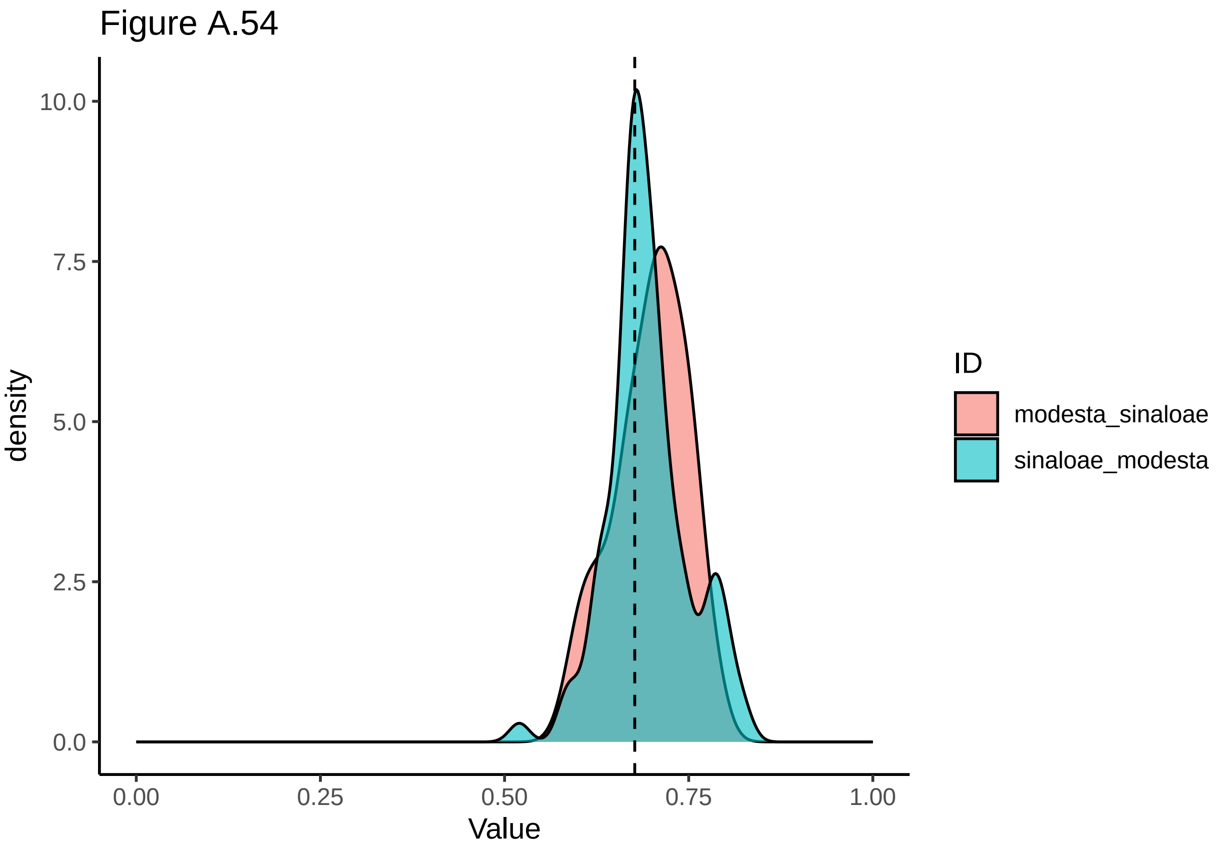


```
## [1] "modesta_sinaloae"
## [1] "P value for modesta vs. sinaloae = 0.333801326243559"
## [1] "sinaloae_modesta"
## [1] "P value for modesta vs. sinaloae = 0.374690294593921"
```

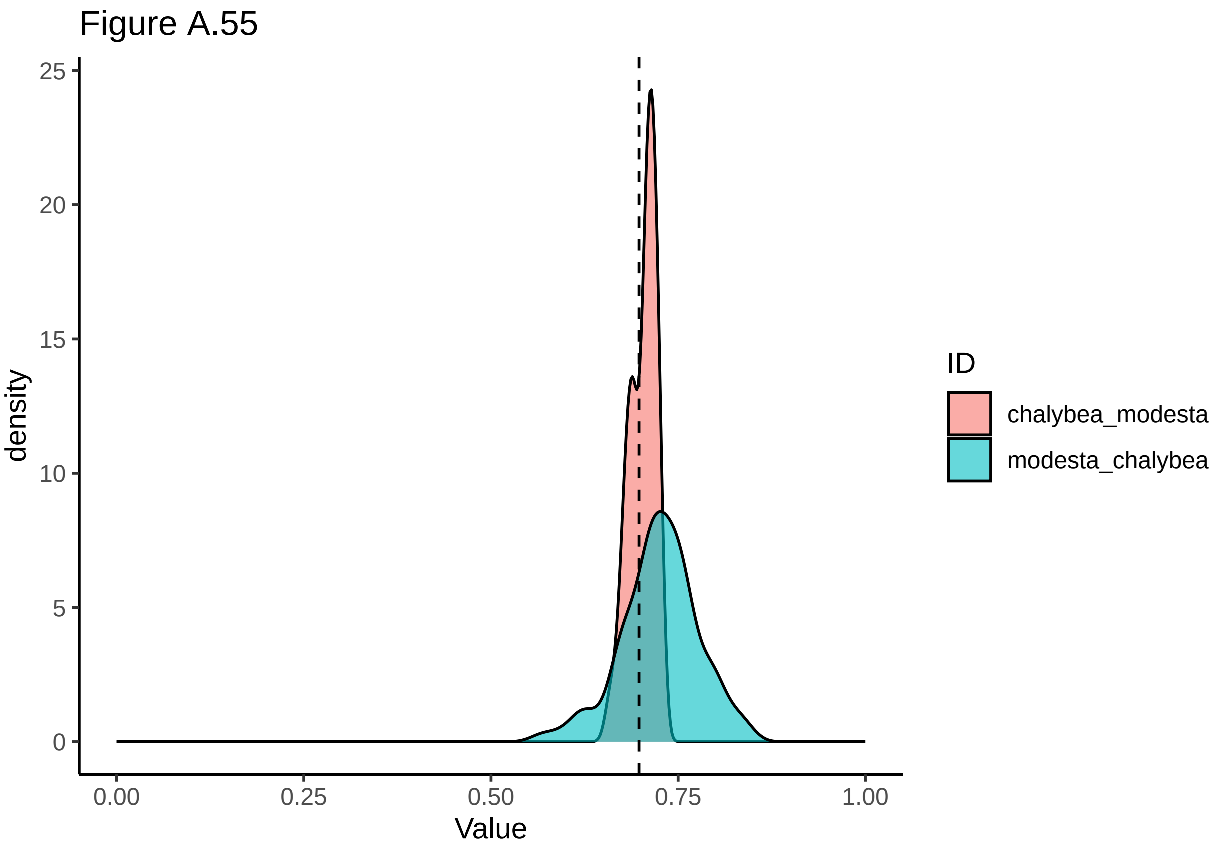


```
## [1] "modesta_chalybea"
## [1] "P value for modesta vs. chalybea = 0.294789673096891"
## [1] "chalybea_modesta"
## [1] "P value for modesta vs. chalybea = 0.389634581657429"
```

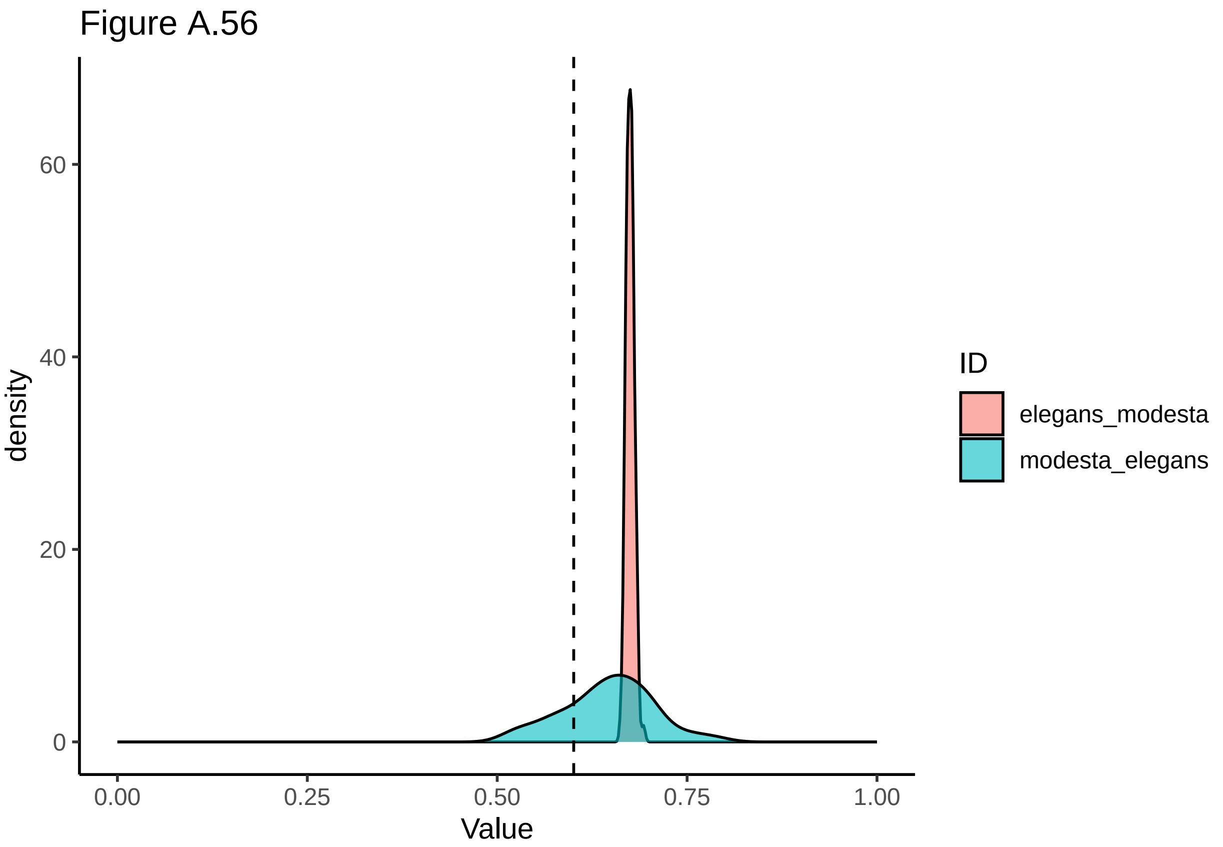


```
## [1] "modesta_elegans"
## [1] "P value for modesta vs. elegans = 0.220310658967219"
## [1] "elegans_modesta"
## [1] "P value for modesta vs. elegans = 6.59915408258642e-47"
## [1] "Test statistic below low critical value."
## [1] "0.66486893233368; statistic = 0.600700504205202"
```

Figure A.57

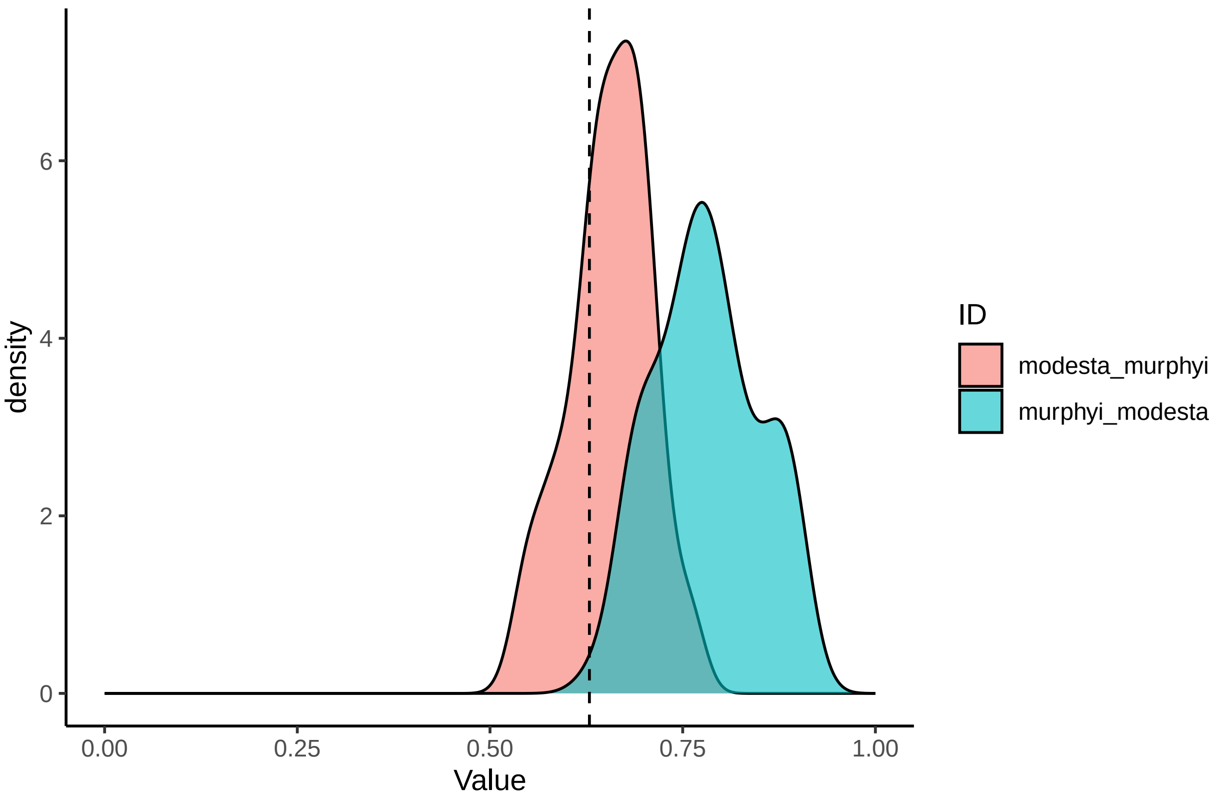


```
## [1] "modesta_murphyi"
## [1] "P value for modesta vs. murphyi = 0.306293889036427"
## [1] "murphyi_modesta"
## [1] "P value for modesta vs. murphyi = 0.0125321881521672"
## [1] "Test statistic below low critical value."
## [1] "0.648027960567164; statistic = 0.62897231131383"
```

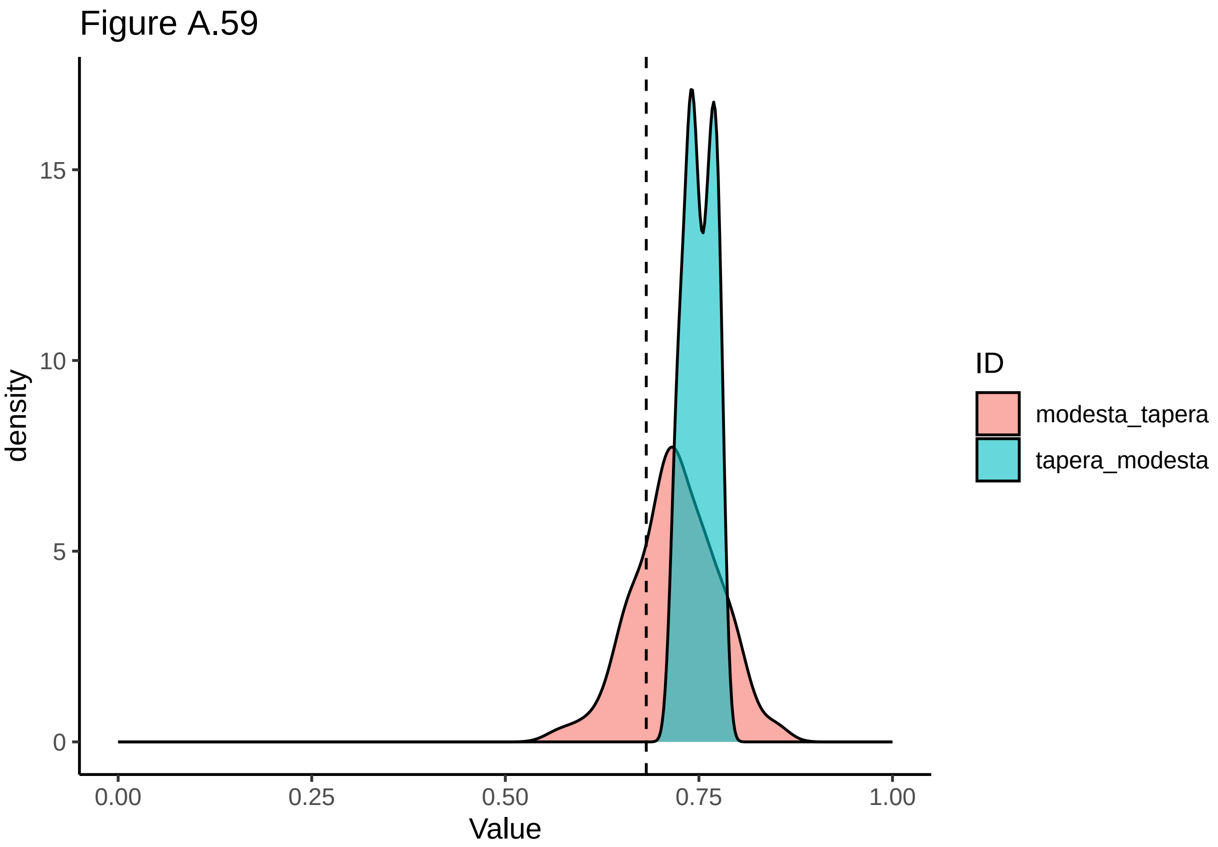


```
## [1] "modesta_tapera"
## [1] "P value for modesta vs. tapera = 0.233956062658453"
## [1] "tapera_modesta"
## [1] "P value for modesta vs. tapera = 0.000167011894132902"
## [1] "Test statistic below low critical value."
## [1] "0.712997150805312; statistic = 0.68199189109927"
```

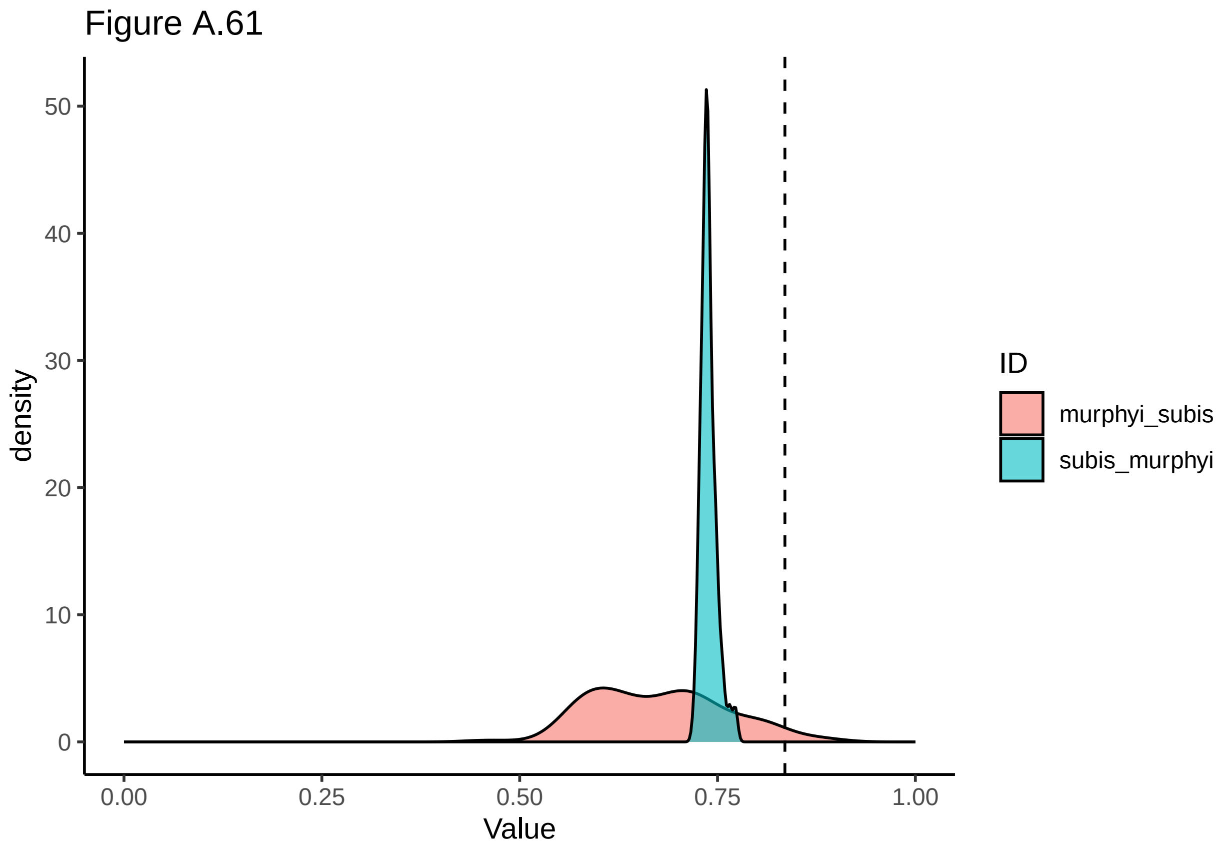


```
## [1] "murphyi_subis"
## [1] "P value for murphyi vs. subis = 0.971139295288171"
## [1] "subis_murphyi"
## [1] "P value for murphyi vs. subis = 1"
## [1] "Test statistic above high critical value."
## [1] "0.758298132126779; statistic = 0.835123293713782"
```

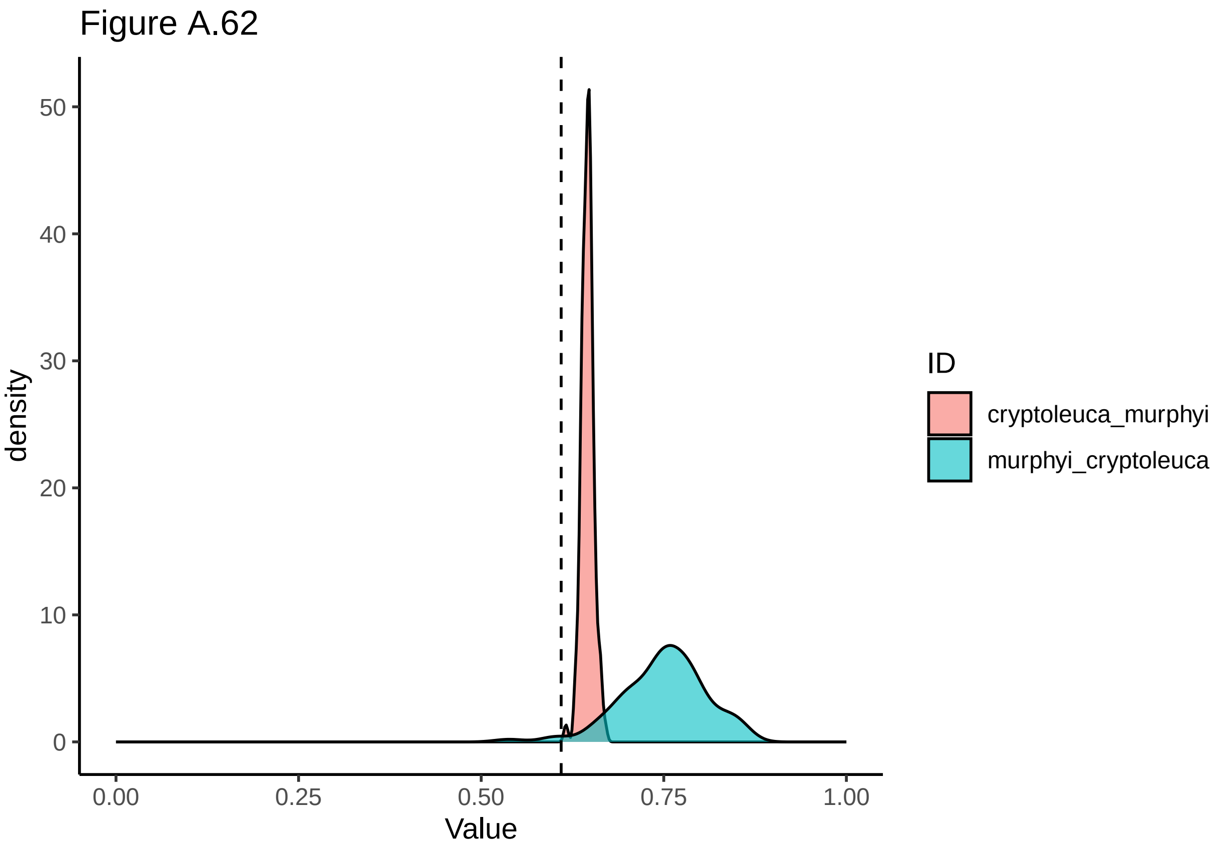


```
## [1] "murphyi_cryptoleuca"
## [1] "P value for murphyi vs. cryptoleuca = 0.00765410075705068"
## [1] "Test statistic below low critical value."
## [1] "0.636418105864818; statistic = 0.609509779095934"
## [1] "cryptoleuca_murphyi"
## [1] "P value for murphyi vs. cryptoleuca = 6.61526226502393e-06"
## [1] "Test statistic below low critical value."
## [1] "0.629372014246327; statistic = 0.609509779095934"
```

Figure A.63

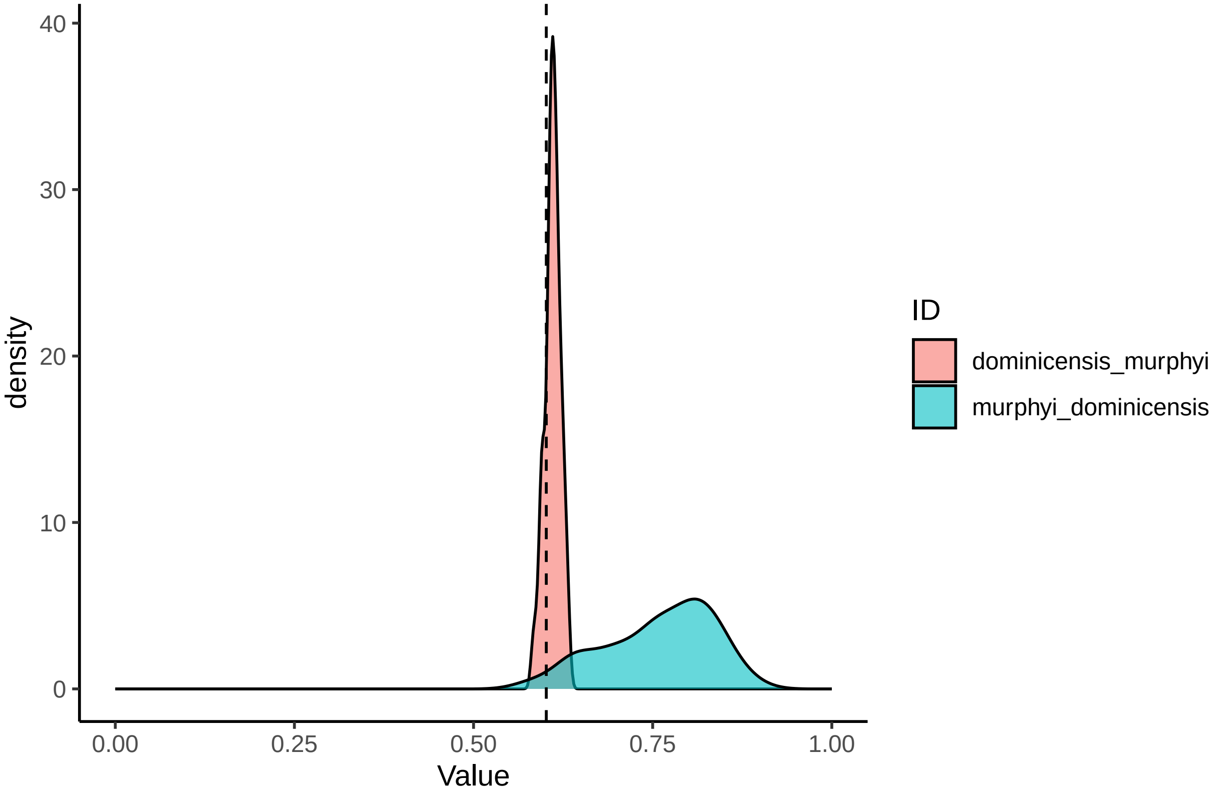


```
## [1] "murphyi_dominicensis"
## [1] "P value for murphyi vs. dominicensis = 0.017876444789363"
## [1] "Test statistic below low critical value."
## [1] "0.61194638994795; statistic = 0.601502602342795"
## [1] "dominicensis_murphyi"
## [1] "P value for murphyi vs. dominicensis = 0.204464259340592"
```

Figure A.64

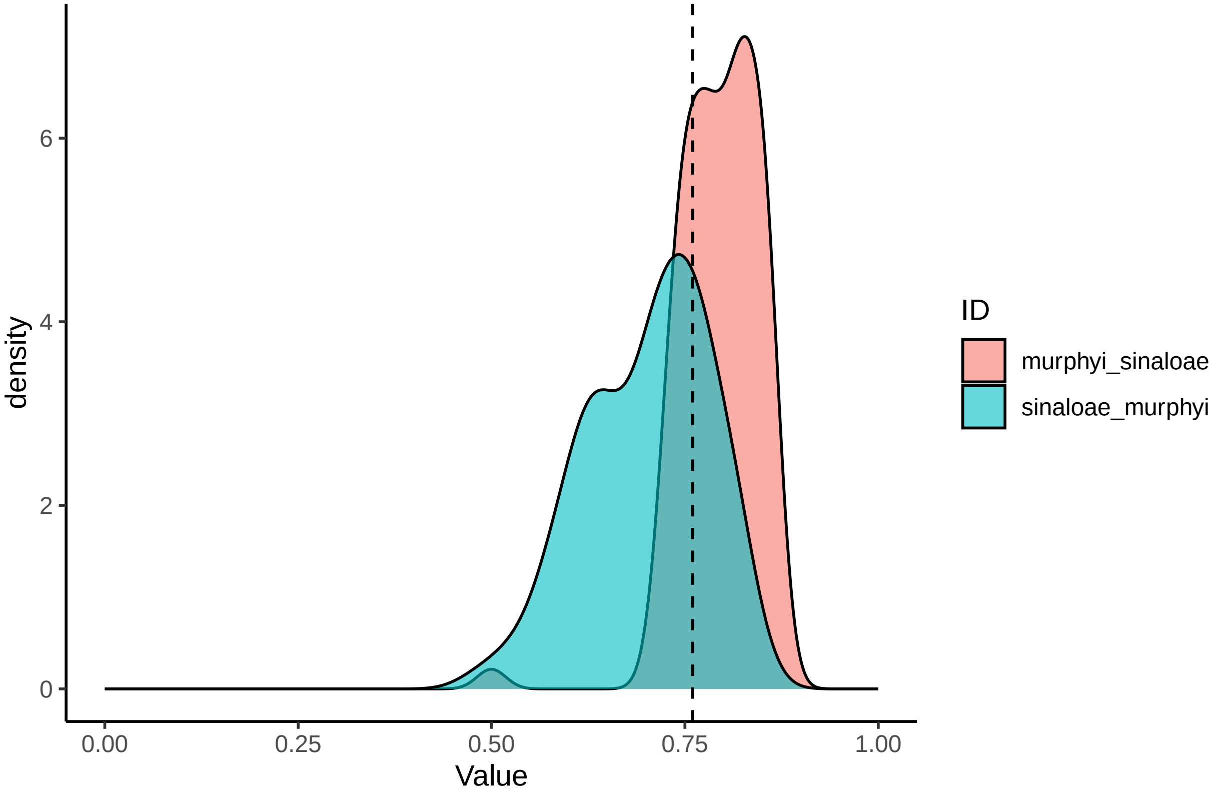


```
## [1] "murphyi_sinaloae"
## [1] "P value for murphyi vs. sinaloae = 0.249887811838028"
## [1] "sinaloae_murphyi"
## [1] "P value for murphyi vs. sinaloae = 0.76773496956385"
```

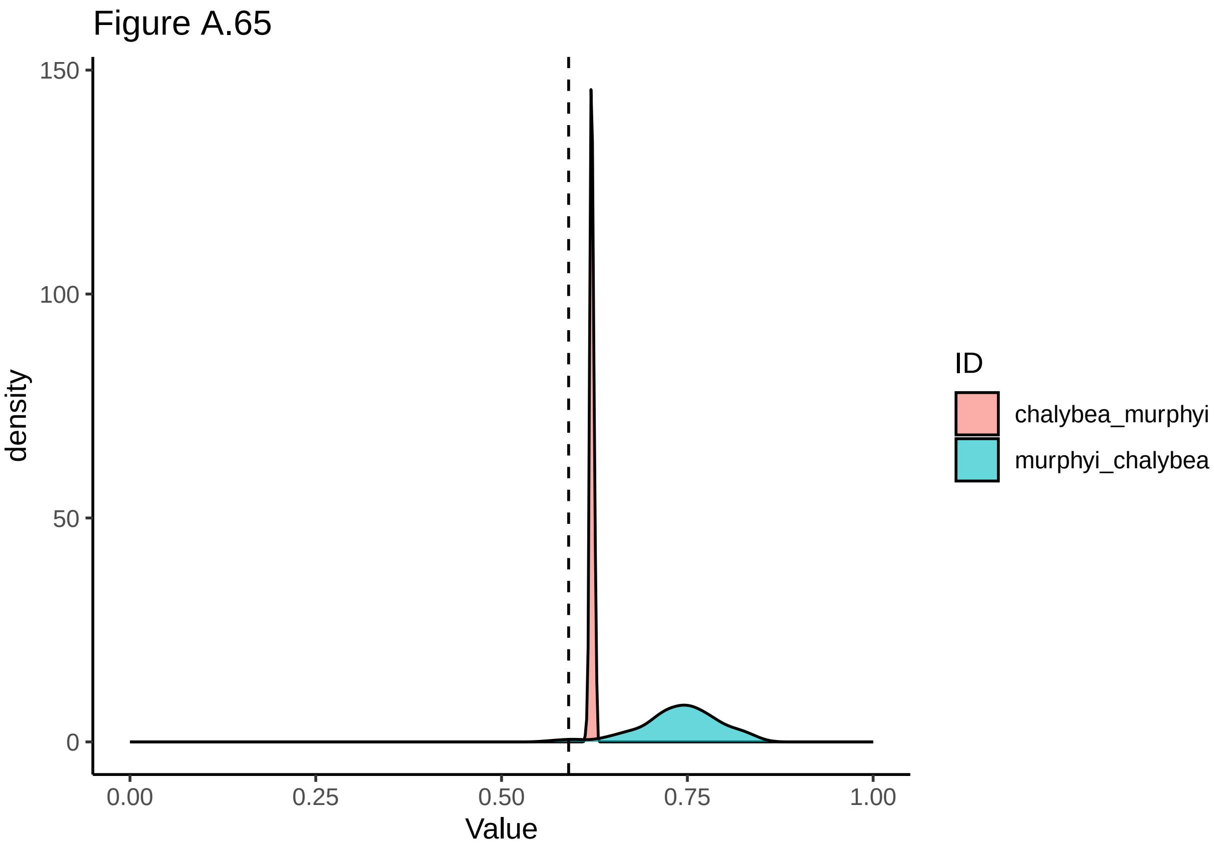


```
## [1] "murphyi_chalybea"
## [1] "P value for murphyi vs. chalybea = 0.00216732497679625"
## [1] "Test statistic below low critical value."
## [1] "0.637154158297721; statistic = 0.590267738596306"
## [1] "chalybea_murphyi"
## [1] "P value for murphyi vs. chalybea = 1.30125236540328e-37"
## [1] "Test statistic below low critical value."
## [1] "0.616780352612352; statistic = 0.590267738596306"
```

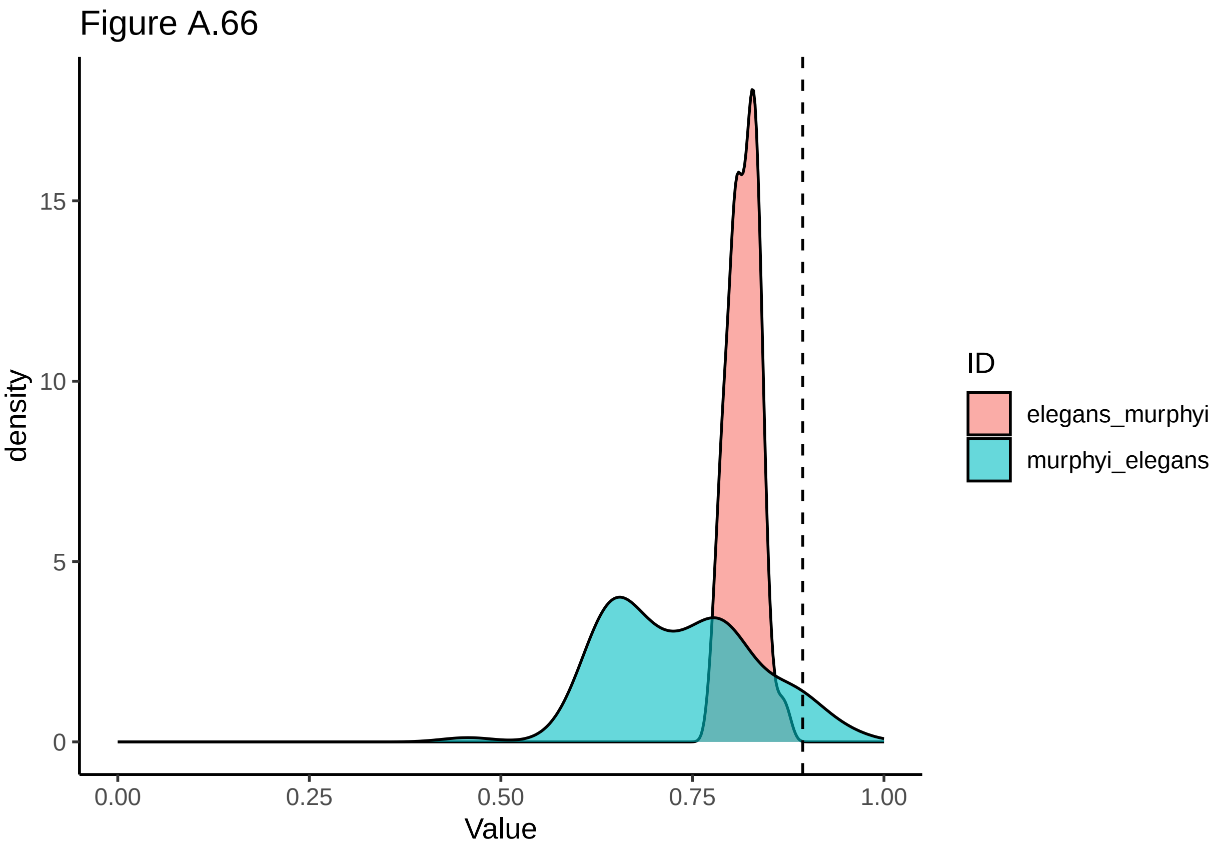


```
## [1] "murphyi_elegans"
## [1] "P value for murphyi vs. elegans = 0.953606783256938"
## [1] "elegans_murphyi"
## [1] "P value for murphyi vs. elegans = 0.999931943900692"
## [1] "Test statistic above high critical value."
## [1] "0.85663781619806; statistic = 0.894009802454622"
```

Figure A.68

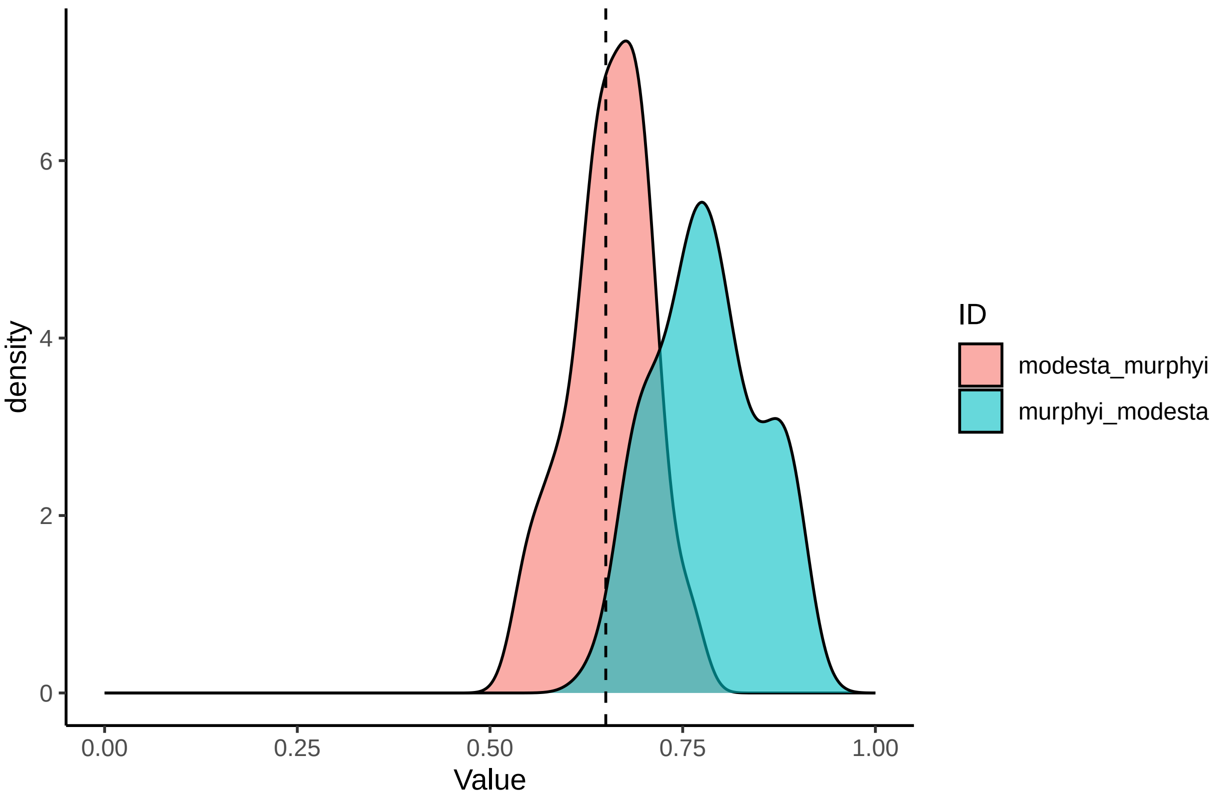


```
## [1] "murphyi_modesta"
## [1] "P value for murphyi vs. modesta = 0.0269308162178768"
## [1] "modesta_murphyi"
## [1] "P value for murphyi vs. modesta = 0.459275587048333"
```

Figure A.69

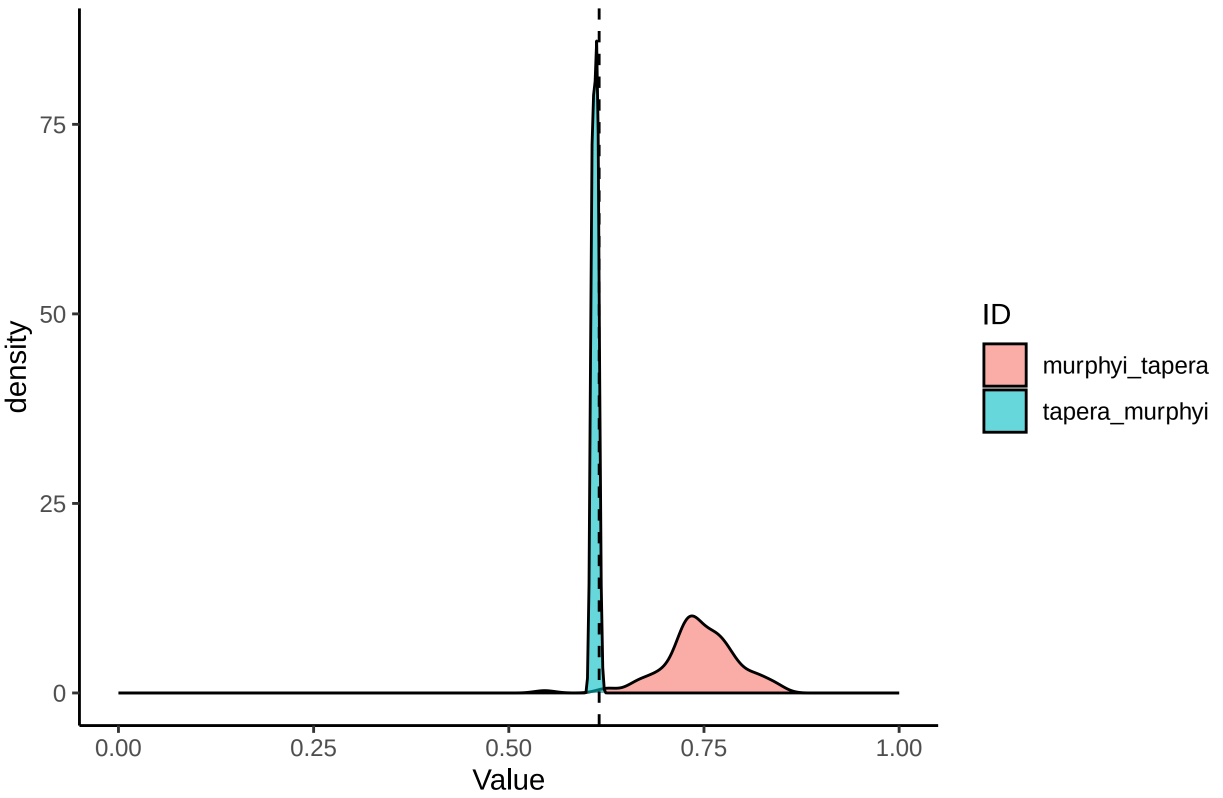


```
## [1] "murphyi_tapera"
## [1] "P value for murphyi vs. tapera = 0.00388454823008584"
## [1] "Test statistic below low critical value."
## [1] "0.649649760588276; statistic = 0.615724242493323"
## [1] "tapera_murphyi"
## [1] "P value for murphyi vs. tapera = 0.923749328771478"
```

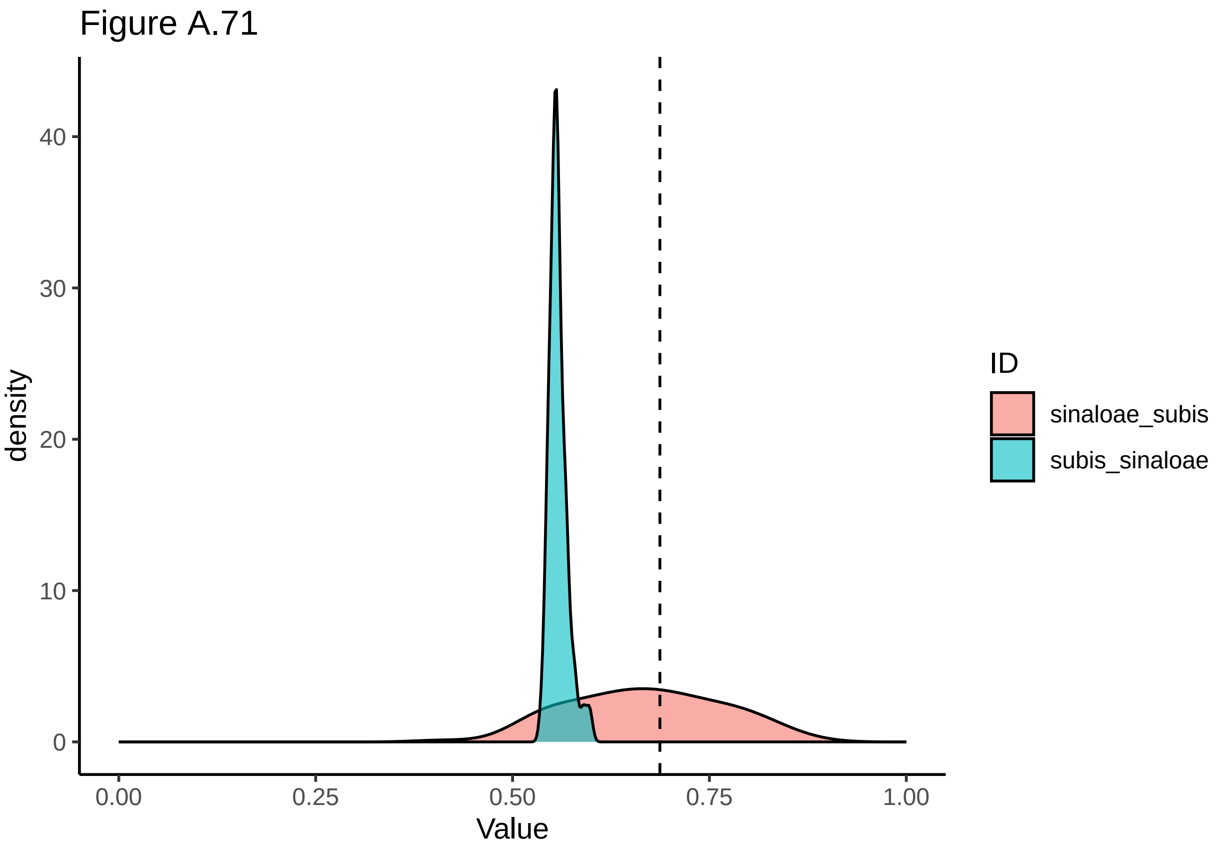


```
## [1] "sinaloae_subis"
## [1] "P value for sinaloae vs. subis = 0.573177191904059"
## [1] "subis_sinaloae"
## [1] "P value for sinaloae vs. subis = 1"
## [1] "Test statistic above high critical value."
## [1] "0.580979920109348; statistic = 0.687111531570226"
```

Figure A.72

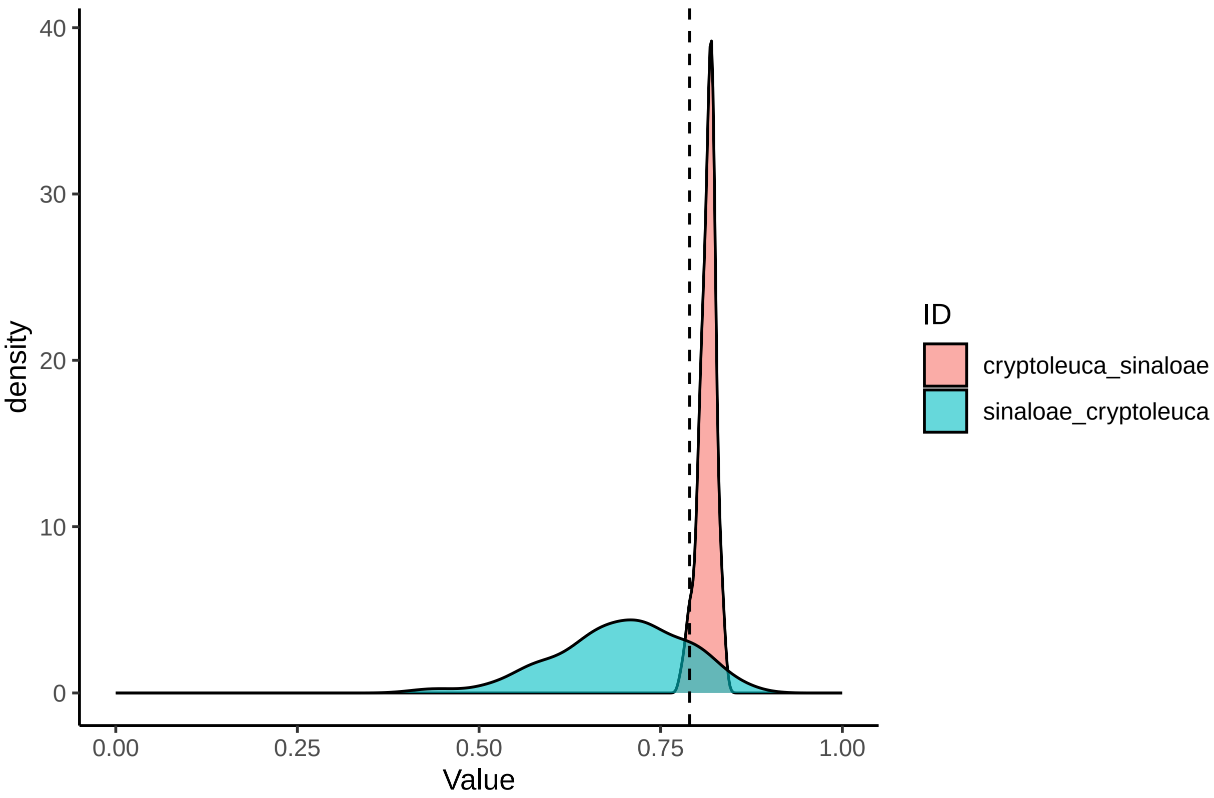


```
## [1] "sinaloae_cryptoleuca"
## [1] "P value for sinaloae vs. cryptoleuca = 0.858123249895697"
## [1] "cryptoleuca_sinaloae"
## [1] "P value for sinaloae vs. cryptoleuca = 0.0178629901775483"
## [1] "Test statistic below low critical value."
## [1] "0.791481323974456; statistic = 0.789848394763692"
```

Figure A.73

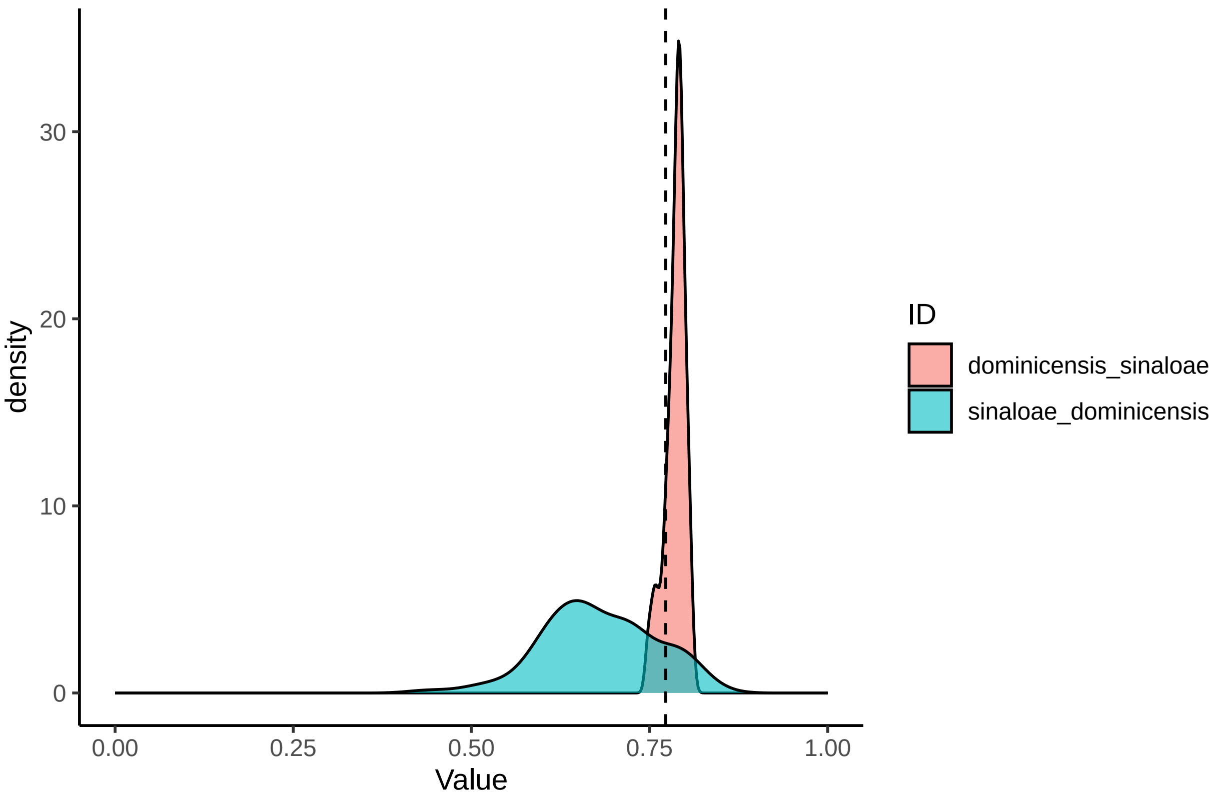


```
## [1] "sinaloae_dominicensis"
## [1] "P value for sinaloae vs. dominicensis = 0.883512971775754"
## [1] "dominicensis_sinaloae"
## [1] "P value for sinaloae vs. dominicensis = 0.168545748778204"
```

Figure A.75

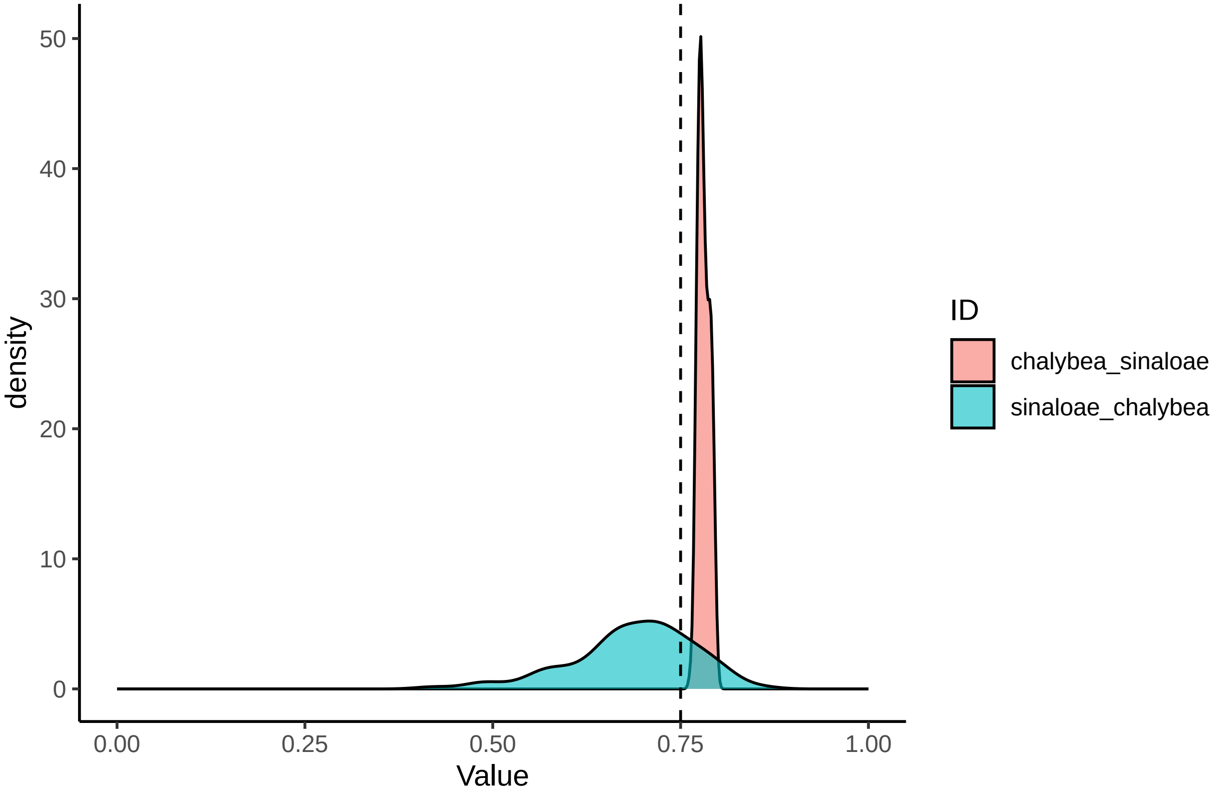


```
## [1] "sinaloae_chalybea"
## [1] "P value for sinaloae vs. chalybea = 0.77871818448255"
## [1] "chalybea_sinaloae"
## [1] "P value for sinaloae vs. chalybea = 2.58592319317009e-05"
## [1] "Test statistic below low critical value."
## [1] "0.765954690487922; statistic = 0.750023122900644"
```

Figure A.76

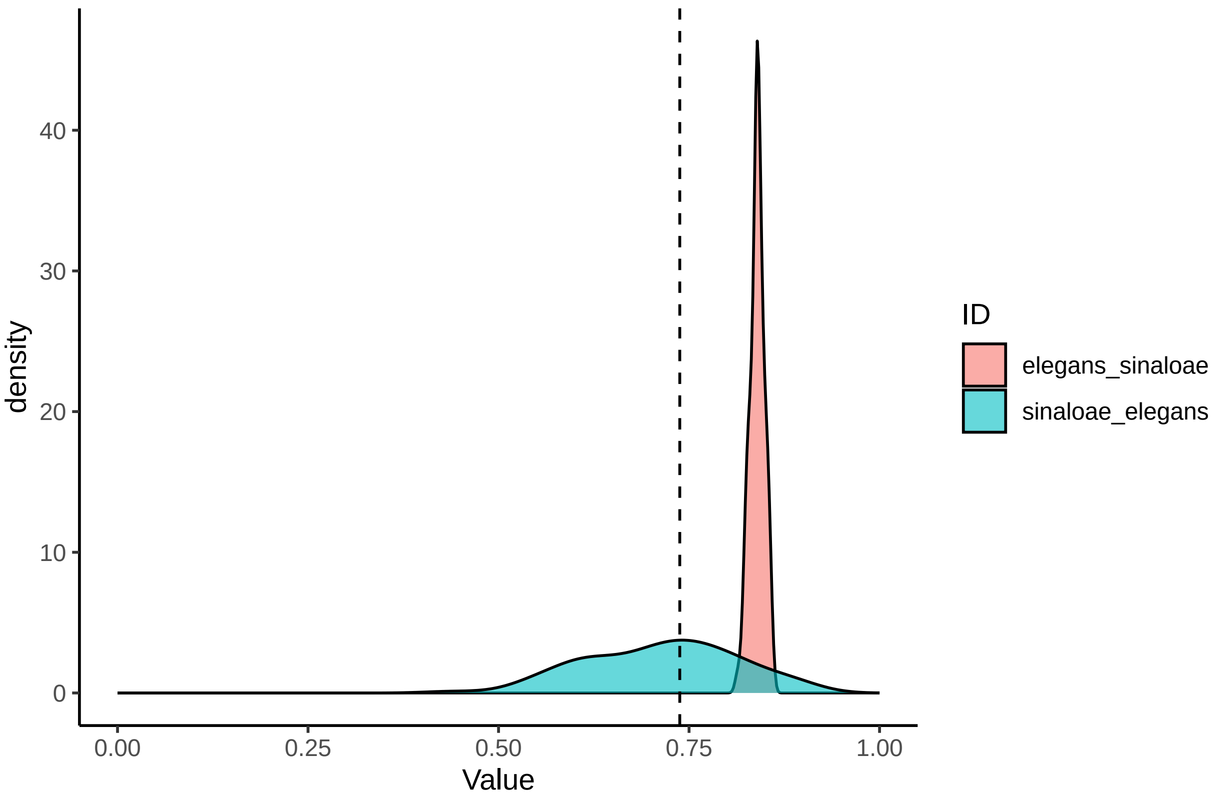


```
## [1] "sinaloae_elegans"
## [1] "P value for sinaloae vs. elegans = 0.595819427529082"
## [1] "elegans_sinaloae"
## [1] "P value for sinaloae vs. elegans = 1.07682315635689e-27"
## [1] "Test statistic below low critical value."
## [1] "0.821080281493838; statistic = 0.737840312619779"
```

Figure A.77

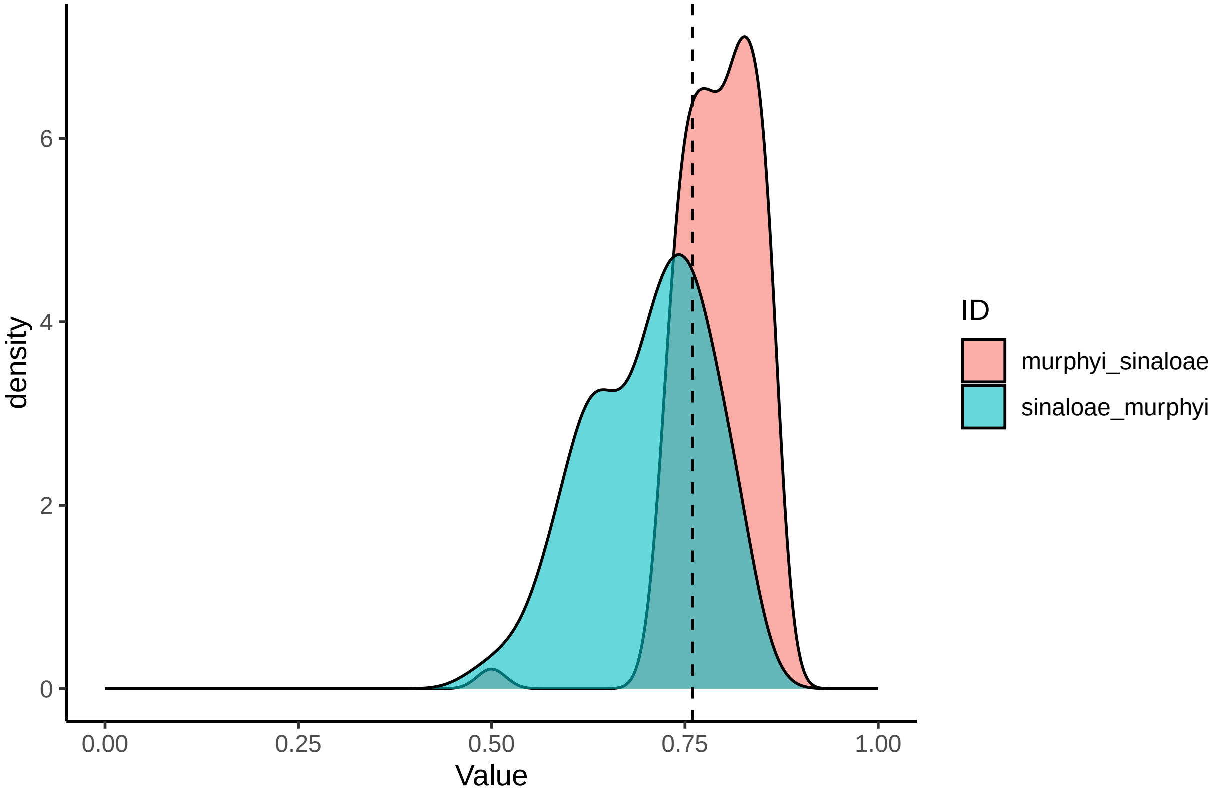


```
## [1] "sinaloae_murphyi"
## [1] "P value for sinaloae vs. murphyi = 0.76773496956385"
## [1] "murphyi_sinaloae"
## [1] "P value for sinaloae vs. murphyi = 0.249887811838028"
```

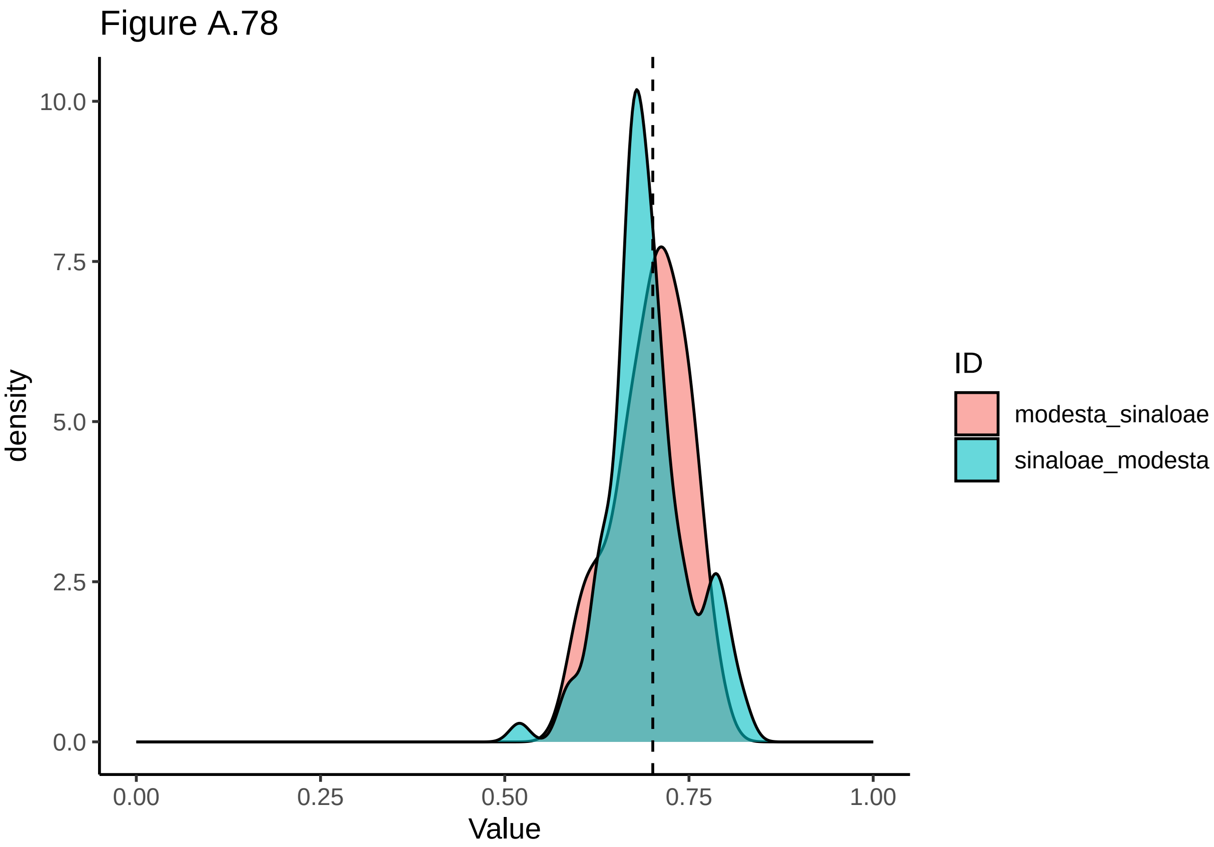


```
## [1] "sinaloae_modesta"
## [1] "P value for sinaloae vs. modesta = 0.549559407515952"
## [1] "modesta_sinaloae"
## [1] "P value for sinaloae vs. modesta = 0.522509267513247"
```

Figure A.79

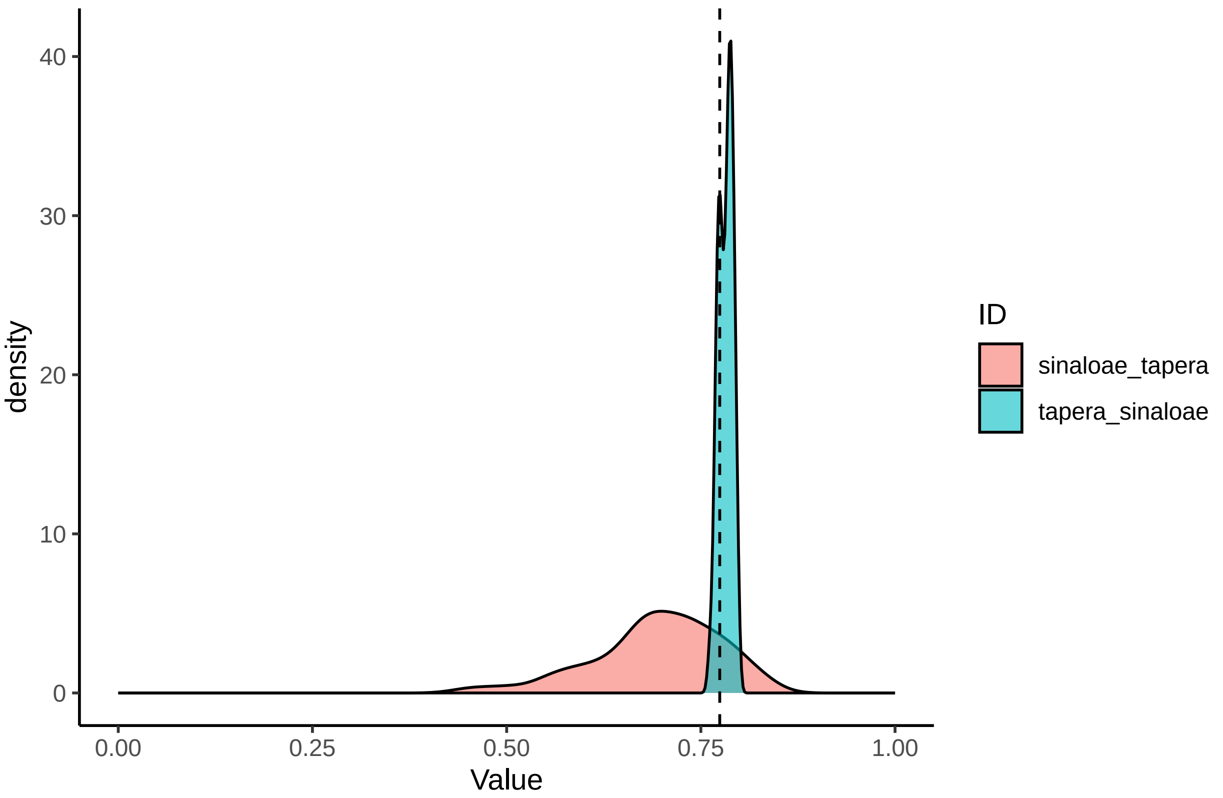


```
## [1] "sinaloae_tapera"
## [1] "P value for sinaloae vs. tapera = 0.837385351802245"
## [1] "tapera_sinaloae"
## [1] "P value for sinaloae vs. tapera = 0.188657928500043"
```

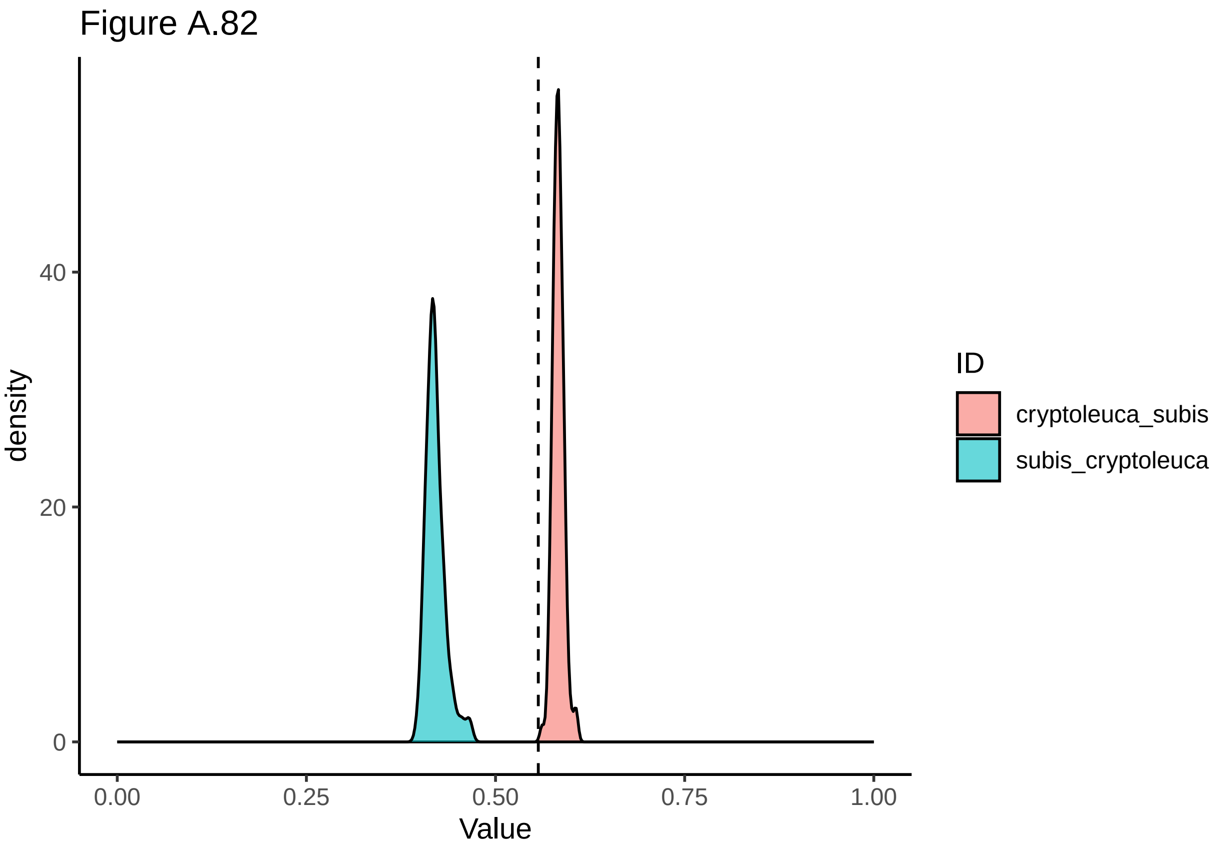


```
## [1] "subis_cryptoleuca"
## [1] "P value for subis vs. cryptoleuca = 1"
## [1] "Test statistic above high critical value."
## [1] "0.446182291694113; statistic = 0.556518426277204"
## [1] "cryptoleuca_subis"
## [1] "P value for subis vs. cryptoleuca = 0.000171916898040372"
## [1] "Test statistic below low critical value."
## [1] "0.568482313562568; statistic = 0.556518426277204"
```

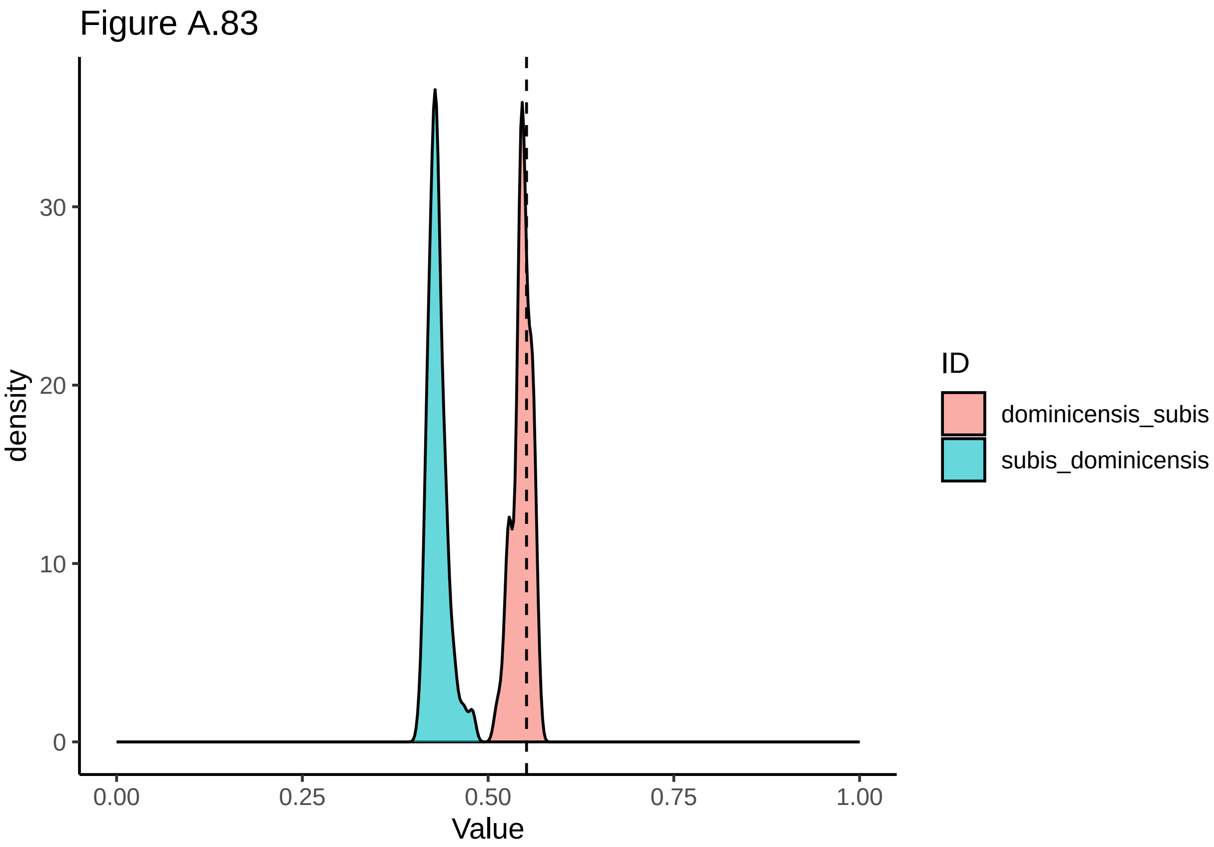


```
## [1] "subis_dominicensis"
## [1] "P value for subis vs. dominicensis = 1"
## [1] "Test statistic above high critical value."
## [1] "0.458350885803299; statistic = 0.551734842020407"
## [1] "dominicensis_subis"
## [1] "P value for subis vs. dominicensis = 0.687438381466892"
```

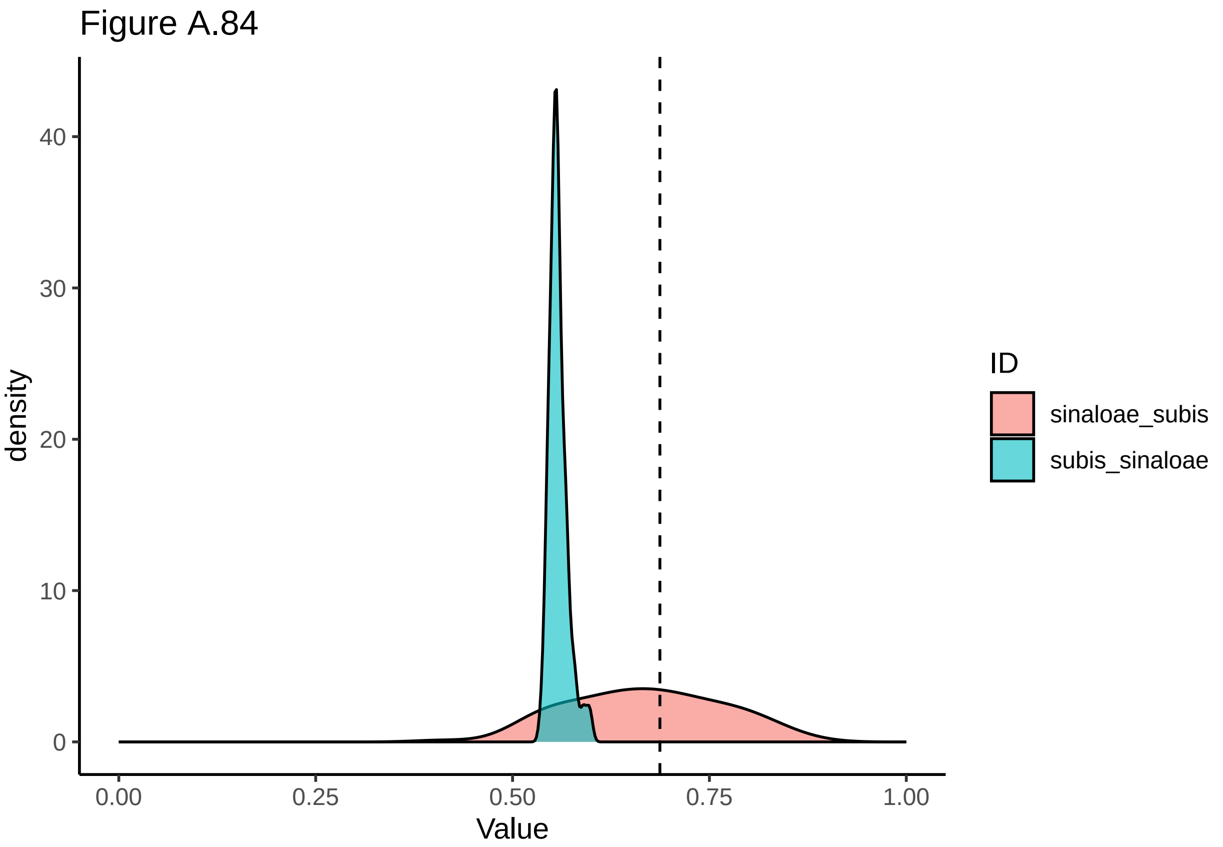


```
## [1] "subis_sinaloae"
## [1] "P value for subis vs. sinaloae = 1"
## [1] "Test statistic above high critical value."
## [1] "0.580979920109348; statistic = 0.687111531570226"
## [1] "sinaloae_subis"
## [1] "P value for subis vs. sinaloae = 0.573177191904059"
```

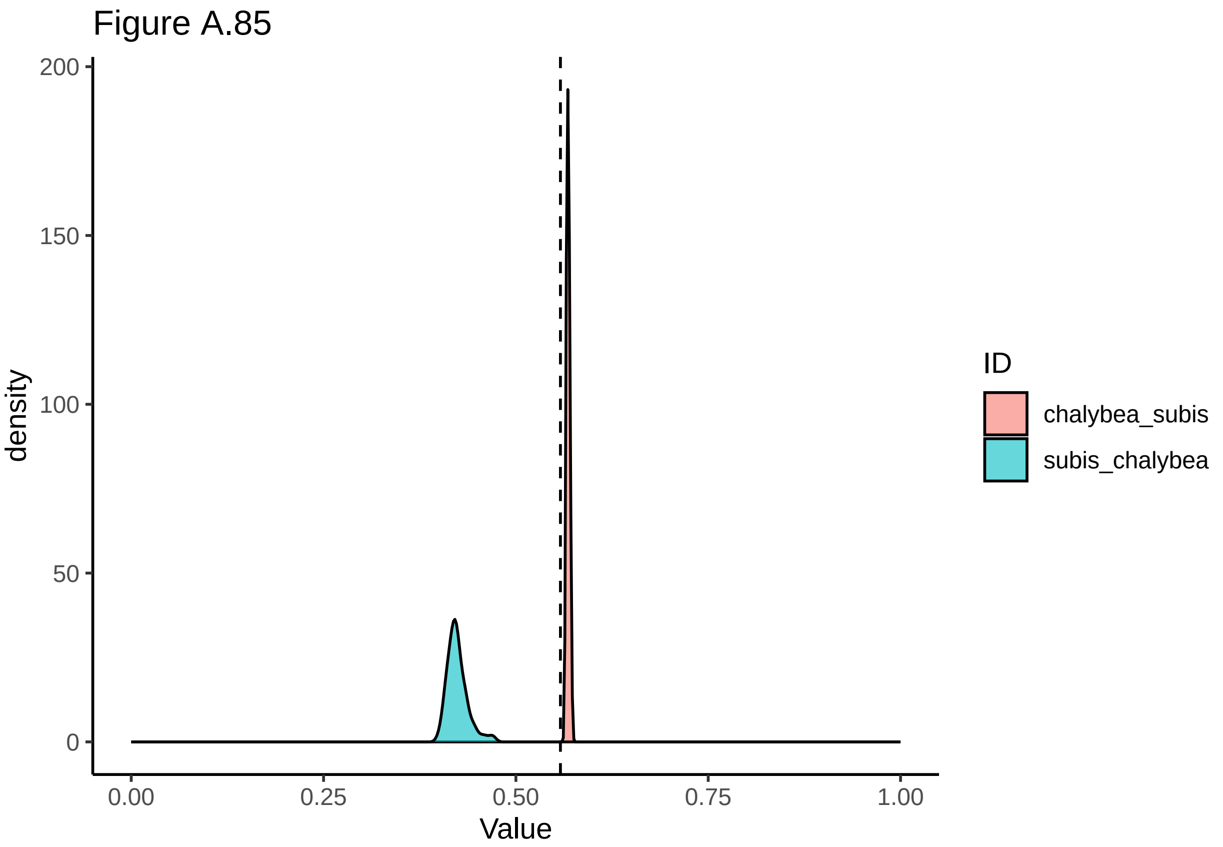


```
## [1] "subis_chalybea"
## [1] "P value for subis vs. chalybea = 1"
## [1] "Test statistic above high critical value."
## [1] "0.449945704057096; statistic = 0.557892897787648"
## [1] "chalybea_subis"
## [1] "P value for subis vs. chalybea = 1.10720012896822e-07"
## [1] "Test statistic below low critical value."
## [1] "0.564118732714916; statistic = 0.557892897787648"
```

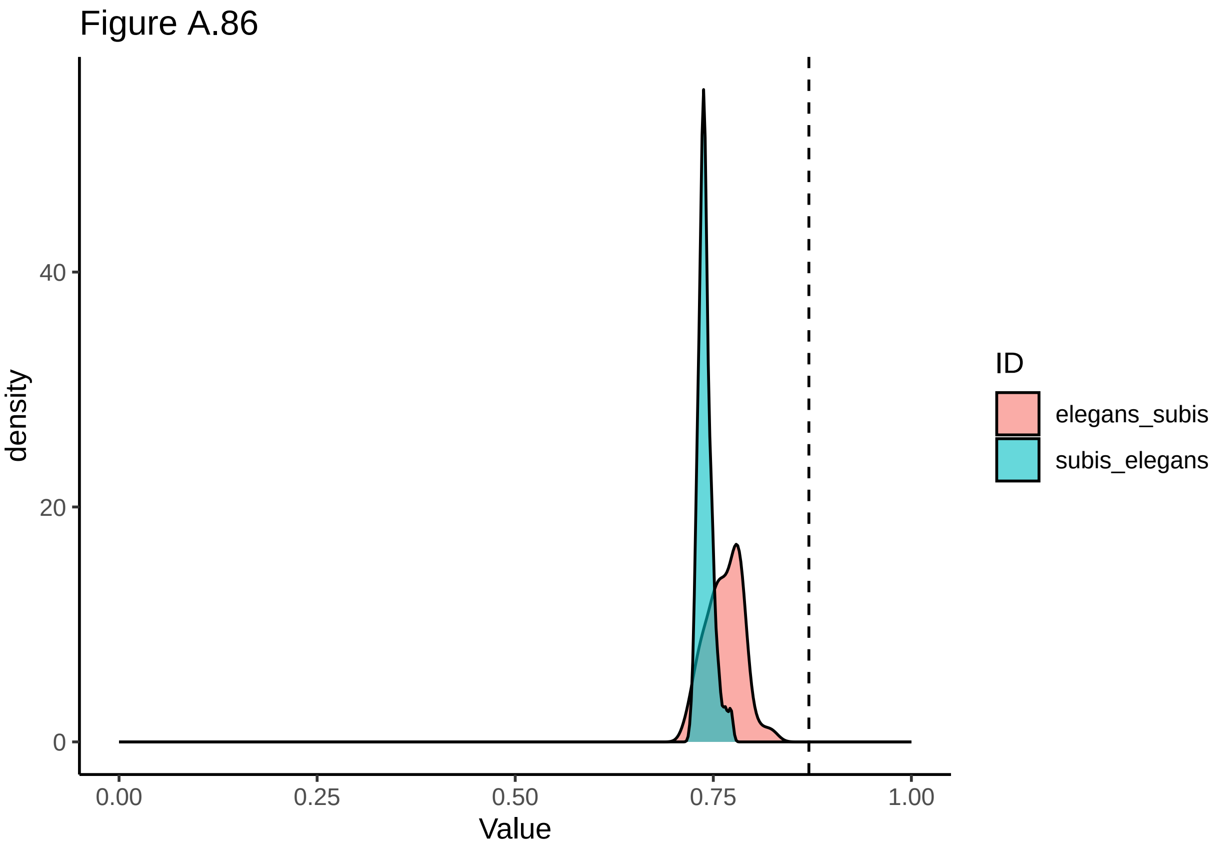


```
## [1] "subis_elegans"
## [1] "P value for subis vs. elegans = 1"
## [1] "Test statistic above high critical value."
## [1] "0.758251033354595; statistic = 0.870619103076361"
## [1] "elegans_subis"
## [1] "P value for subis vs. elegans = 0.999998746444586"
## [1] "Test statistic above high critical value."
## [1] "0.808541951133876; statistic = 0.870619103076361"
```

Figure A.87

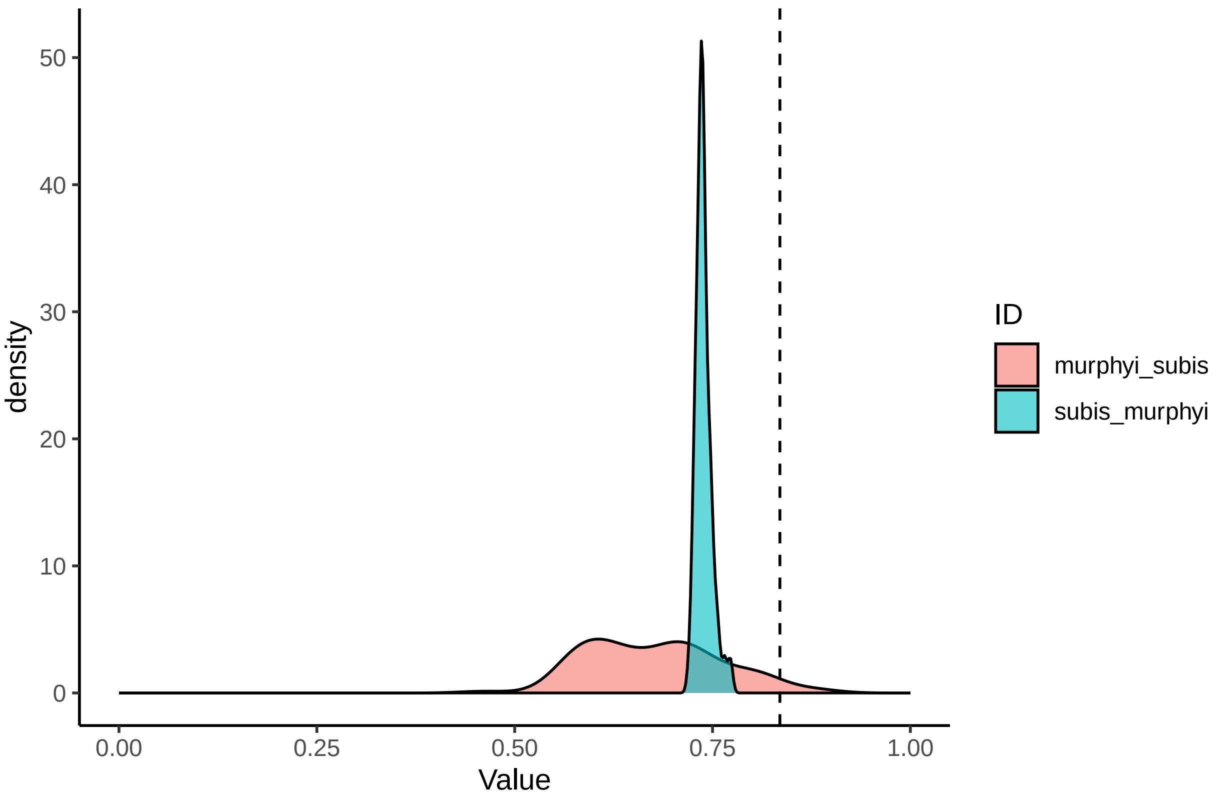


```
## [1] "subis_murphyi"
## [1] "P value for subis vs. murphyi = 1"
## [1] "Test statistic above high critical value."
## [1] "0.758298132126779; statistic = 0.835123293713782"
## [1] "murphyi_subis"
## [1] "P value for subis vs. murphyi = 0.971139295288171"
```

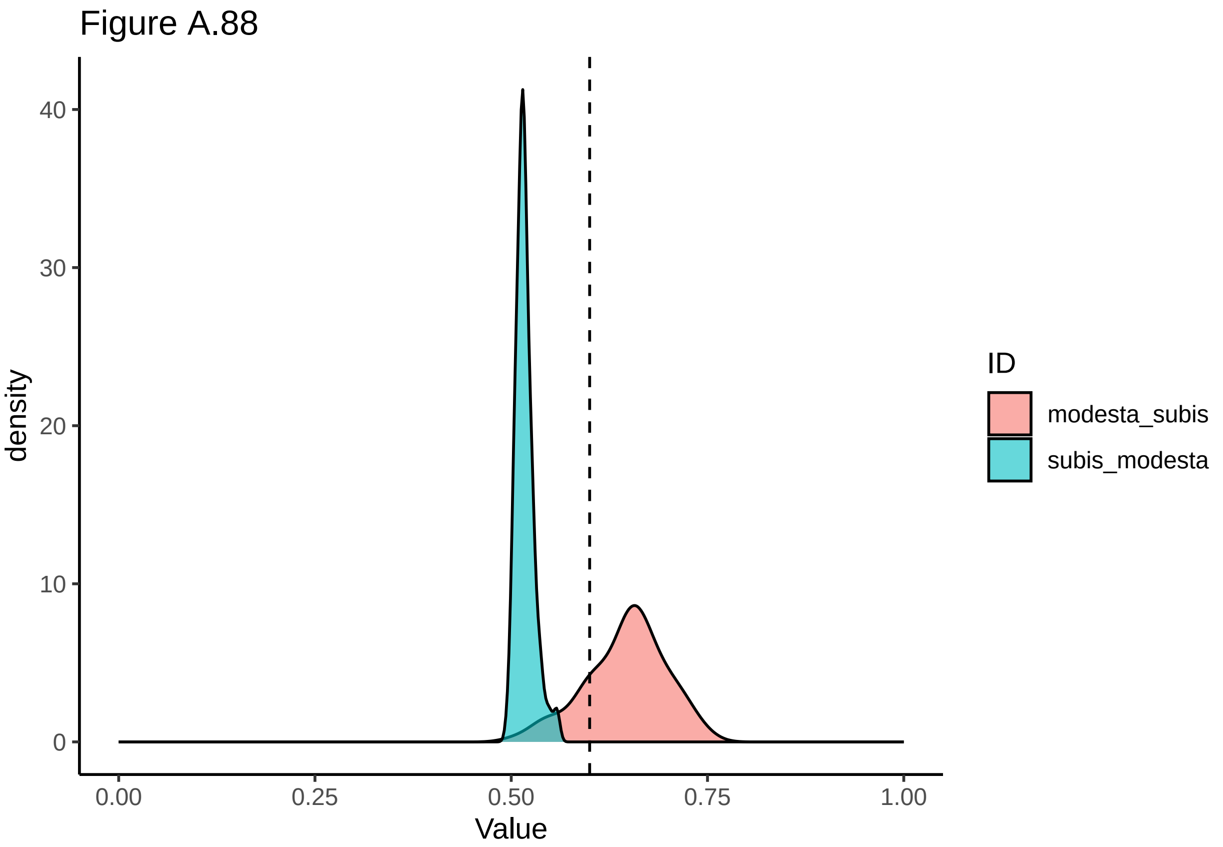


```
## [1] "subis_modesta"
## [1] "P value for subis vs. modesta = 0.999999999998774"
## [1] "Test statistic above high critical value."
## [1] "0.540528555485547; statistic = 0.599896377154647"
## [1] "modesta_subis"
## [1] "P value for subis vs. modesta = 0.181223380709725"
```

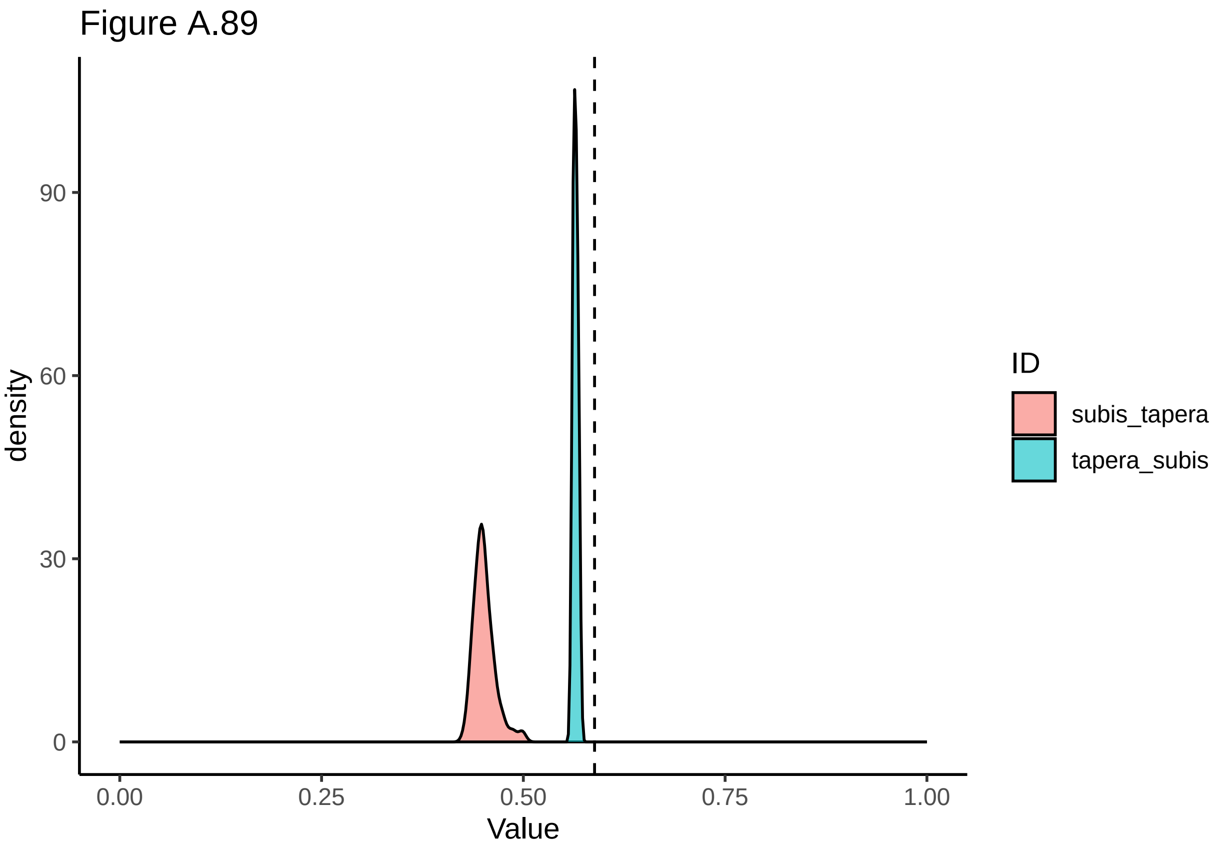


```
## [1] "subis_tapera"
## [1] "P value for subis vs. tapera = 1"
## [1] "Test statistic above high critical value."
## [1] "0.478120915348566; statistic = 0.588251057285956"
## [1] "tapera_subis"
## [1] "P value for subis vs. tapera = 0.999999999999996"
## [1] "Test statistic above high critical value."
## [1] "0.570620486311015; statistic = 0.588251057285956"
```

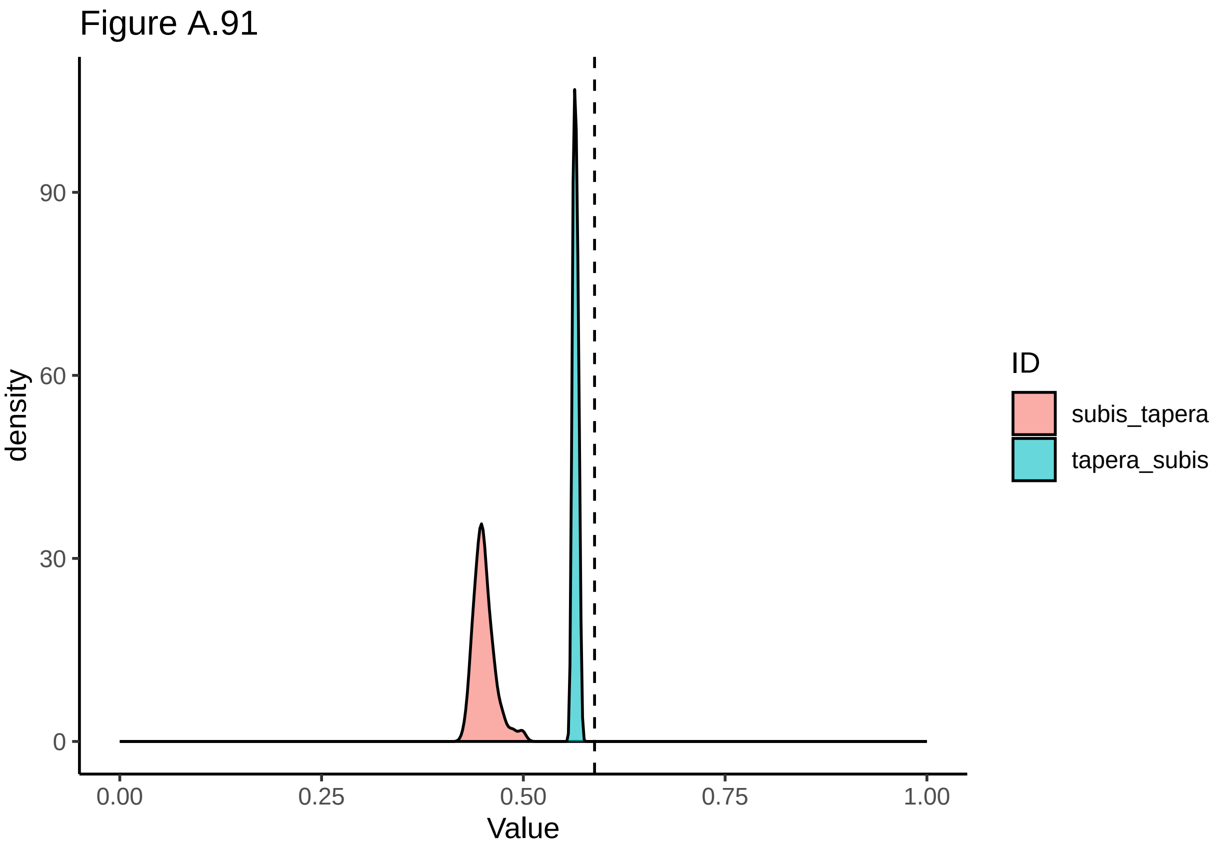


```
## [1] "tapera_subis"
## [1] "P value for tapera vs. subis = 0.999999999999996"
## [1] "Test statistic above high critical value."
## [1] "0.570620486311015; statistic = 0.588251057285956"
## [1] "subis_tapera"
## [1] "P value for tapera vs. subis = 1"
## [1] "Test statistic above high critical value."
## [1] "0.478120915348566; statistic = 0.588251057285956"
```

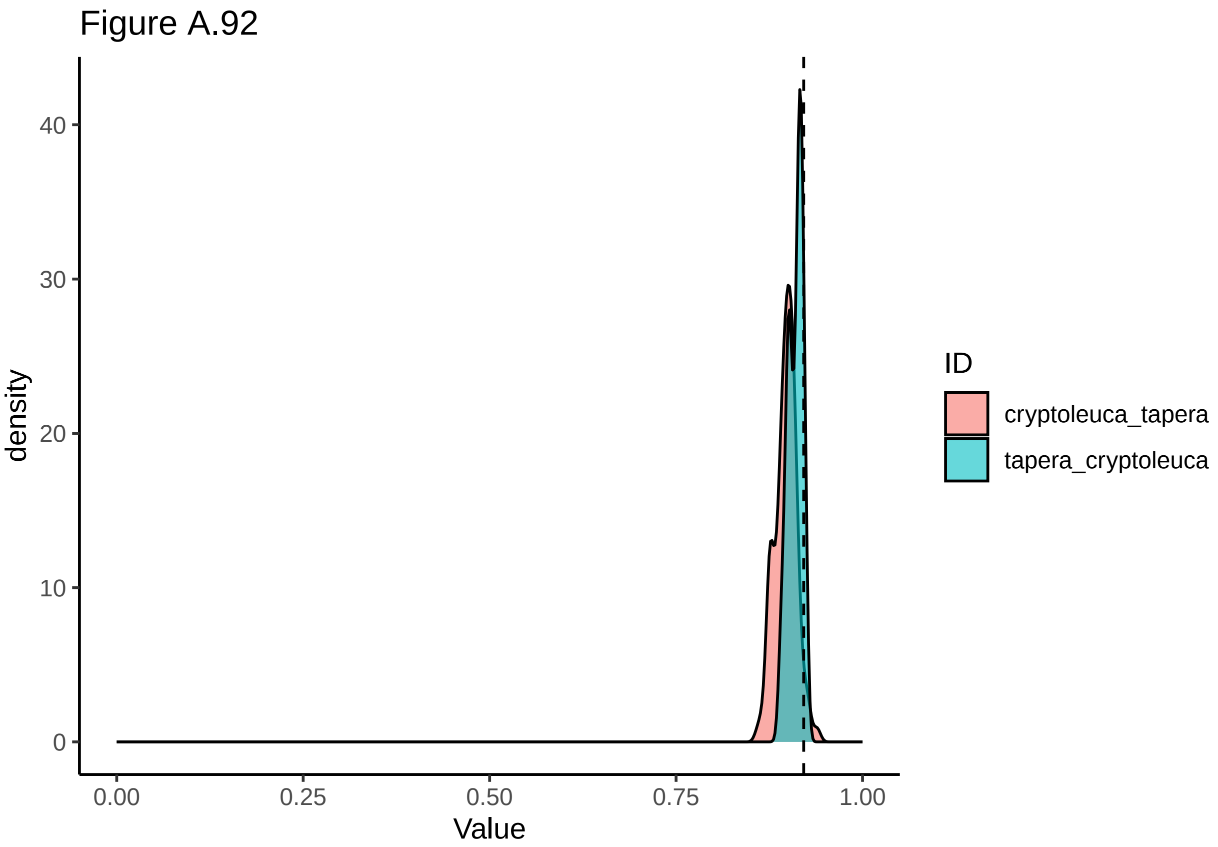


```
## [1] "tapera_cryptoleuca"
## [1] "P value for tapera vs. cryptoleuca = 0.881938628576874"
## [1] "cryptoleuca_tapera"
## [1] "P value for tapera vs. cryptoleuca = 0.953802915264648"
```

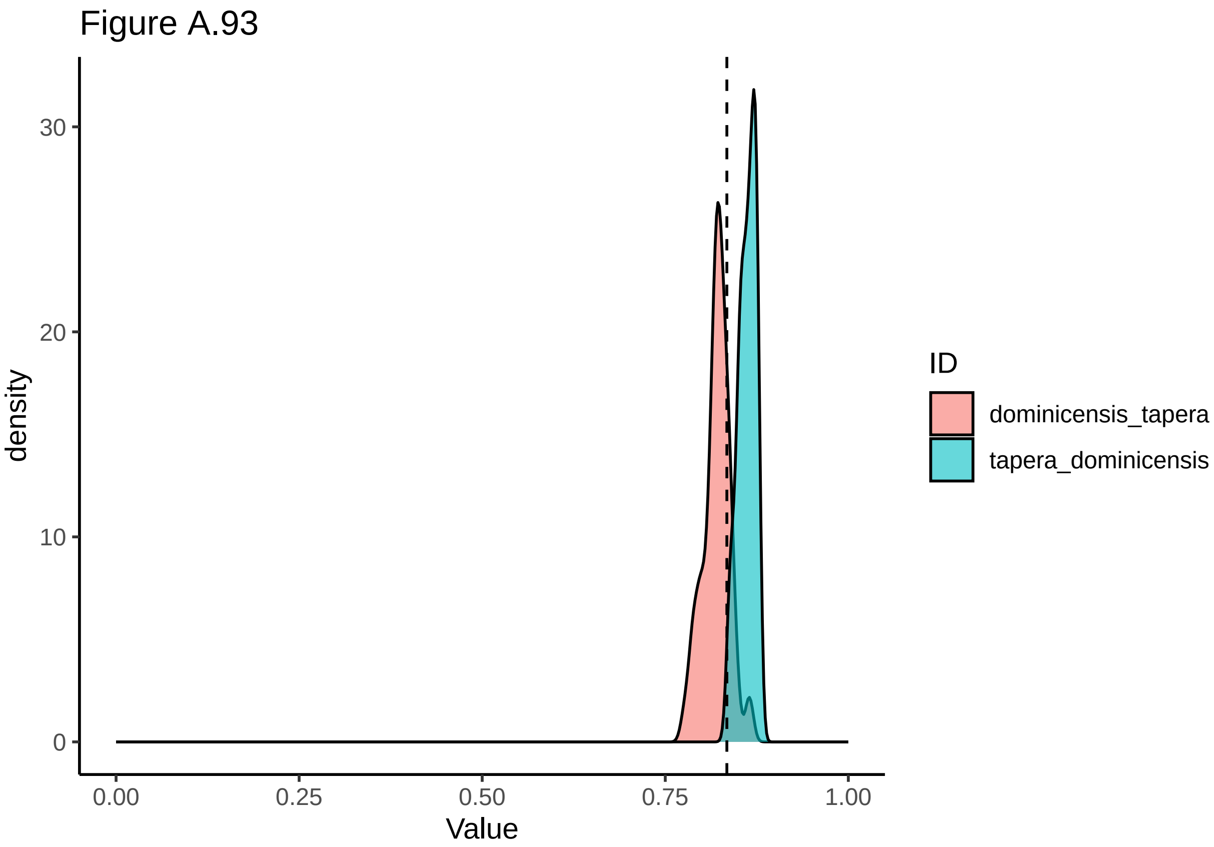


```
## [1] "tapera_dominicensis"
## [1] "P value for tapera vs. dominicensis = 0.00967468212857085"
## [1] "Test statistic below low critical value."
## [1] "0.83855636419014; statistic = 0.834135081207135"
## [1] "dominicensis_tapera"
## [1] "P value for tapera vs. dominicensis = 0.778955908690357"
```

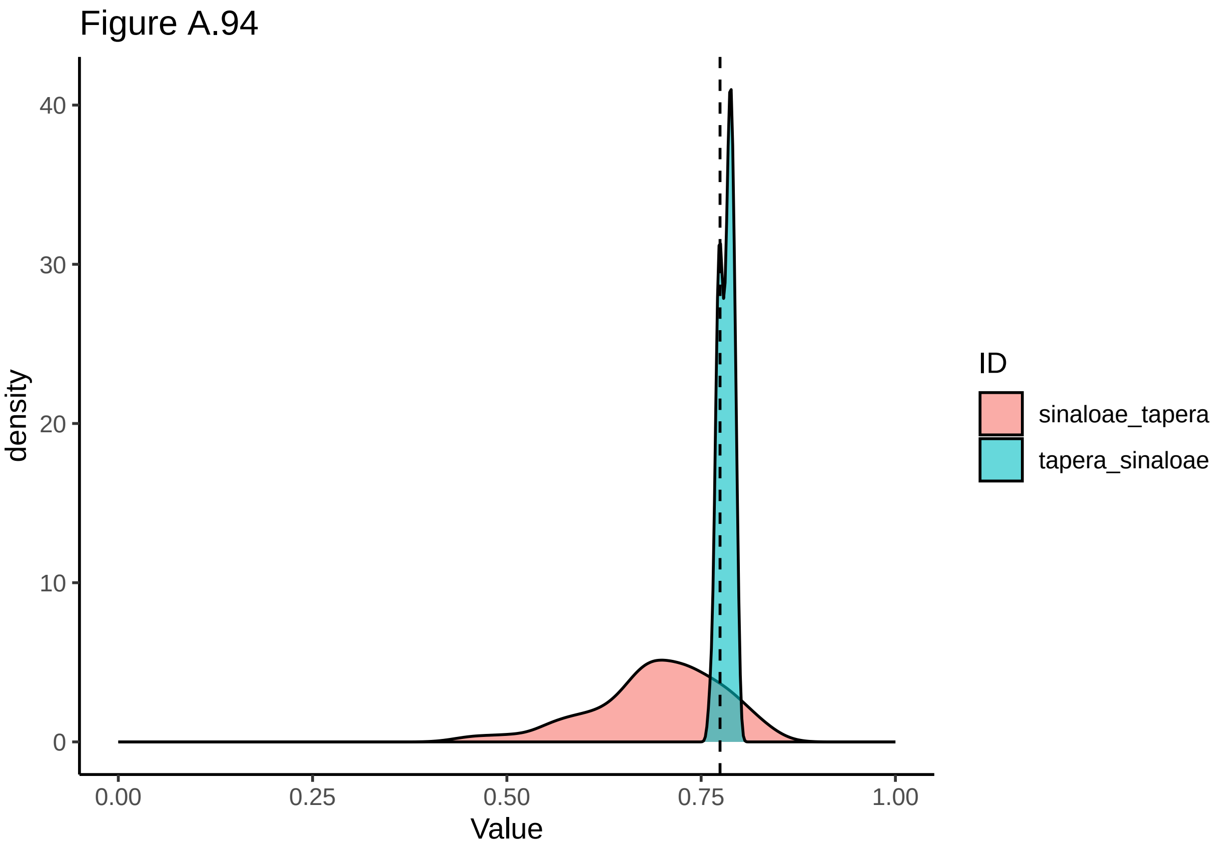


```
## [1] "tapera_sinaloae"
## [1] "P value for tapera vs. sinaloae = 0.188657928500043"
## [1] "sinaloae_tapera"
## [1] "P value for tapera vs. sinaloae = 0.837385351802245"
```

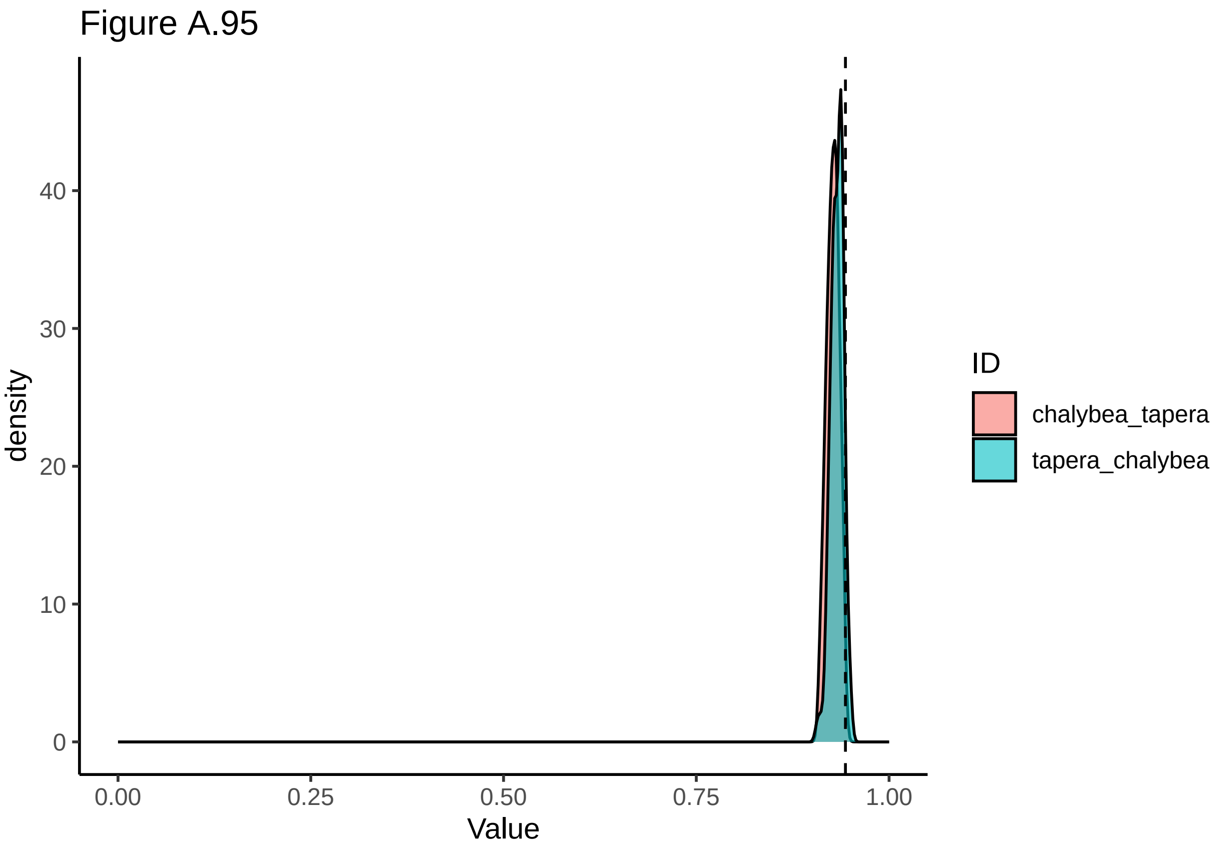


```
## [1] "tapera_chalybea"
## [1] "P value for tapera vs. chalybea = 0.909860928781084"
## [1] "chalybea_tapera"
## [1] "P value for tapera vs. chalybea = 0.980030480793911"
## [1] "Test statistic above high critical value."
## [1] "0.942700177395444; statistic = 0.943446311489337"
```

Figure A.96

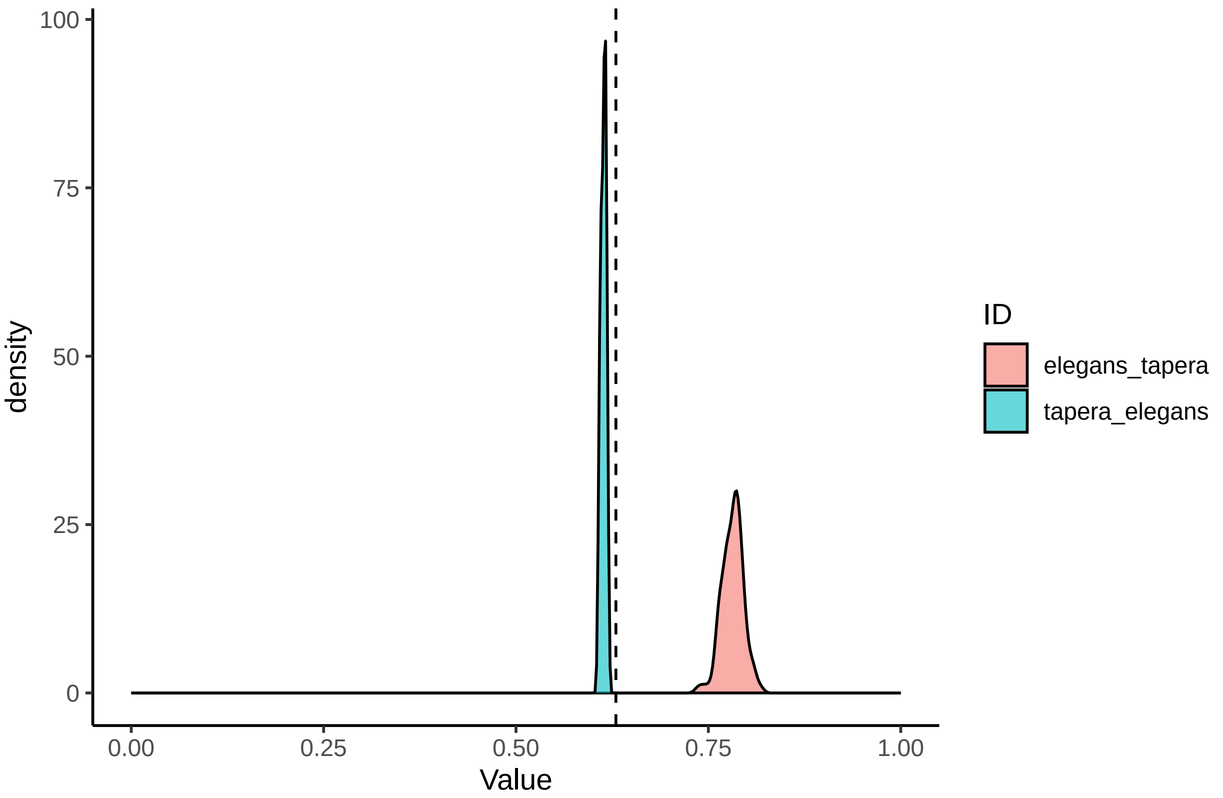


```
## [1] "tapera_elegans"
## [1] "P value for tapera vs. elegans = 0.999999195787371"
## [1] "Test statistic above high critical value."
## [1] "0.620340813315704; statistic = 0.629837909273023"
## [1] "elegans_tapera"
## [1] "P value for tapera vs. elegans = 1.78641501617912e-28"
## [1] "Test statistic below low critical value."
## [1] "0.754411253629397; statistic = 0.629837909273023"
```

Figure A.97

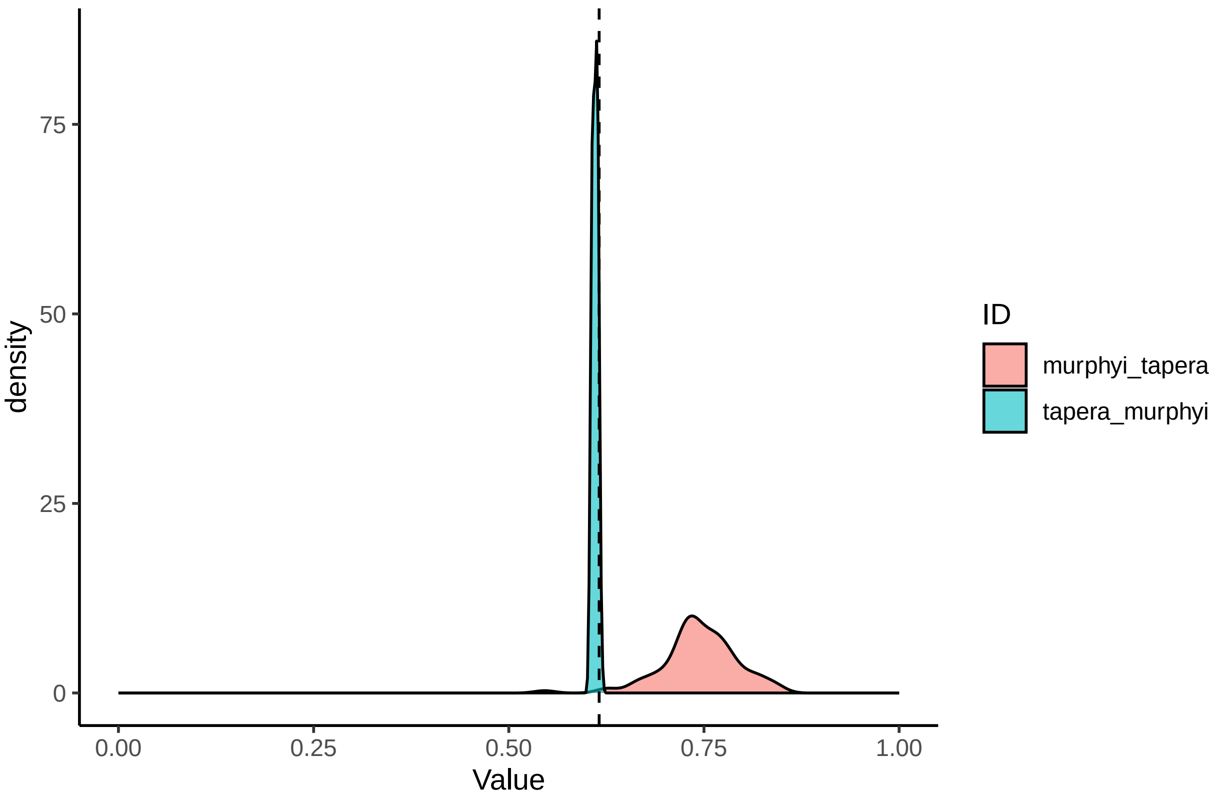


```
## [1] "tapera_murphyi"
## [1] "P value for tapera vs. murphyi = 0.923749328771478"
## [1] "murphyi_tapera"
## [1] "P value for tapera vs. murphyi = 0.00388454823008584"
## [1] "Test statistic below low critical value."
## [1] "0.649649760588276; statistic = 0.615724242493323"
```

Figure A.98

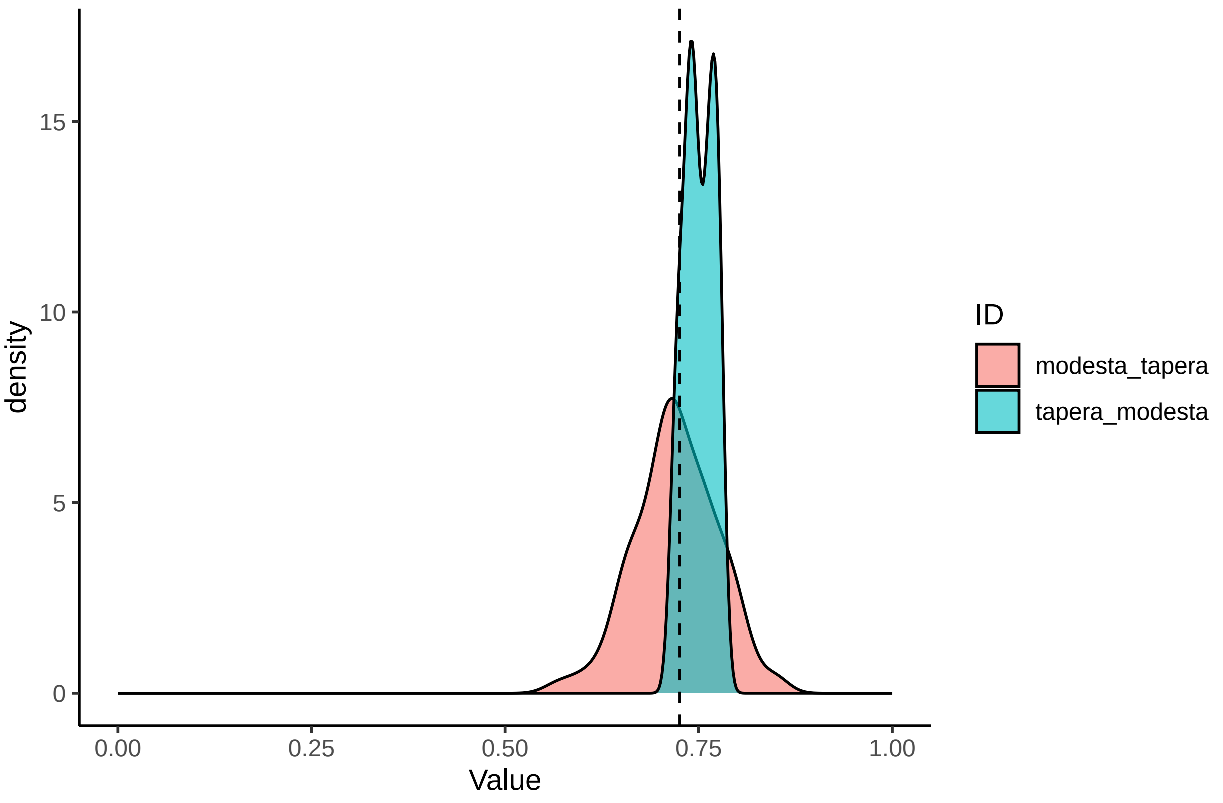


```
## [1] "tapera_modesta"
## [1] "P value for tapera vs. modesta = 0.0960970246071175"
## [1] "modesta_tapera"
## [1] "P value for tapera vs. modesta = 0.535747443004034"
```

B: Subspecies comparisons

```
for(i in 1:length(subis.comp.files)){
  sp=strsplit(subis.comp.files[i],"_")[[1]][1]
  x1=read_csv(paste0(filepath,subis.comp.files[i]))
  for(ii in 1:ncol(x1)){
    comp.sp=strsplit(colnames(x1)[ii],"_")[[1]][3]
    list.1=x1[,ii]

    if(comp.sp==sp){next}
    x2=read_csv(paste0(filepath,subis.comp.files[subis.comp.files%like%comp.sp]))
    list.2=x2[-1,which(colnames(x2)%like%sp)]

    list.1$ID=paste0(sp,'_',comp.sp)
    list.2$ID=paste0(comp.sp,'_',sp)

    test_val=list.1[1,1]
    list.1=list.1[-1,]

    colnames(list.1)=colnames(list.2)=c("Value","ID")

    val.list=as.data.frame(rbind(list.1,list.2))
```

```
    a=ggplot(data=val.list,aes(x=Value,fill=ID))
    b=scale_x_continuous(limits=c(0,1))
    c=geom_density(alpha=0.6)
    d=theme_classic()
    d2=geom_vline(xintercept = as.numeric(test_val),colour="black",
                  linetype="dashed")
    d3=ggtitle(paste0("Figure B.",i,ii))

    print(a+b+c+d+d2+d3)

    IDs=unique(val.list$ID)

    for(iii in 1:2){
      ID=IDs[iii]
      print(ID)
      sub.val=val.list[which(val.list$ID==ID),]
      mu=mean(sub.val$Value)
      sigma=sd(sub.val$Value)
      n=nrow(sub.val)

      xbar=as.numeric(test_val)

      z=(xbar-mu)/(sigma/sqrt(n))

      lowcrit=qnorm(p=0.025,mean=mu,sd=sigma)
      hicrit=qnorm(p=0.975,mean=mu,sd=sigma)

      print(paste0("P value for ",sp," vs. ",comp.sp,
                   " = ",pnorm(xbar,
                               mean=mu,sd=sigma)))

      if(xbar<lowcrit){
        print("Test statistic below low critical value.")
        print(paste0(lowcrit,"; statistic = ",xbar))
        next
      }
      if(xbar>hicrit){
        print("Test statistic above high critical value.")
        print(paste0(hicrit,"; statistic = ",xbar))
        next
      }
    }
  }
}
```

Figure B.11

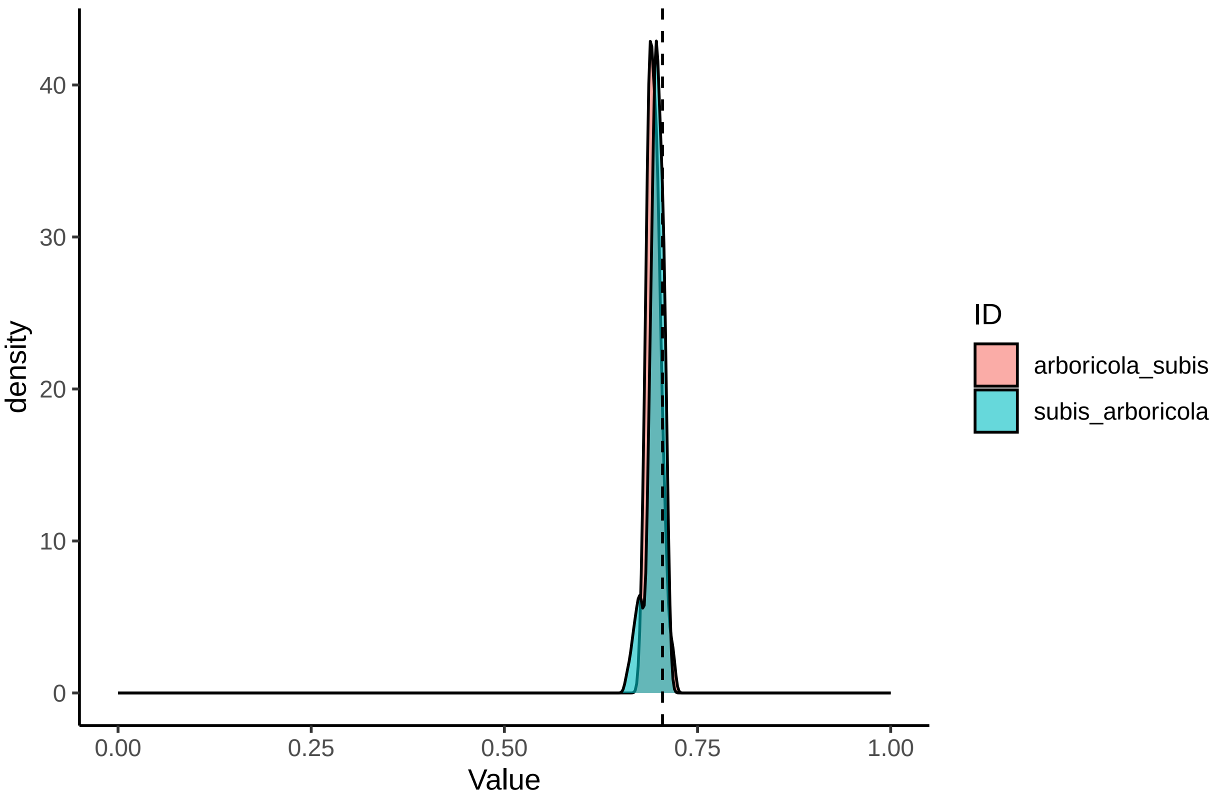


```
## [1] "arboricola_subis"
## [1] "P value for arboricola vs. subis = 0.897214018750521"
## [1] "subis_arboricola"
## [1] "P value for arboricola vs. subis = 0.801150285765002"
```

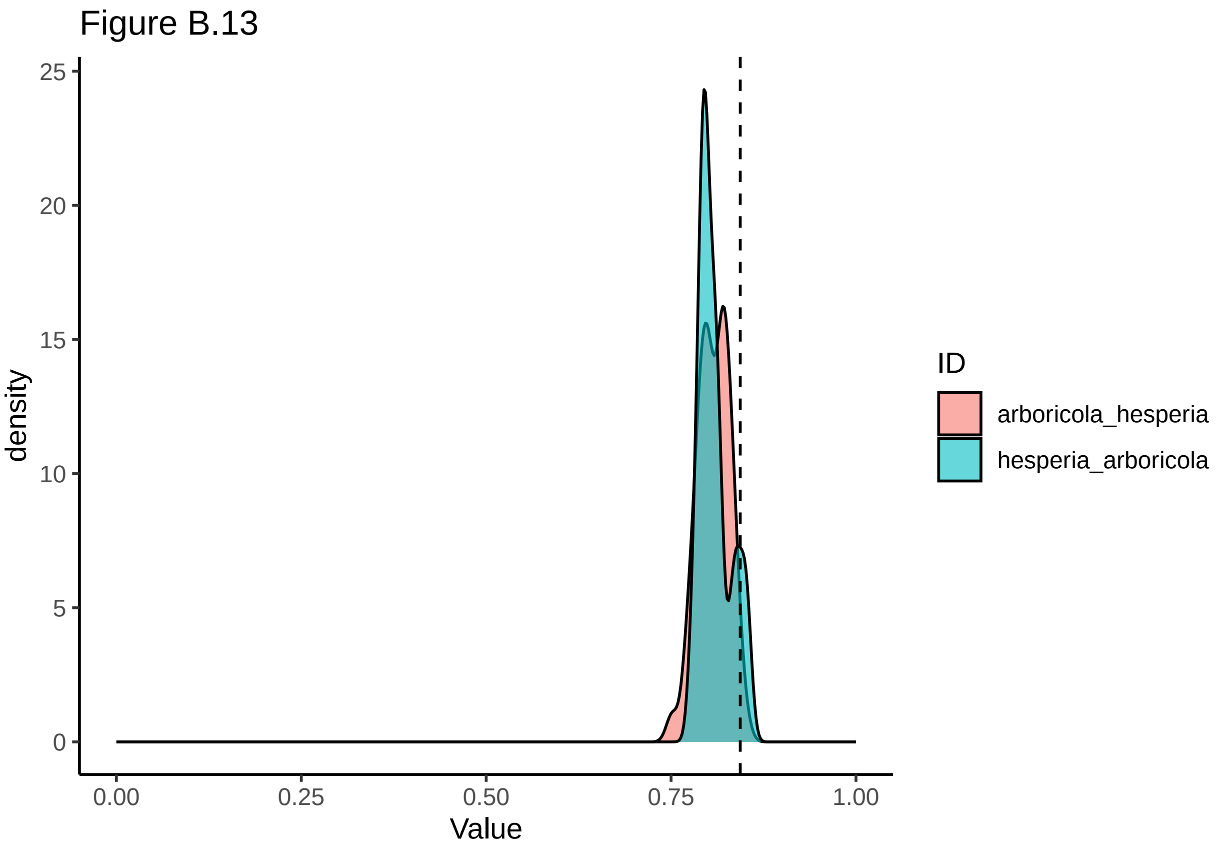


```
## [1] "arboricola_hesperia"
## [1] "P value for arboricola vs. hesperia = 0.958304222864935"
## [1] "hesperia_arboricola"
## [1] "P value for arboricola vs. hesperia = 0.951036130670033"
```

Figure B.14

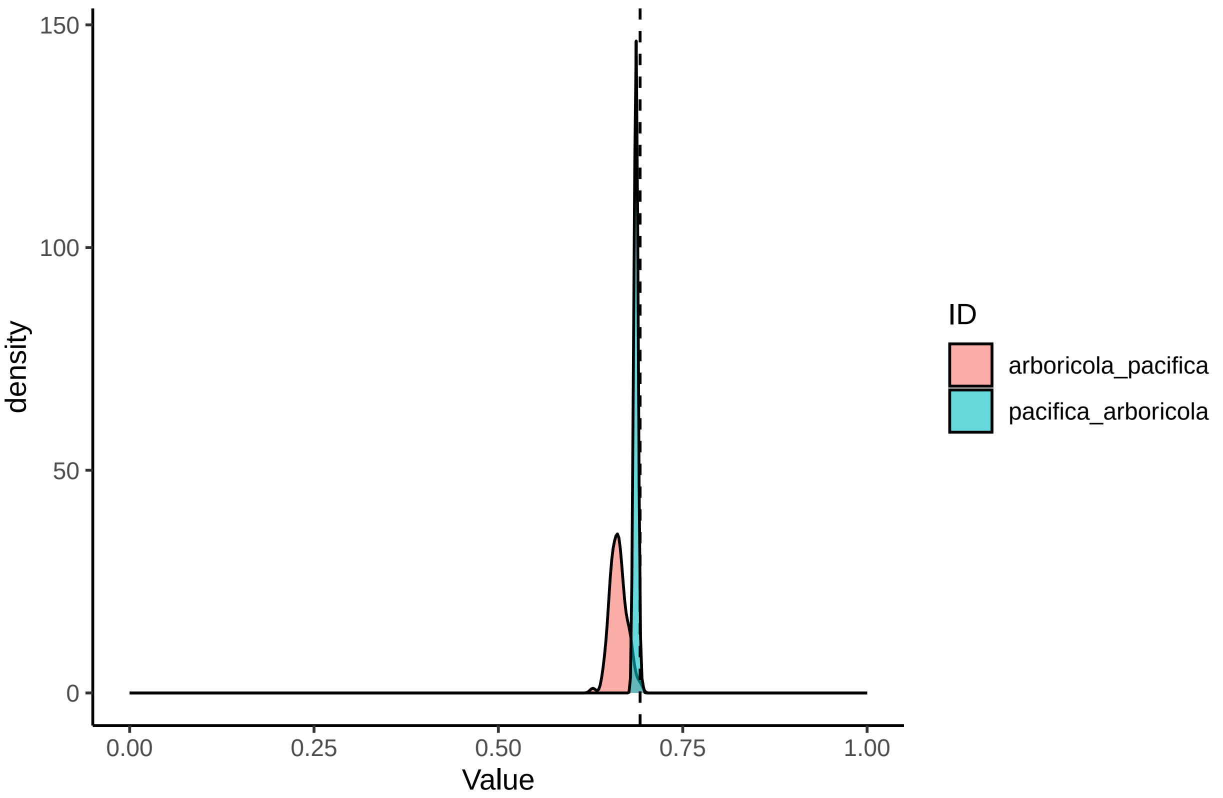


```
## [1] "arboricola_pacifica"
## [1] "P value for arboricola vs. pacifica = 0.995921993929447"
## [1] "Test statistic above high critical value."
## [1] "0.684491016861398; statistic = 0.692175835838449"
## [1] "pacifica_arboricola"
## [1] "P value for arboricola vs. pacifica = 0.98656810582395"
## [1] "Test statistic above high critical value."
## [1] "0.691532025784251; statistic = 0.692175835838449"
```

Figure B.15

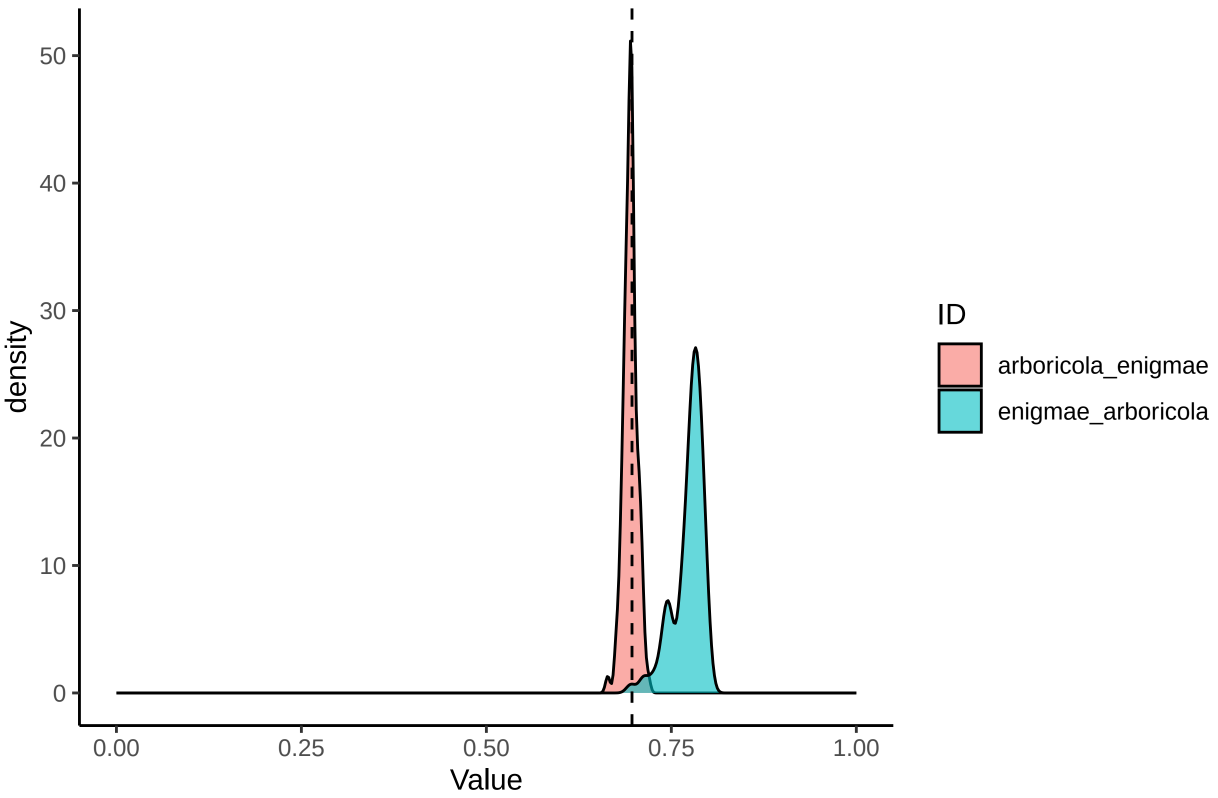


```
## [1] "arboricola_enigmae"
## [1] "P value for arboricola vs. enigmae = 0.605488596325206"
## [1] "enigmae_arboricola"
## [1] "P value for arboricola vs. enigmae = 0.000125633706492037"
## [1] "Test statistic below low critical value."
## [1] "0.732169888846646; statistic = 0.696818752956035"
```

Figure B.21

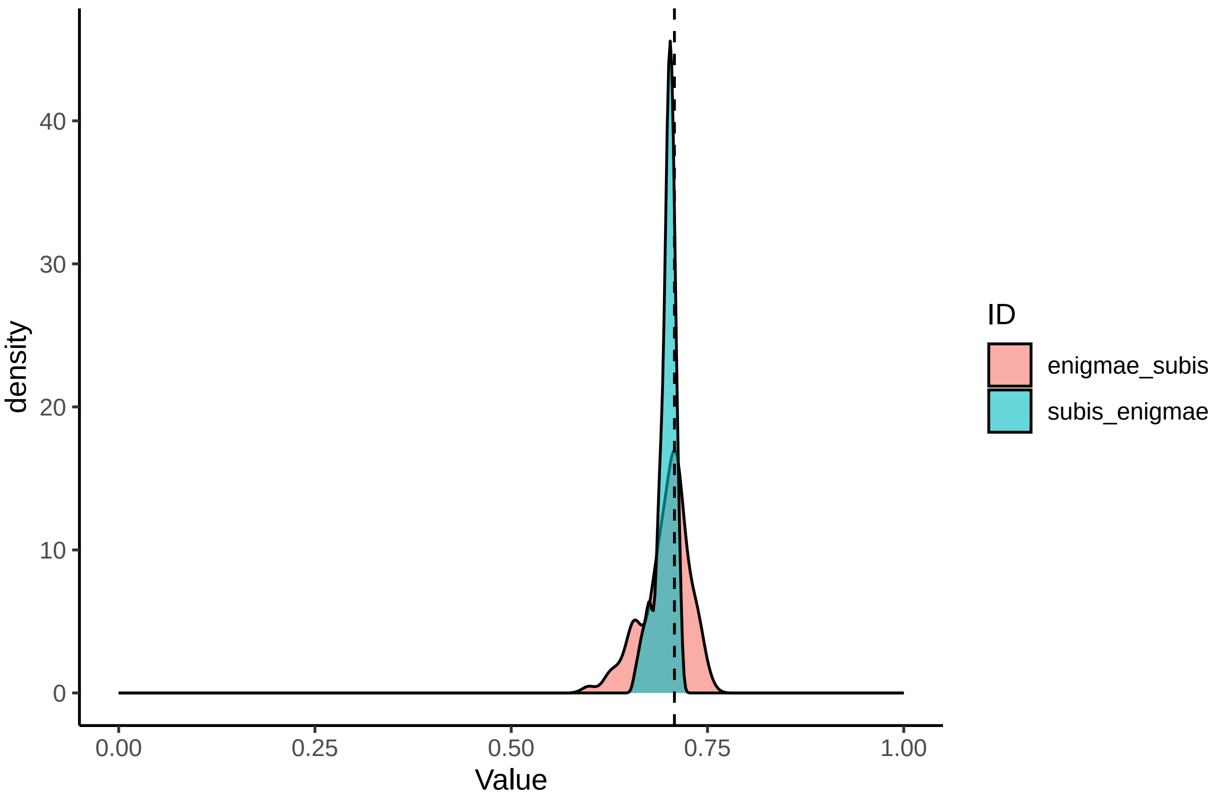


```
## [1] "enigmae_subis"
## [1] "P value for enigmae vs. subis = 0.643415185715688"
## [1] "subis_enigmae"
## [1] "P value for enigmae vs. subis = 0.805847702903804"
```

Figure B.22

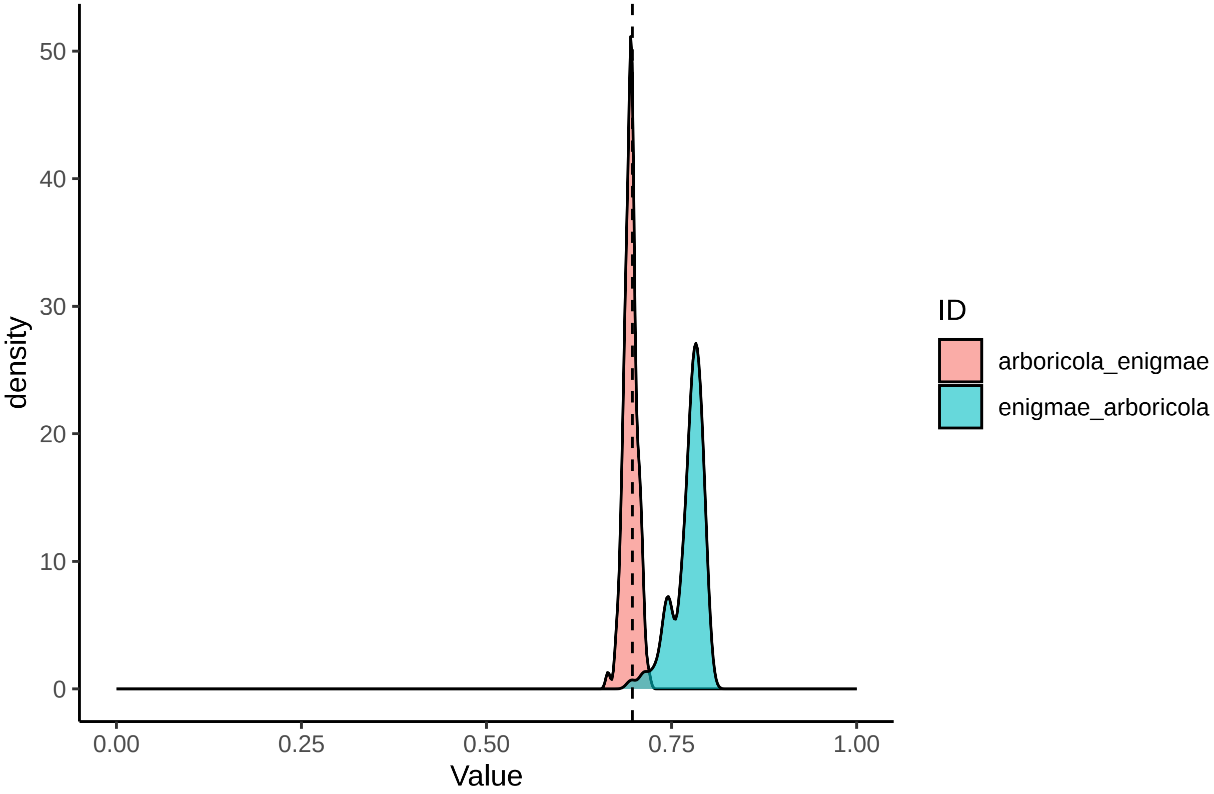


```
## [1] "enigmae_arboricola"
## [1] "P value for enigmae vs. arboricola = 0.000125633706492037"
## [1] "Test statistic below low critical value."
## [1] "0.732169888846646; statistic = 0.696818752956035"
## [1] "arboricola_enigmae"
## [1] "P value for enigmae vs. arboricola = 0.605488596325206"
```

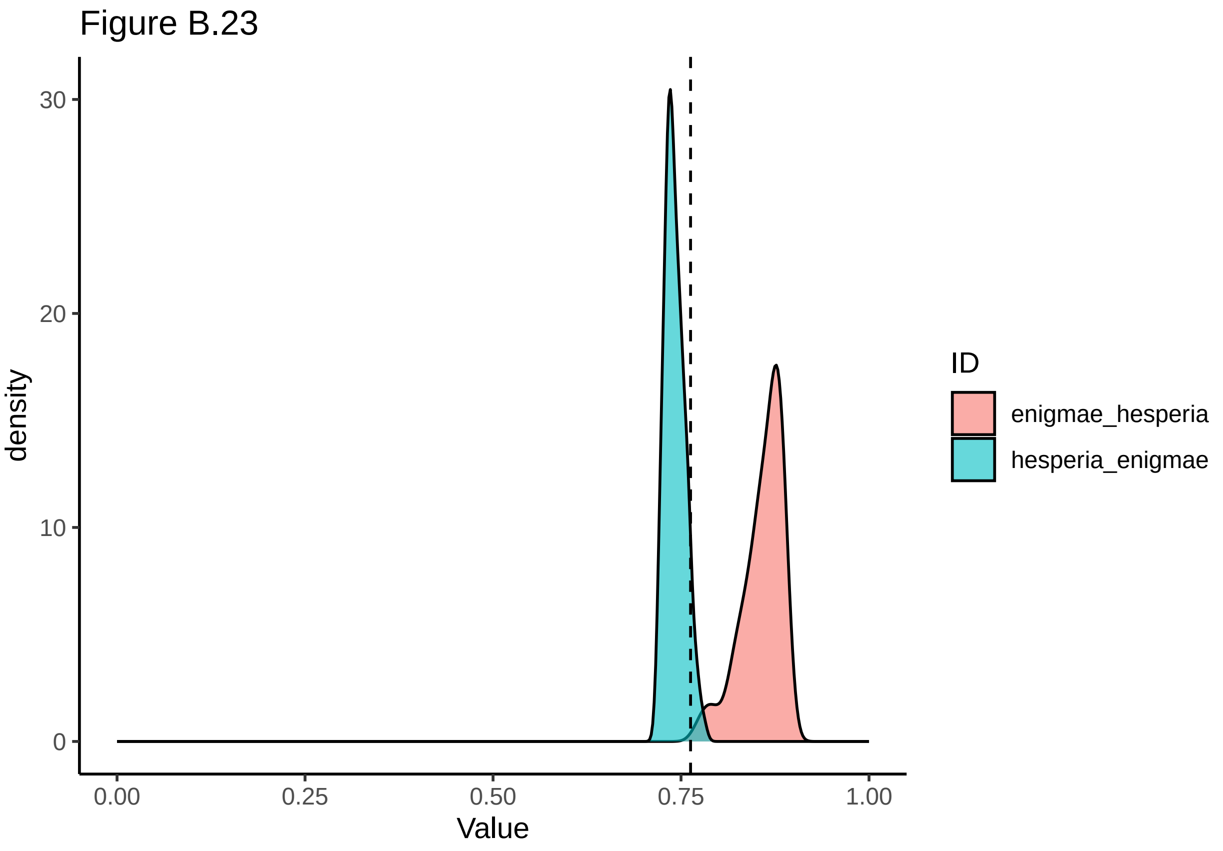


```
## [1] "enigmae_hesperia"
## [1] "P value for enigmae vs. hesperia = 0.000235903781827755"
## [1] "Test statistic below low critical value."
## [1] "0.804383230678103; statistic = 0.762675562886749"
## [1] "hesperia_enigmae"
## [1] "P value for enigmae vs. hesperia = 0.945650775417108"
```

Figure B.24

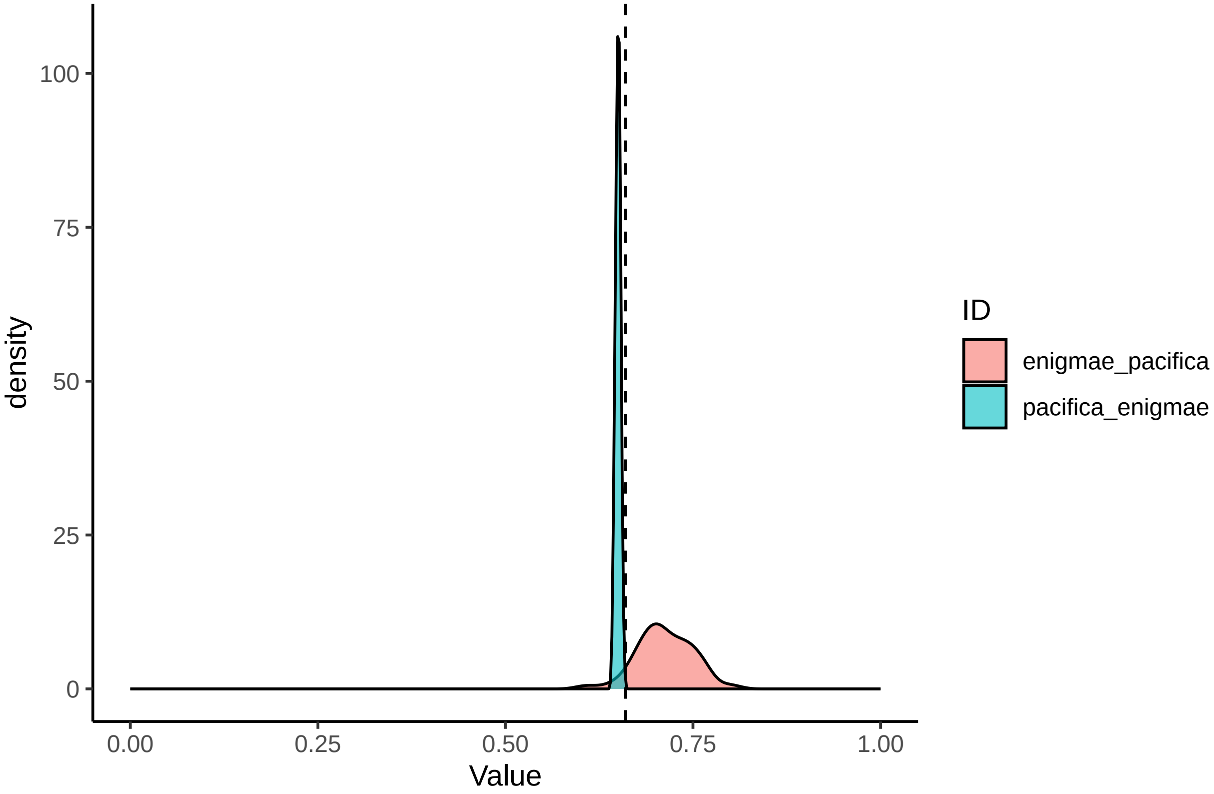


```
## [1] "enigmae_pacifica"
## [1] "P value for enigmae vs. pacifica = 0.0750848771079954"
## [1] "pacifica_enigmae"
## [1] "P value for enigmae vs. pacifica = 0.999118403218665"
## [1] "Test statistic above high critical value."
## [1] "0.656257251372496; statistic = 0.659932933213162"
```

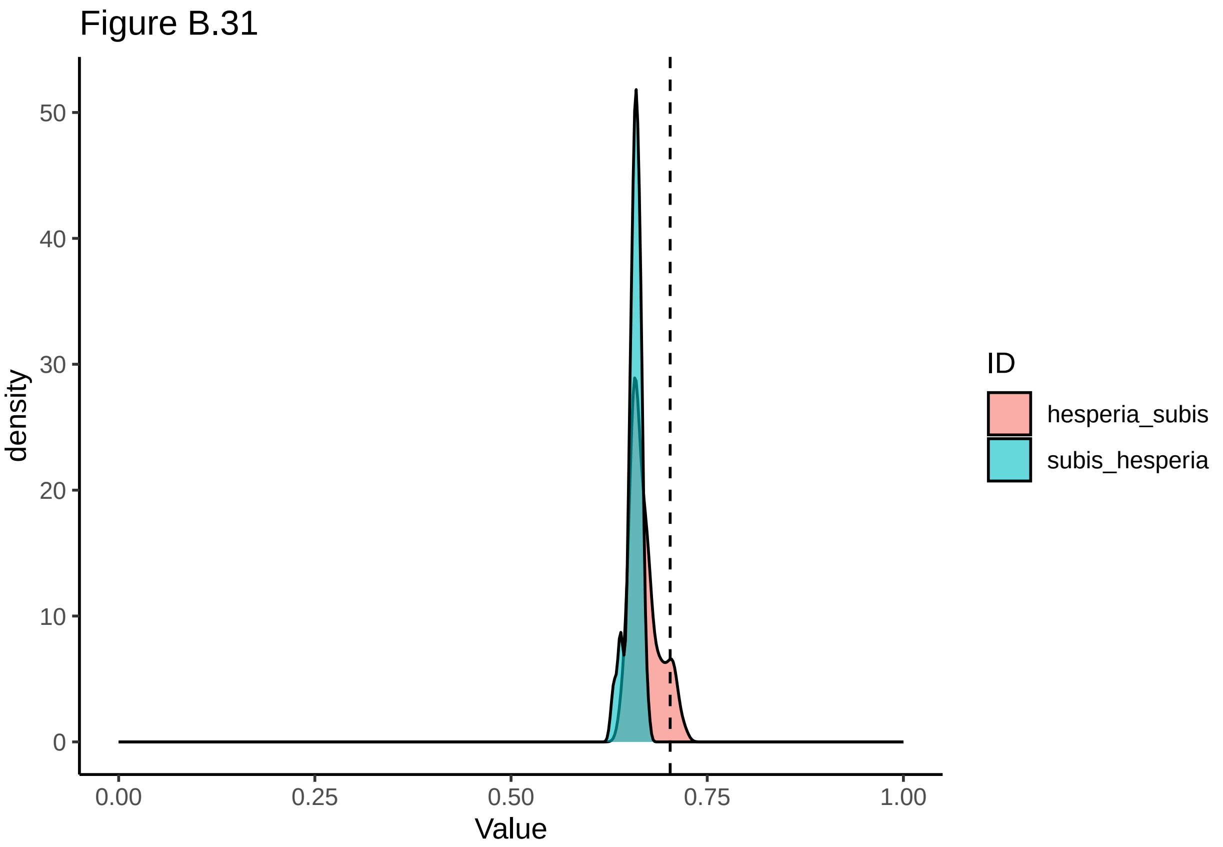


```
## [1] "hesperia_subis"
## [1] "P value for hesperia vs. subis = 0.963250045671483"
## [1] "subis_hesperia"
## [1] "P value for hesperia vs. subis = 0.999999562151269"
## [1] "Test statistic above high critical value."
## [1] "0.675132144914459; statistic = 0.702759858381477"
```

Figure B.32

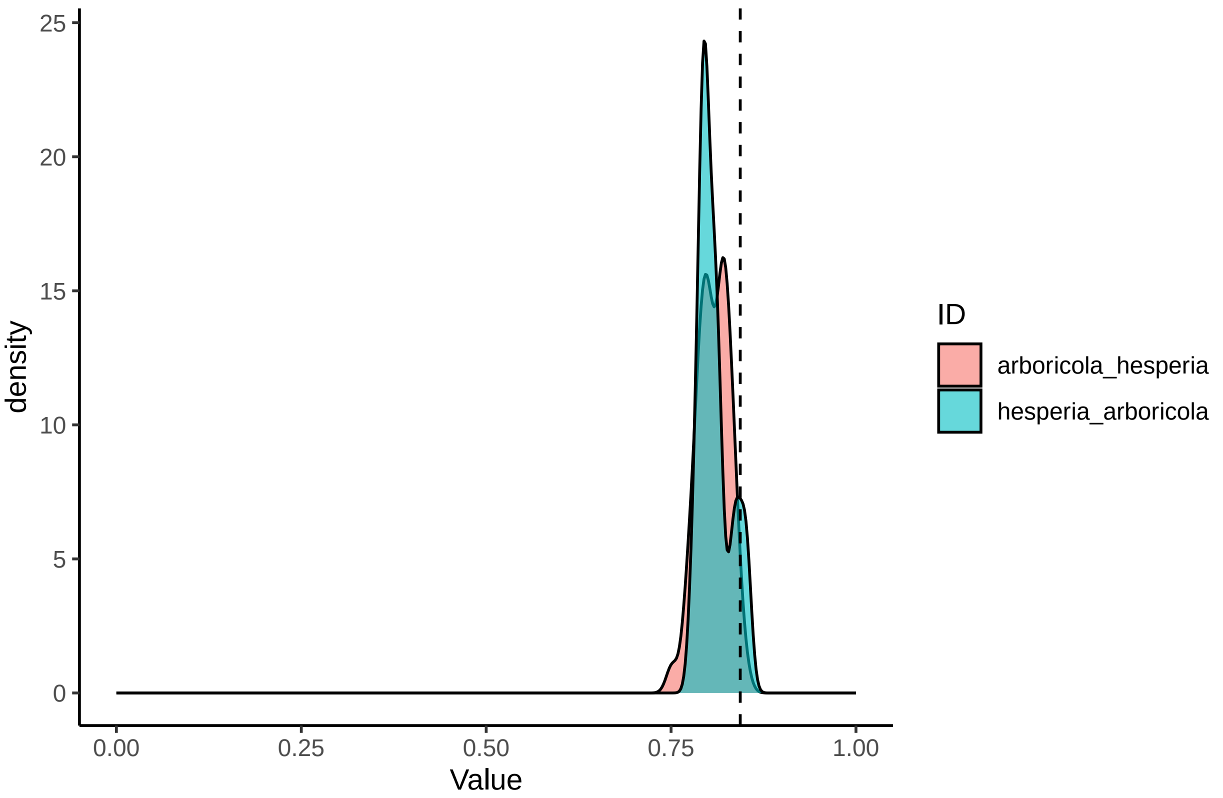


```
## [1] "hesperia_arboricola"
## [1] "P value for hesperia vs. arboricola = 0.951036130670033"
## [1] "arboricola_hesperia"
## [1] "P value for hesperia vs. arboricola = 0.958304222864935"
```

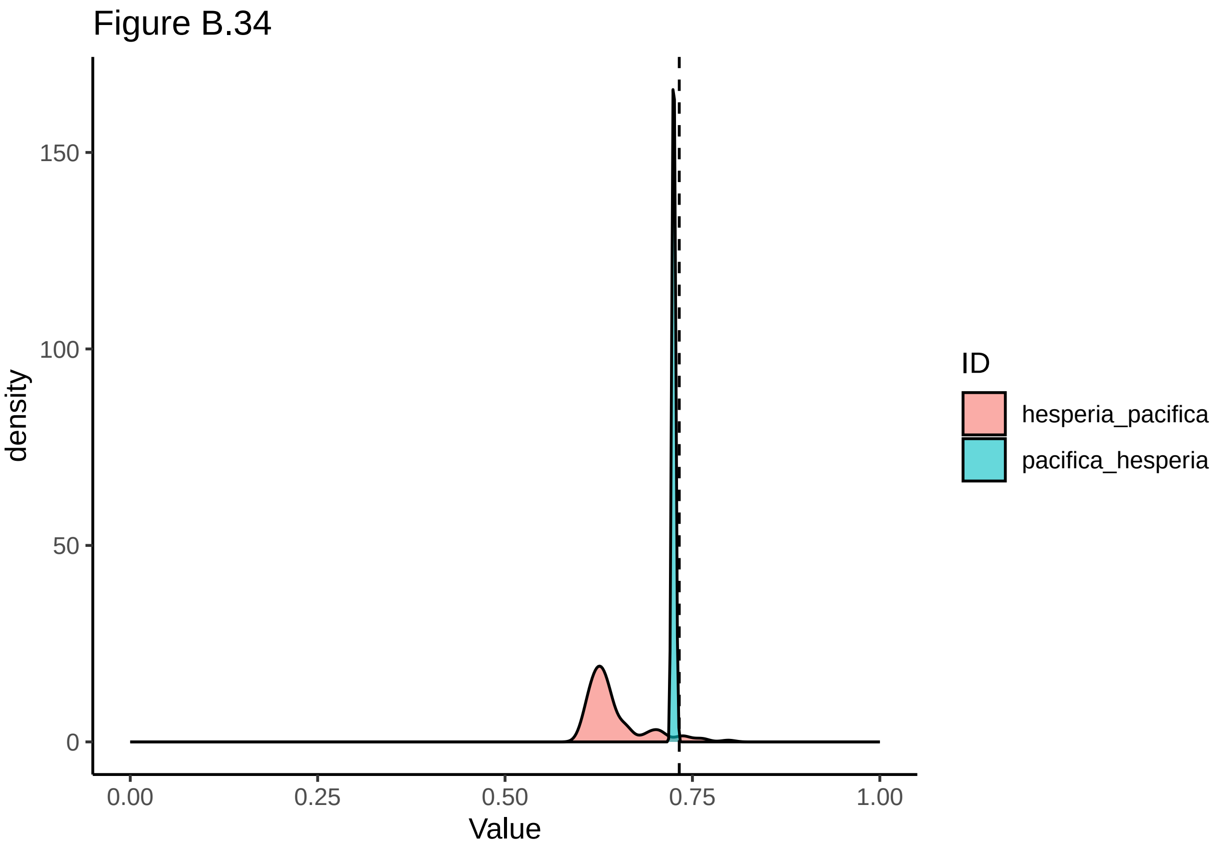


```
## [1] "hesperia_pacifica"
## [1] "P value for hesperia vs. pacifica = 0.985861219536583"
## [1] "Test statistic above high critical value."
## [1] "0.723152555455607; statistic = 0.732404918264699"
## [1] "pacifica_hesperia"
## [1] "P value for hesperia vs. pacifica = 0.999810707015926"
## [1] "Test statistic above high critical value."
## [1] "0.729138438790516; statistic = 0.732404918264699"
```

Figure B.35

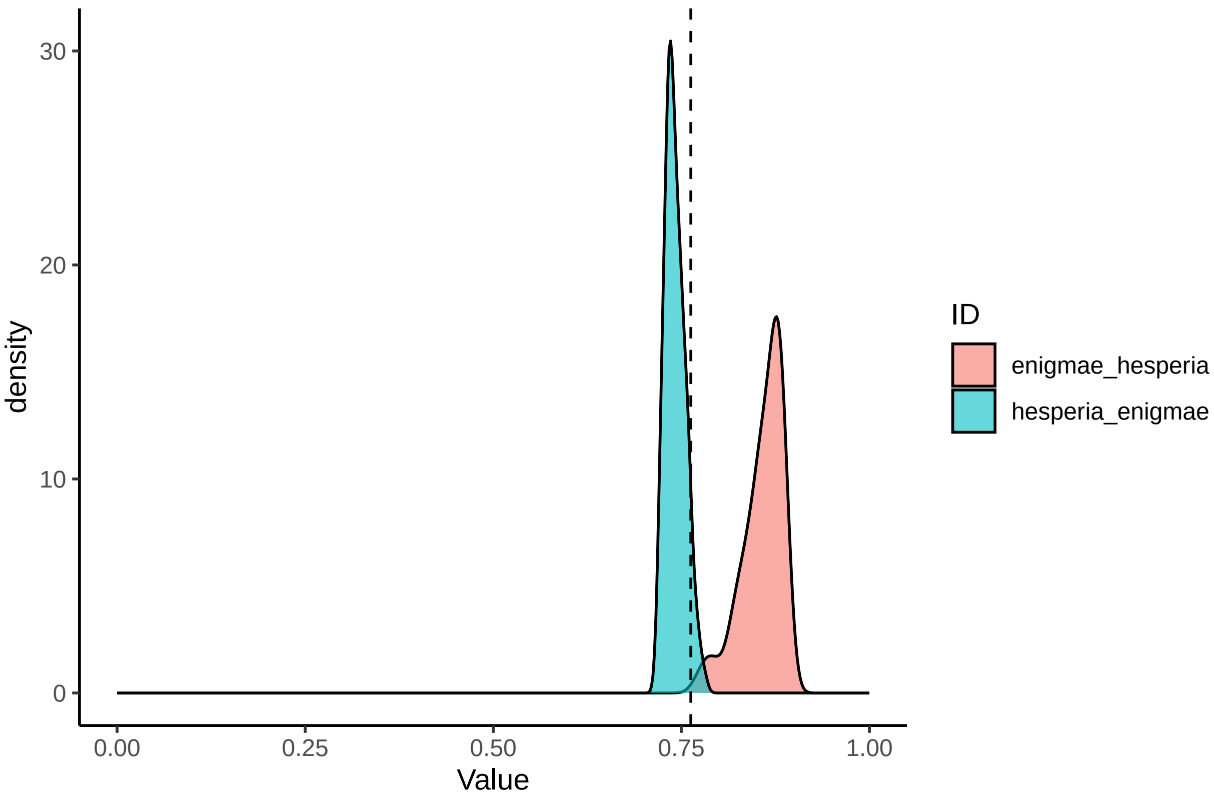


```
## [1] "hesperia_enigmae"
## [1] "P value for hesperia vs. enigmae = 0.945650775417108"
## [1] "enigmae_hesperia"
## [1] "P value for hesperia vs. enigmae = 0.000235903781827755"
## [1] "Test statistic below low critical value."
## [1] "0.804383230678103; statistic = 0.762675562886749"
```

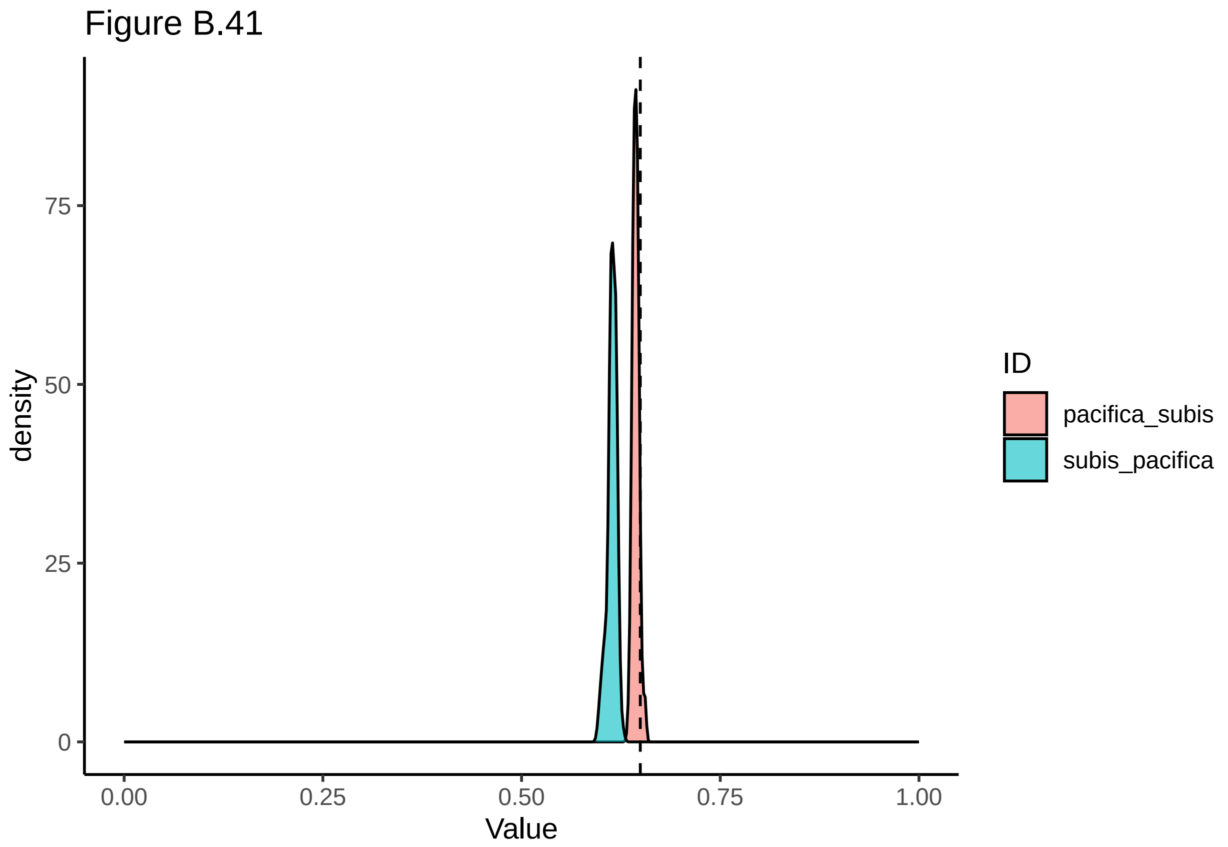


```
## [1] "pacifica_subis"
## [1] "P value for pacifica vs. subis = 0.923700723743345"
## [1] "subis_pacifica"
## [1] "P value for pacifica vs. subis = 0.99999999961522"
## [1] "Test statistic above high critical value."
## [1] "0.625228933535226; statistic = 0.649373031253288"
```

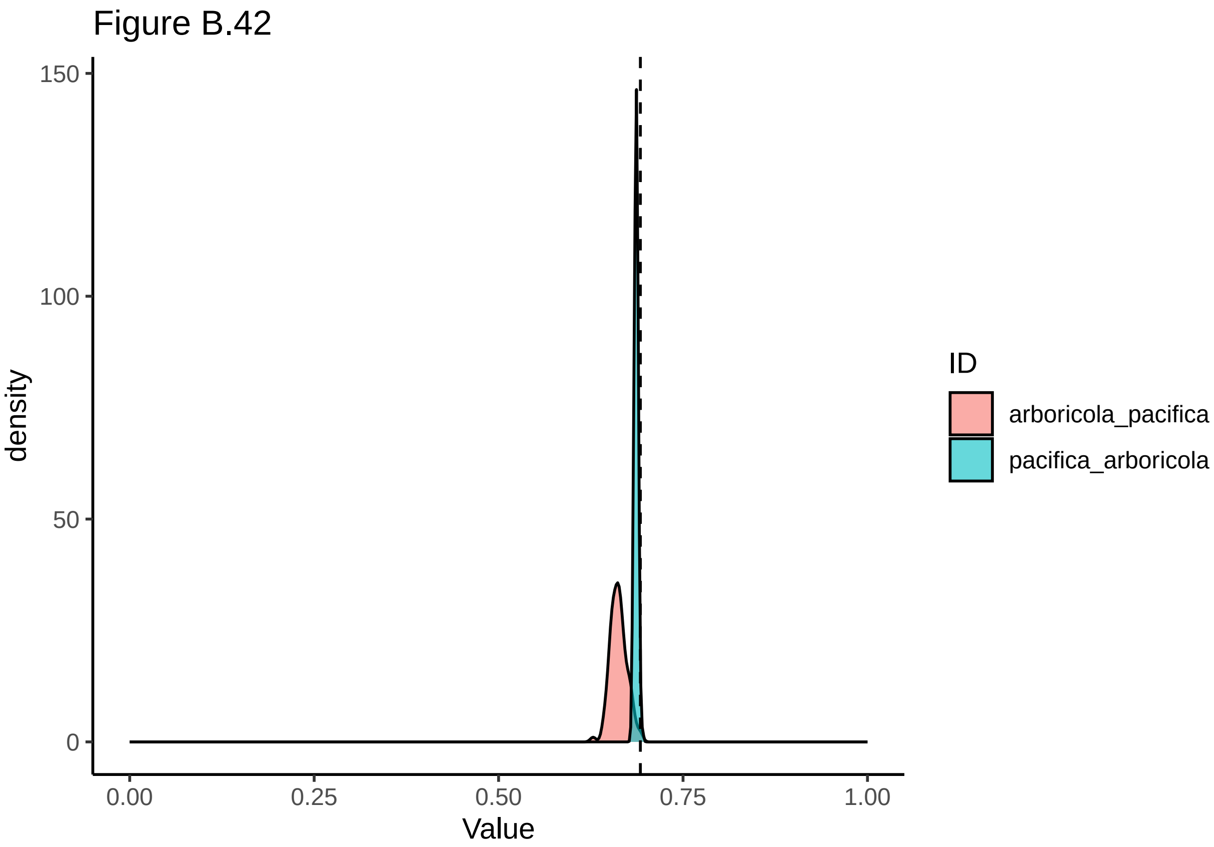


```
## [1] "pacifica_arboricola"
## [1] "P value for pacifica vs. arboricola = 0.98656810582395"
## [1] "Test statistic above high critical value."
## [1] "0.691532025784251; statistic = 0.692175835838449"
## [1] "arboricola_pacifica"
## [1] "P value for pacifica vs. arboricola = 0.995921993929447"
## [1] "Test statistic above high critical value."
## [1] "0.684491016861398; statistic = 0.692175835838449"
```

Figure B.43

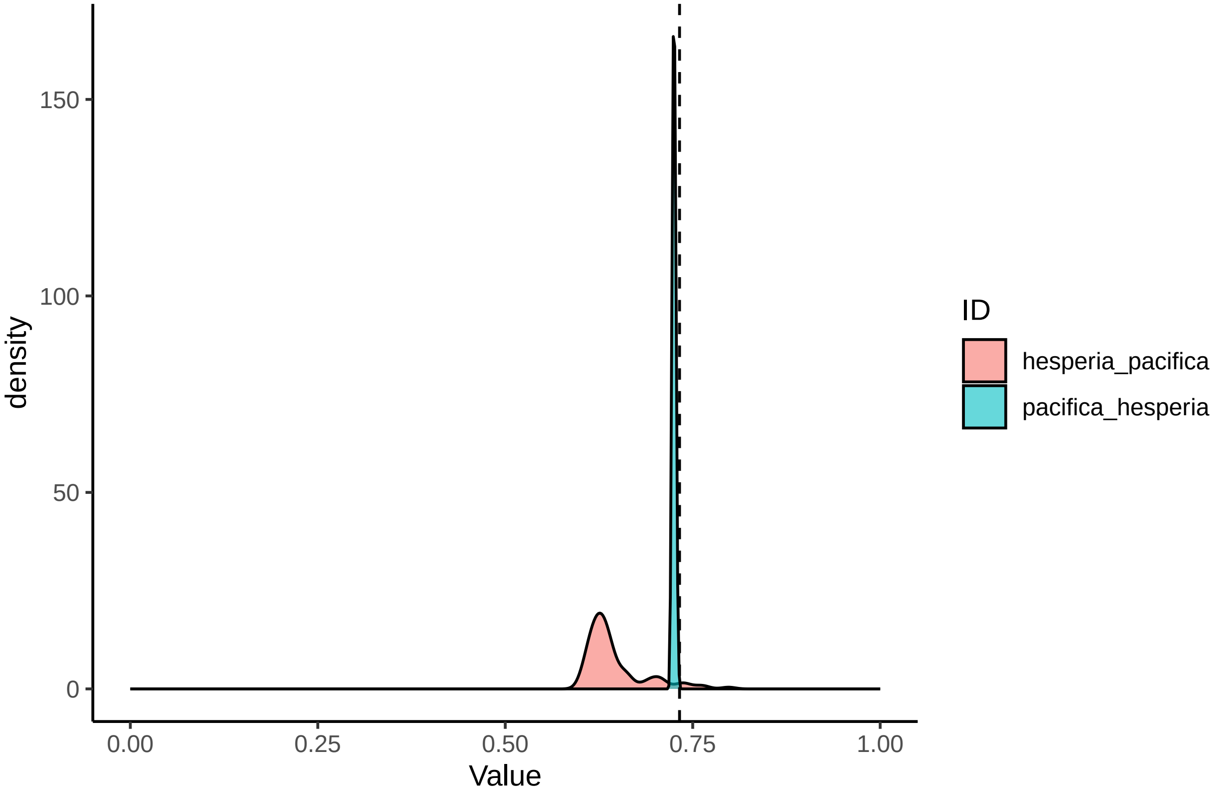


```
## [1] "pacifica_hesperia"
## [1] "P value for pacifica vs. hesperia = 0.999810707015926"
## [1] "Test statistic above high critical value."
## [1] "0.729138438790516; statistic = 0.732404918264699"
## [1] "hesperia_pacifica"
## [1] "P value for pacifica vs. hesperia = 0.985861219536583"
## [1] "Test statistic above high critical value."
## [1] "0.723152555455607; statistic = 0.732404918264699"
```

Figure B.45

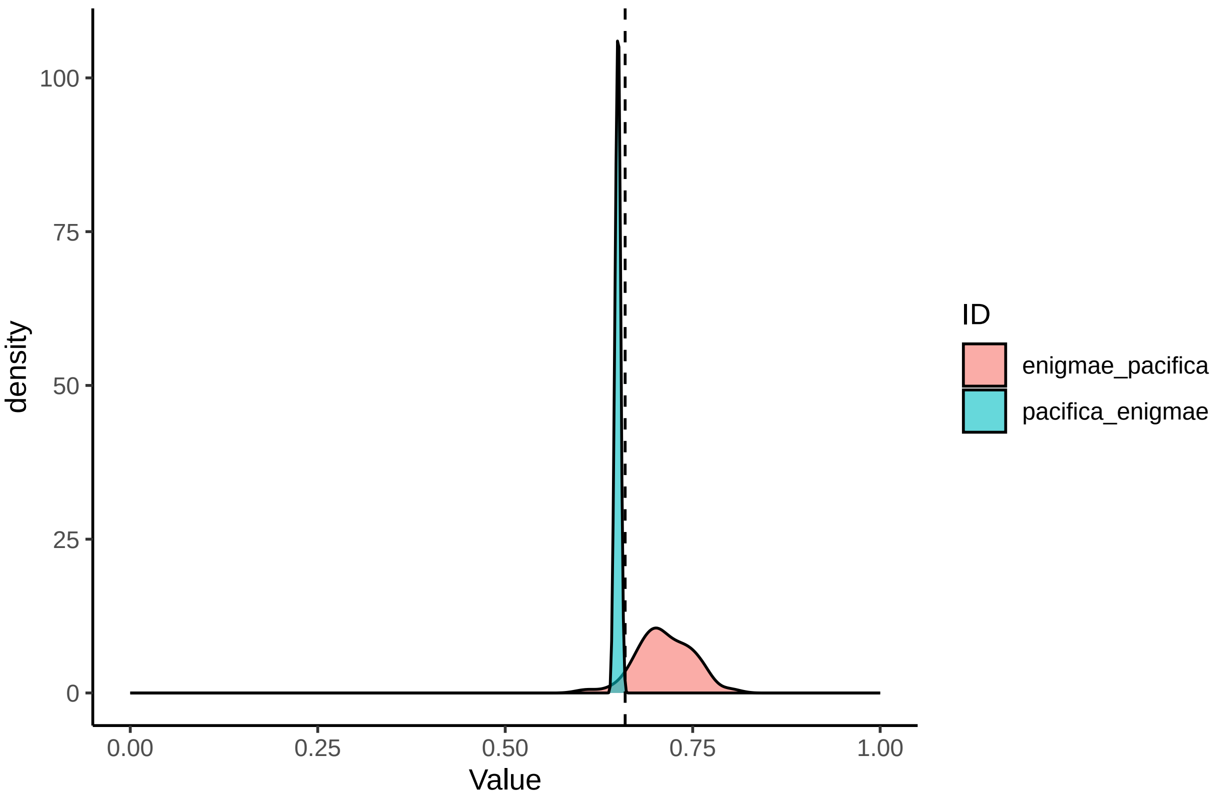


```
## [1] "pacifica_enigmae"
## [1] "P value for pacifica vs. enigmae = 0.999118403218665"
## [1] "Test statistic above high critical value."
## [1] "0.656257251372496; statistic = 0.659932933213162"
## [1] "enigmae_pacifica"
## [1] "P value for pacifica vs. enigmae = 0.0750848771079954"
```

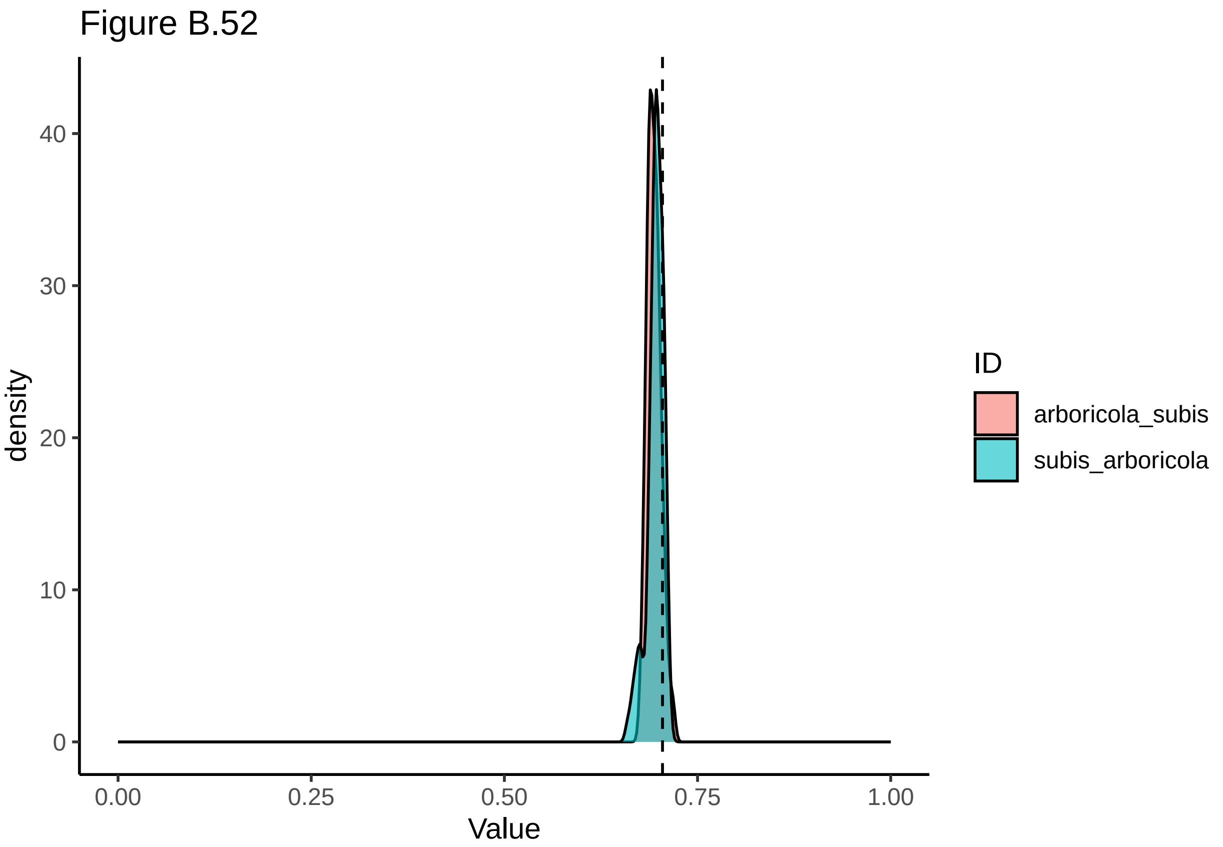


```
## [1] "subis_arboricola"
## [1] "P value for subis vs. arboricola = 0.801150285765002"
## [1] "arboricola_subis"
## [1] "P value for subis vs. arboricola = 0.897214018750521"
```

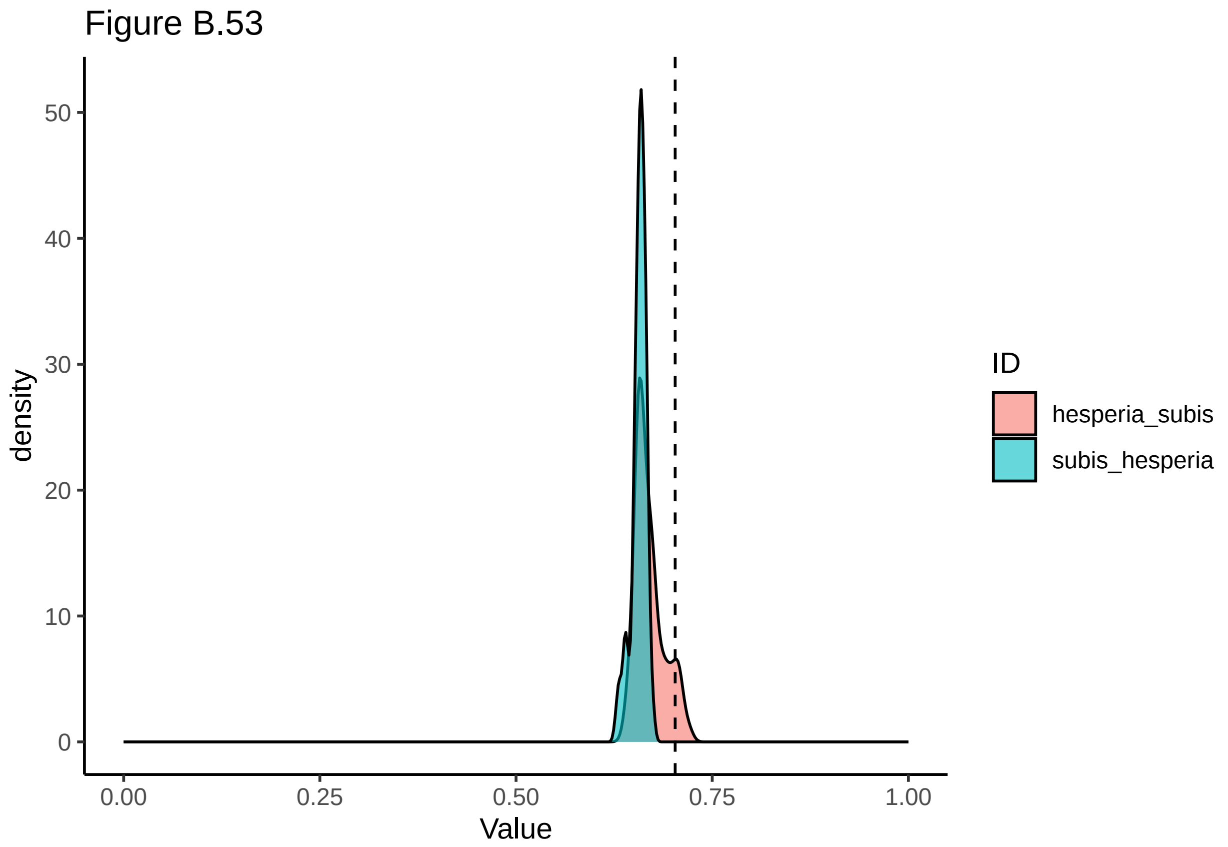


```
## [1] "subis_hesperia"
## [1] "P value for subis vs. hesperia = 0.999999562151269"
## [1] "Test statistic above high critical value."
## [1] "0.675132144914459; statistic = 0.702759858381477"
## [1] "hesperia_subis"
## [1] "P value for subis vs. hesperia = 0.963250045671483"
```

Figure B.54

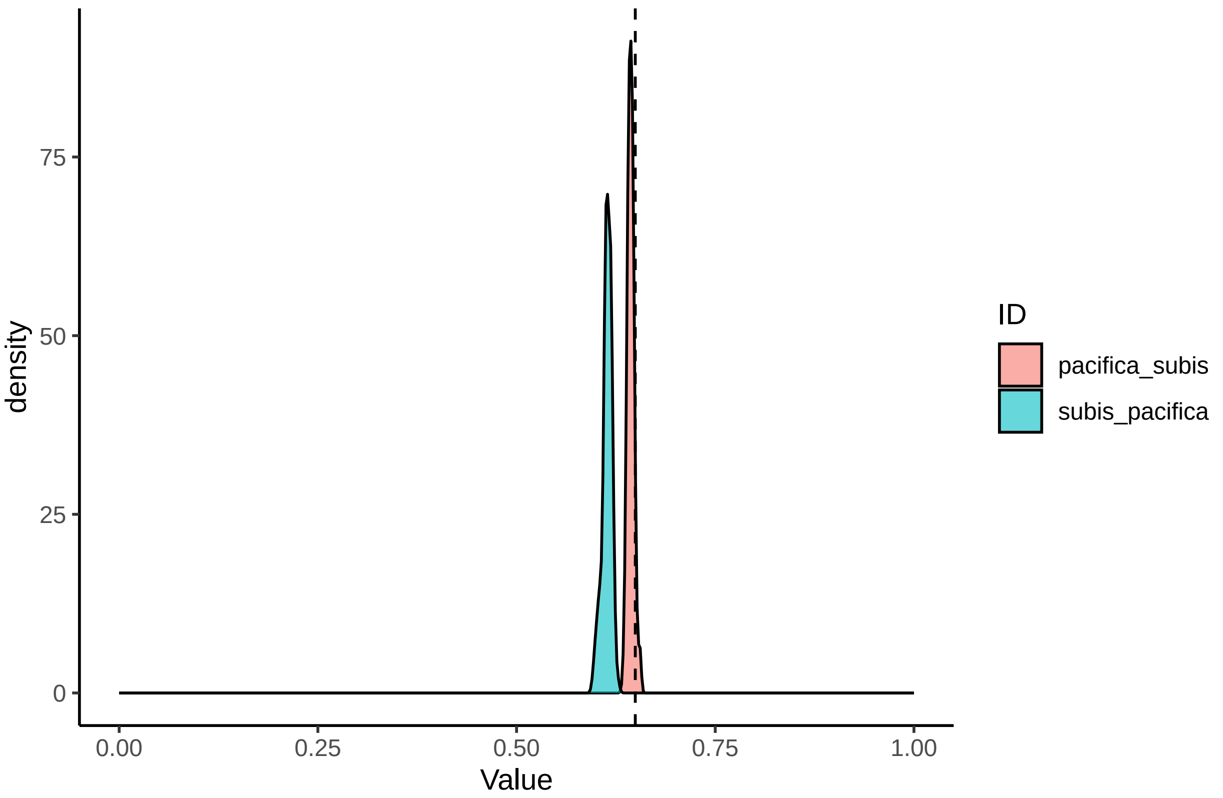


```
## [1] "subis_pacifica"
## [1] "P value for subis vs. pacifica = 0.99999999961522"
## [1] "Test statistic above high critical value."
## [1] "0.625228933535226; statistic = 0.649373031253288"
## [1] "pacifica_subis"
## [1] "P value for subis vs. pacifica = 0.923700723743345"
```

Figure B.55

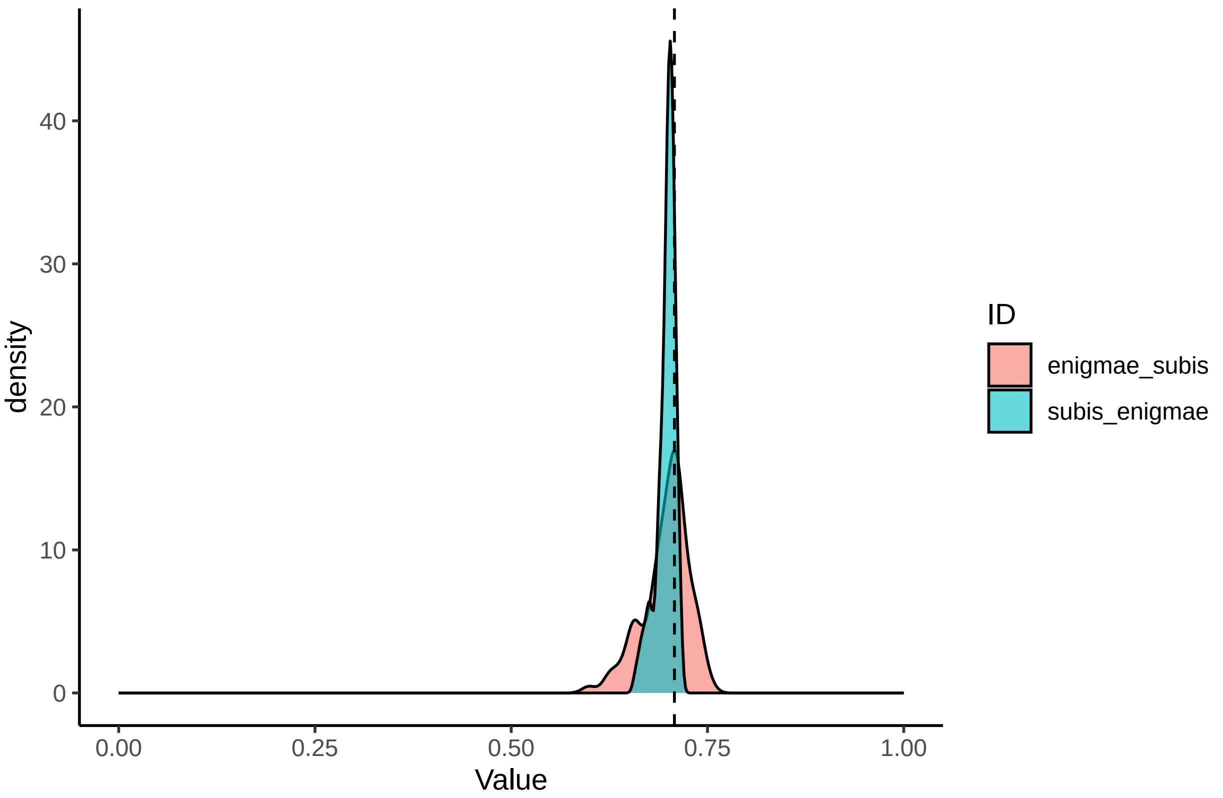


```
## [1] "subis_enigmae"
## [1] "P value for subis vs. enigmae = 0.805847702903804"
## [1] "enigmae_subis"
## [1] "P value for subis vs. enigmae = 0.643415185715688"
```

Running the NicheEvol program in progne.

```
filepath="~/Dropbox/Manuscripts/Progne/"
```

C: NichEvol Data Preparation

```
library(geiger)
library(nichevol)
library(raster)
library(tidyverse)
library(data.table)
library(nodiv)

# load rformat_type
library(ellipsenm)
```

```
# test with one
precip=raster(paste0(GISpath,"current_2-5arcmin_annualPET.tif"))

m_files=list.files(paste0(filepath,"Ms/"),pattern="*accessible.gpkg")

occ_list=read_csv(paste0(filepath,"progne_env_extract.csv"))
```

```r
p.tree=read.tree(paste0(filepath,"Sequences/all-v2-samples-95-best.raxml.bestTree"))
```

```r
# fix tree
fnames=p.tree$tip.label

# reduce to Progne
fnames_index=which(!fnames%like%"Progne")

p.tree2=drop.tip(p.tree,fnames_index)
```

```r
# plot of all Progne
plot.phylo(p.tree2,cex=0.65,main="Figure C.1: All Progne")
```

Figure C.1: All Progne

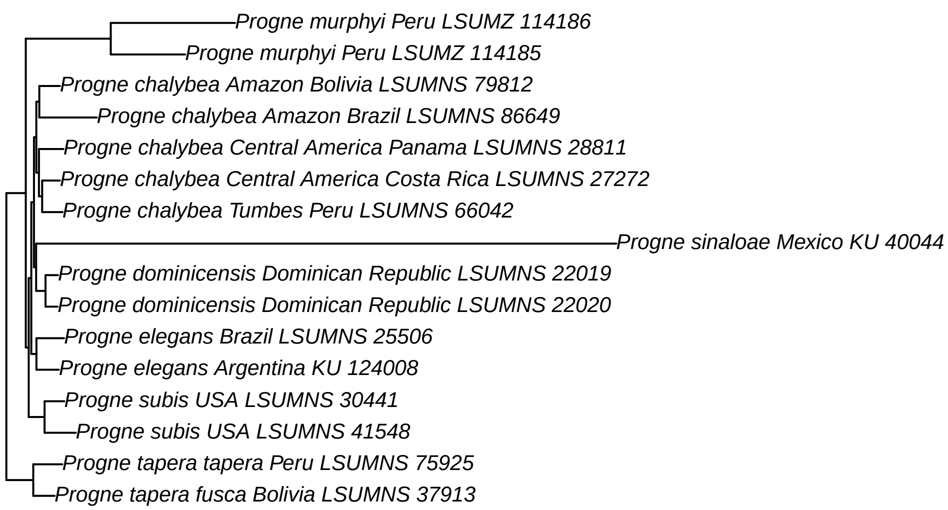

This tree is from Clare Brown's dissertation, as made available by Subir Shakya (with thanks also to Fred Sheldon). From Clare Brown's dissertation, we know that *chalybea* is monophyletic, and that it is not sister to *elegans*. We want to reduce this to a single individual per species for our next analyses.

```r
keepers=c("Progne_tapera_tapera_Peru_LSUMNS_75925",
          "Progne_chalybea_Central_America_Panama_LSUMNS_28811",
          "Progne_sinaloae_Mexico_KU_40044",
          "Progne_dominicensis_Dominican_Republic_LSUMNS_22019",
          # "AY825945.1-Progne_cryptoleuca", # not included in phylo
          "Progne_elegans_Argentina_KU_124008",
          "Progne_subis_USA_LSUMNS_41548",
          "Progne_murphyi_Peru_LSUMZ_114185")#,
          # "MH307578.1-Progne_modesta") # not included in phylo

p.tree3=drop.tip(p.tree2,c(which(p.tree2$tip.label%in%keepers==F)))
plot(p.tree3,main='Figure C.3')
```

Figure C.3

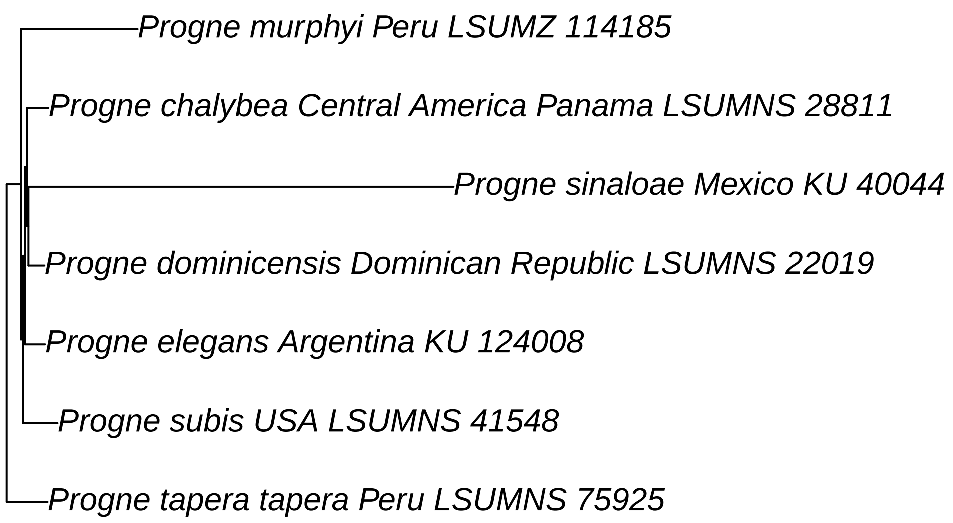


```r
# rename tips to SP only
for(i in 1:length(p.tree3$tip.label)){
  sp=strsplit(p.tree3$tip.label[i],"_")[[1]][2]
  #sp=gsub("_"," ",sp)
  p.tree3$tip.label[i]=sp
}

# reformatted other files to be the same name elsewhere
```

```r
# create ultrametric trees
phy_tree=chronos(p.tree3)
```

```
##
## Setting initial dates...
## Fitting in progress... get a first set of estimates
##          (Penalised) log-lik = -0.3001496
## Optimising rates... dates... -0.3001496
##
## log-Lik = -0.2990202
## PHIIC = 34.6
```

```r
# make edges nonzero
# step not necessary
#p.tree4$edge.length=p.tree4$edge.length+
#  0.0000000000000000000000001
```

While not included in this dataset, we can also place some species from Moyle et al. (2008), and use these estimates to help in the reconstruction.

1. *P. cryptoleuca* is sister to *P. dominicensis*.
2. There is no molecular evidence for the placement of *P. modesta*.

```r
write.tree(phy_tree,file=paste0(filepath,"new_tree.tre"))
```

Added taxon in file.

```r
phy_tree=read.tree(file=paste0(filepath,"new_tree2.tre"))
plot(phy_tree)
```

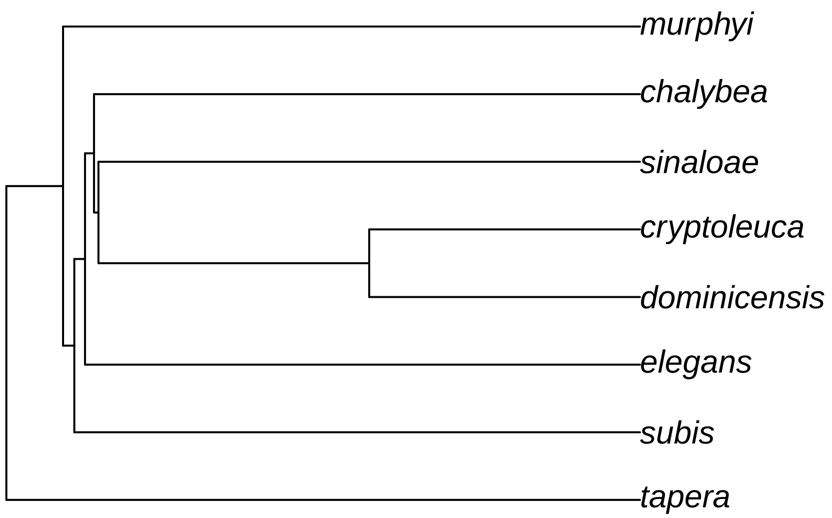

D: Occurrences and Environmental Data

We need to split occurrences into separate files, and put them in a separate folder.

```
# split occ_list into files named the same as the tips

spp=unique(occ_list$Species)

for(i in 1:length(spp)){
  sp=spp[i]
  occ_list%>%
    filter(Species==sp)%>%
    write_csv(file=paste0(filepath,"occ_dir/",
                          gsub(" ","_",sp),".csv"))
}
```

There is an error in the code. I removed it and then it was able to run. Specifically, in line 86, I removed reference to the "species" corresponding to that iteration. Note also that the files in each have to be an exact match.

```
source(paste0(filepath,'stats_evalues2.r'))
```

```
stats_evalues2(stats=c("mean","median","sd","range"),
              M_folder=paste0(filepath,"Ms/name_species_tree/"),M_format="gpkg",
              occ_folder=paste0(filepath,"occ_dir/"),
              longitude = "Long",latitude = "Lat",
              var_folder=GIS,var_format="GTiff", # must specify GTiff
              save=T,output_directory = paste0(filepath,"nichevol/"),
              overwrite = T)
```

Testing the next section with annual PET.

```
# load both files for annualPET
progne.stats=
  read_csv(paste0(filepath,
                "nichevol/current_2.5arcmin_annualPET_M_stats.csv"))%>%
  filter(Species!="Progne modesta.gpkg")
m.stats=
```

```r
  read_csv(paste0(filepath,
                  "nichevol/current_2.5arcmin_annualPET_Occurrence_stats.csv"))%>%
  filter(Species!="Progne modesta.gpkg")
```

These values may vary, as the document renders them each time. Set seed should fix this, see beginning of document; `set.seed(2024)`.

```r
bins=bintables_jc(M_folder=paste0(filepath,"Ms/name_species_tree/"),
                  M_format="gpkg",occ_folder=paste0(filepath,"occ_dir/"),
                  longitude = "Long",latitude = "Lat",
                  percentage_out = 0,bin_size = 10,
                  var_folder=GIS,var_format="GTiff") # must specify GTiff
```

Link the tree to the data, and then iteratively perform the reconstructions.

```r
### loop through and create tree projects

phy_tree2=phy_tree
phy_tree2$tip.label=c("Progne tapera","Progne subis",
                      "Progne elegans","Progne dominicensis",
                      "Progne cryptoleuca","Progne sinaloae",
                      "Progne chalybea","Progne murphyi")

for(i in 1:length(bins)){
  # link tree and data from list object
  var=strsplit(names(bins[i]),"_")[[1]][3]
  if(var=="annualPET"){var="Annual Potential Evapotranspiration"}
  if(var=='aridityIndexThornthwaite'){var='Aridity Index'}
  if(var=='climaticMoistureIndex'){var='Climatic Moisture Index'}
  if(var=='continentality'){var='Continentality'}
  if(var=='embergerQ'){var="Emberger's Pluviometric Quotient"}
  if(var=='maxTempColdest'){var='Max. Temp. of the Coldest Quarter'}
  if(var=='minTempWarmest'){var='Min. Temp. of the Warmest Quarter'}
  if(var=='PETColdestQuarter'){var='Coldest Quarter Potential Evapotranspiration'}
  if(var=='PETDriestQuarter'){var='Driest Quarter Potential Evapotranspiration'}
  if(var=='PETseasonality'){var='Seasonality of Potential Evapotranspiration'}
  if(var=='PETWarmestQuarter'){var='Warmest Quarter Potential Evapotranspiration'}
  if(var=='PETWettestQuarter'){var='Wettest Quarter Potential Evapotranspiration'}
  if(var=='thermicityIndex'){var='Thermicity Index'}
  varproj=treedata(phy_tree2,bins[[i]]) # convert chronos to phylo???
  # create tables for reconstructions
  ml_rec_table=bin_ml_rec(varproj)
  s_ml_rec_table=smooth_rec(ml_rec_table)

  base=basal_node(phy_tree2)

  var.lab.1=paste0('Figure D.',i,'A: ',var)
  var.lab.2=paste0('Figure D.',i,'B: ',var)

  plot.phylo(phy_tree2,label.offset = 0.06,main=var.lab.1,x.lim=4,y.lim=9)
  nichevol_labels(phy_tree2, s_ml_rec_table, height = 1.25, width = 3)
  niche_labels(phy_tree2,s_ml_rec_table,
               label_type = 'tip_node',height=1.25, width = 6)
  nichevol_legend(position = "bottomright", cex = 1)
```

```
  print("Starting LevelPlot...")

  # adapted from Fernando Machado-Stredel
  bin_tabl_tr=t(s_ml_rec_table)
  bin_tabl_tr[bin_tabl_tr == "?"] <- NA
  nodes=c(which(colnames(bin_tabl_tr)%like%"Progne"),base)
  colnames(bin_tabl_tr)=gsub(base,"Basal Node",colnames(bin_tabl_tr))

  # get column order to understand
  # Phylogenetic placement - out to in, large to small
  col_order=rev(c("Basal Node",
                  "Progne tapera",
                  "Progne murphyi",
                  "Progne subis",
                  "Progne elegans",
                  "Progne chalybea",
                  "Progne sinaloae",
                  "Progne dominicensis",
                  "Progne cryptoleuca"))

  col_theme <- rasterTheme(region = c("#377eb8","#e41a1c"))
  plot2=levelplot(bin_tabl_tr[,col_order], scales = list(x = list(rot = 90)), # labels
          xlab = var.lab.2, ylab = "",
          par.settings = col_theme, colorkey = FALSE) # color options

  print(plot2)
  print("Complete.")
}
```

Figure D.1A: Annual Potential Evapotranspiration

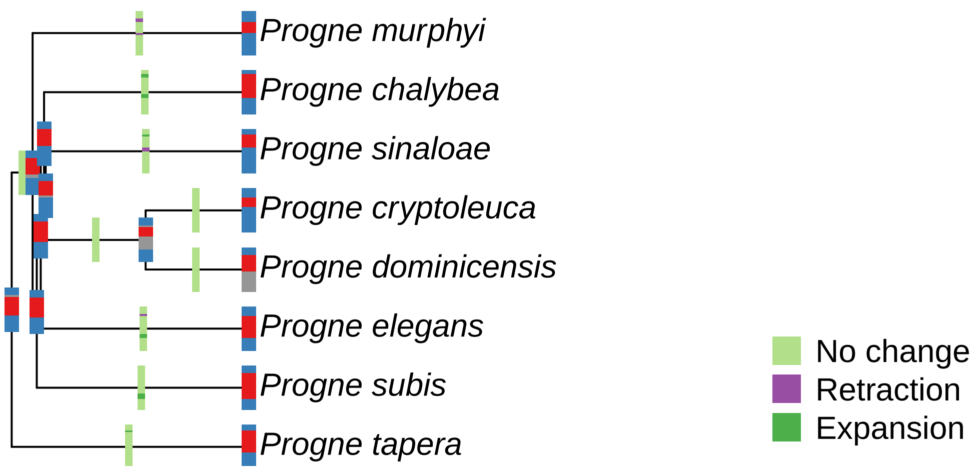


```
## [1] "Starting LevelPlot..."
```

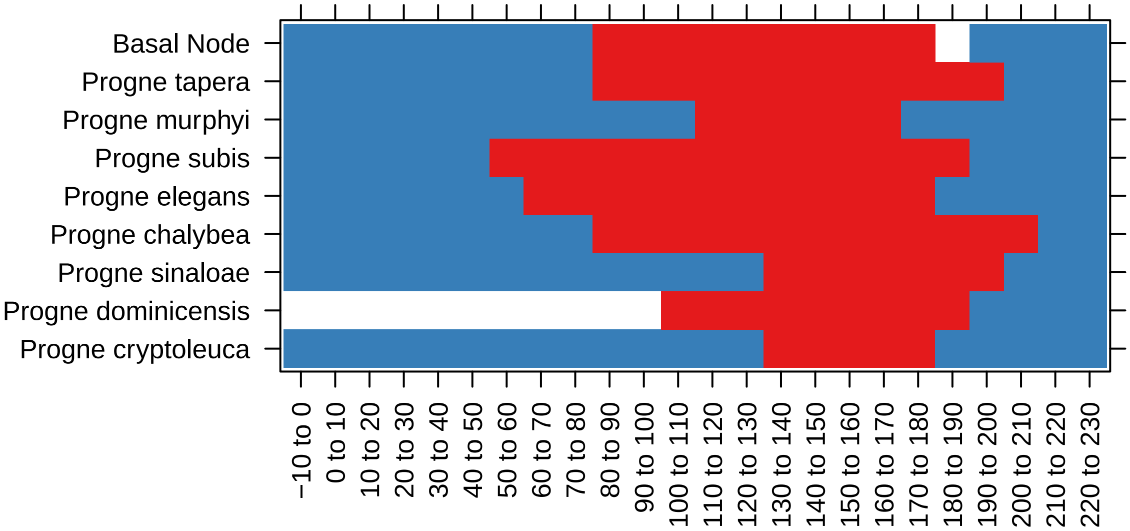

Figure D.1B: Annual Potential Evapotranspiration

```
## [1] "Complete."
```

Figure D.2A: Aridity Index

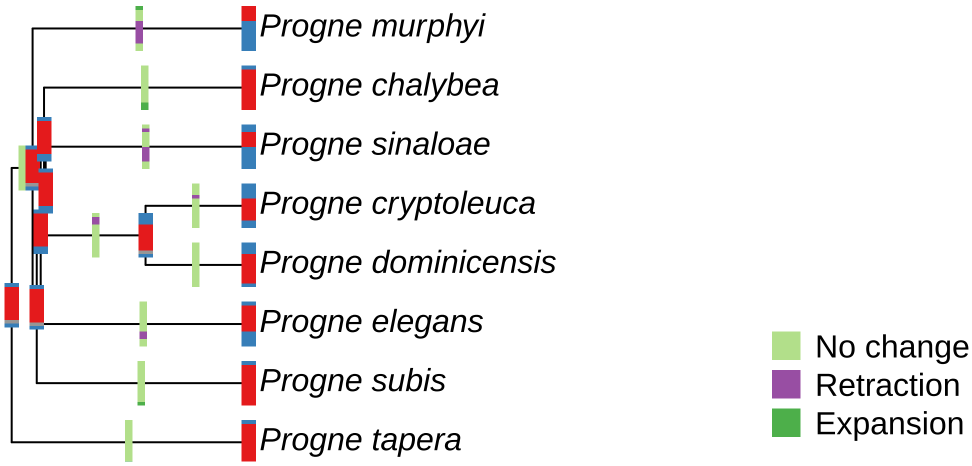


```
## [1] "Starting LevelPlot..."
```

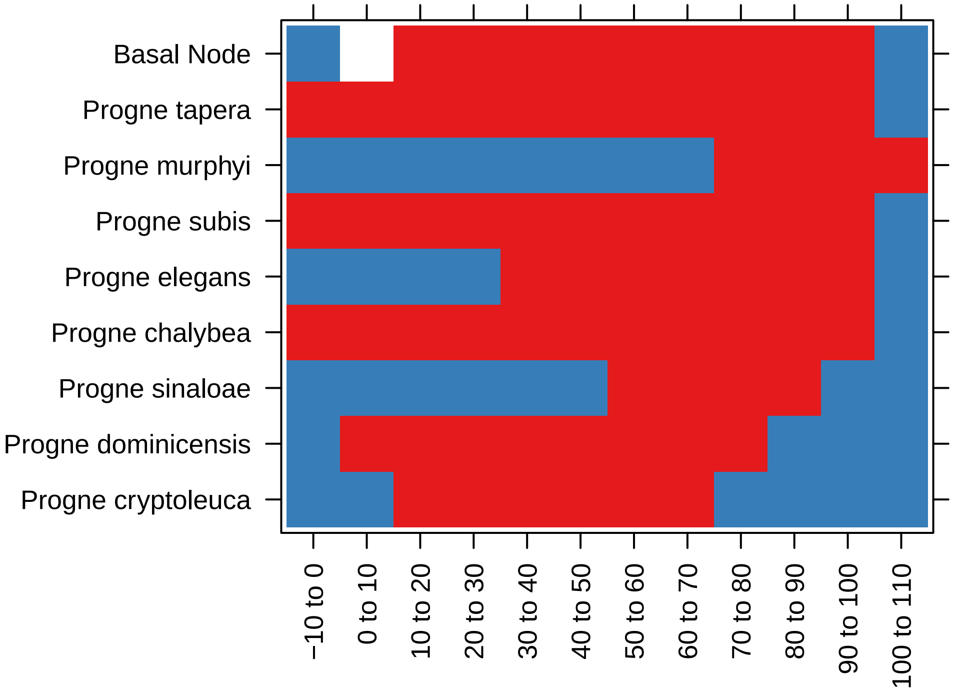

Figure D.2B: Aridity Index

```
## [1] "Complete."
```

Figure D.3A: Climatic Moisture Index

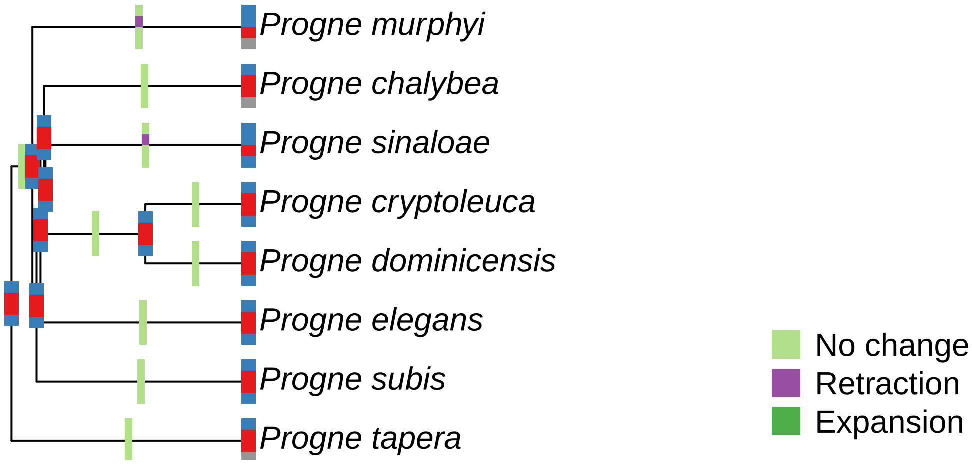


```
## [1] "Starting LevelPlot..."
```

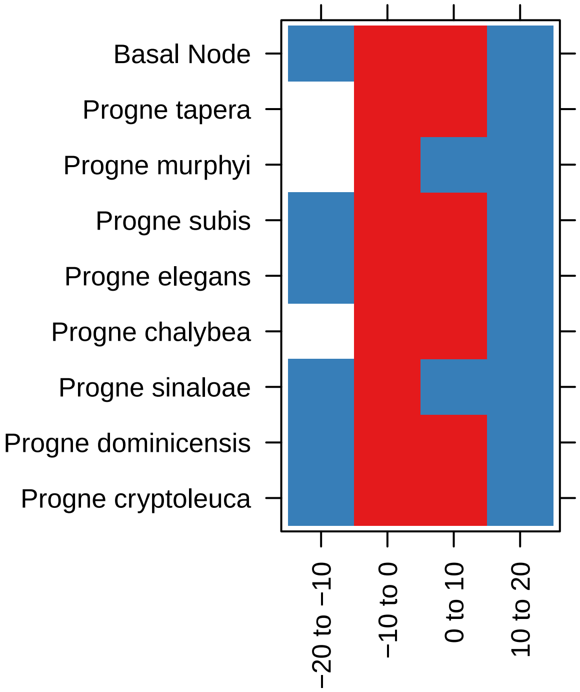

Figure D.3B: Climatic Moisture Index

```
## [1] "Complete."
```

Figure D.4A: Continentality

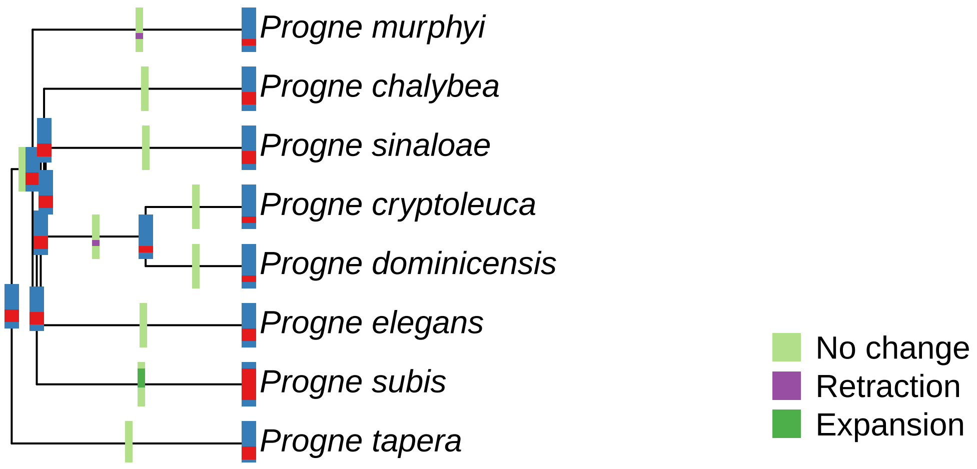


```
## [1] "Starting LevelPlot..."
```

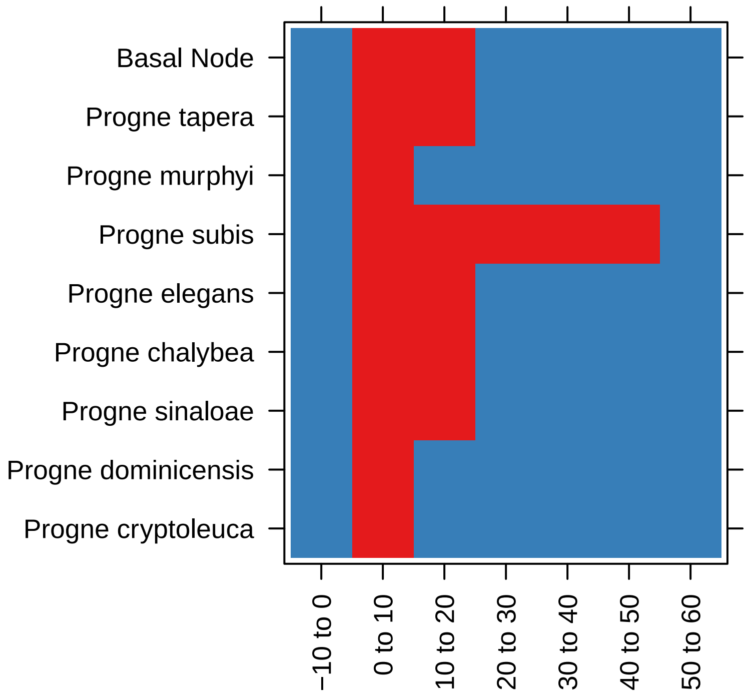

Figure D.4B: Continentality

```
## [1] "Complete."
```

Figure D.5A: Emberger's Pluviometric Quotient

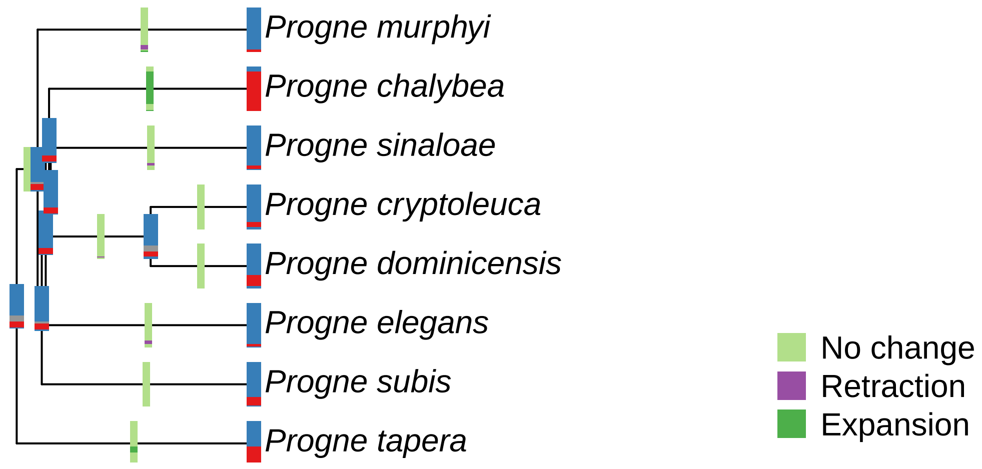


```
## [1] "Starting LevelPlot..."
```

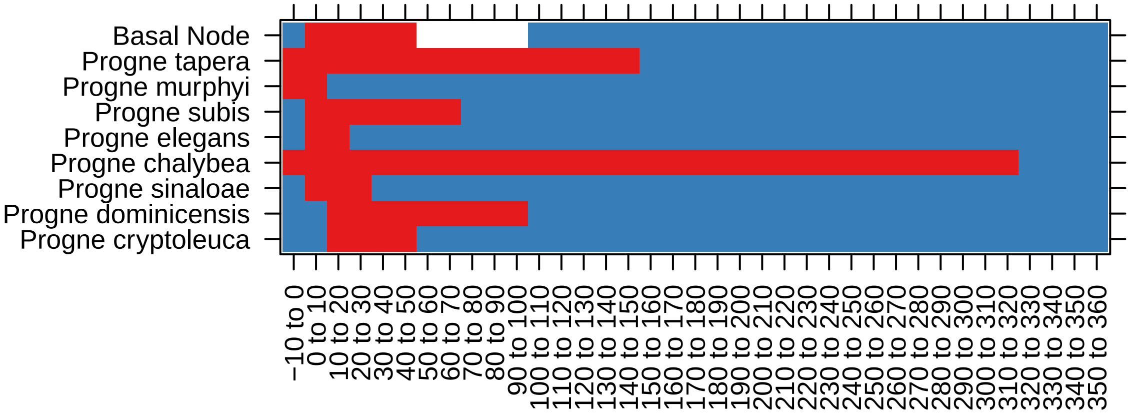

Figure D.5B: Emberger's Pluviometric Quotient

```
## [1] "Complete."
```

Figure D.6A: growingDegDays0

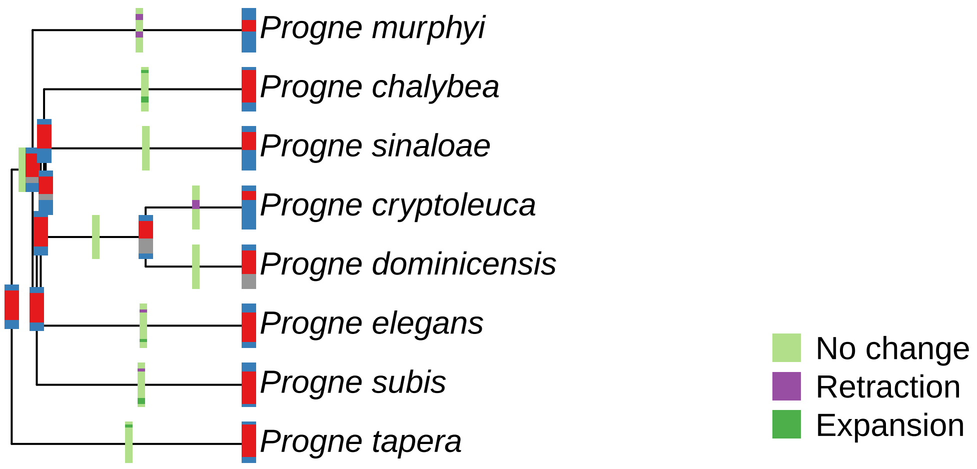


```
## [1] "Starting LevelPlot..."
```

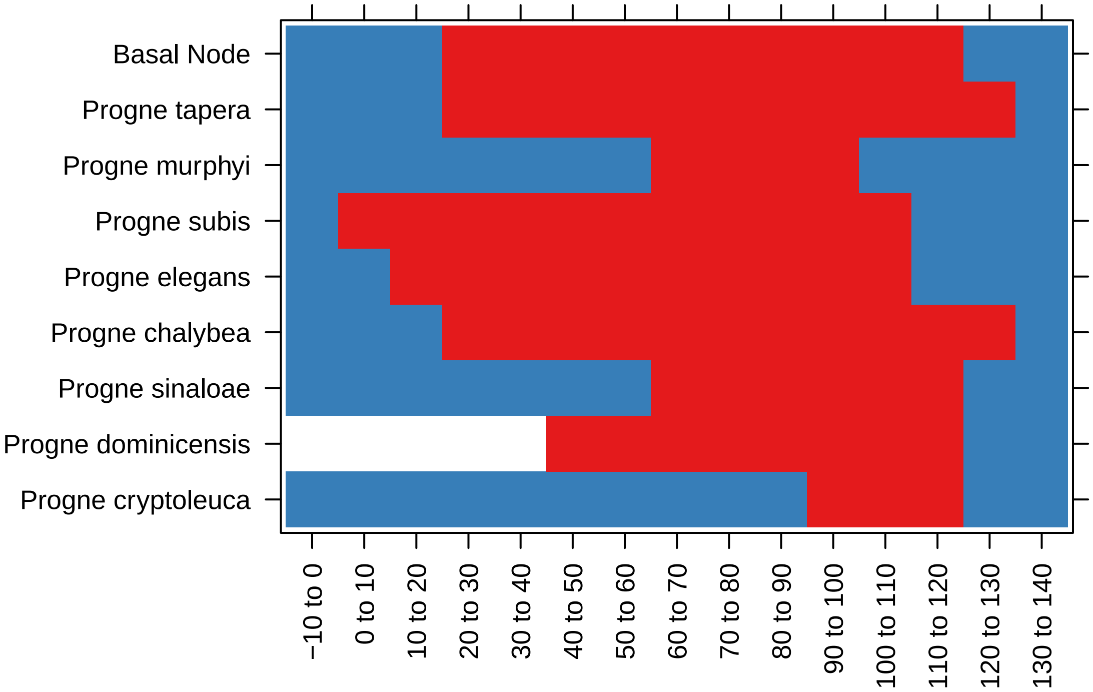

Figure D.6B: growingDegDays0

```
## [1] "Complete."
```

Figure D.7A: growingDegDays5

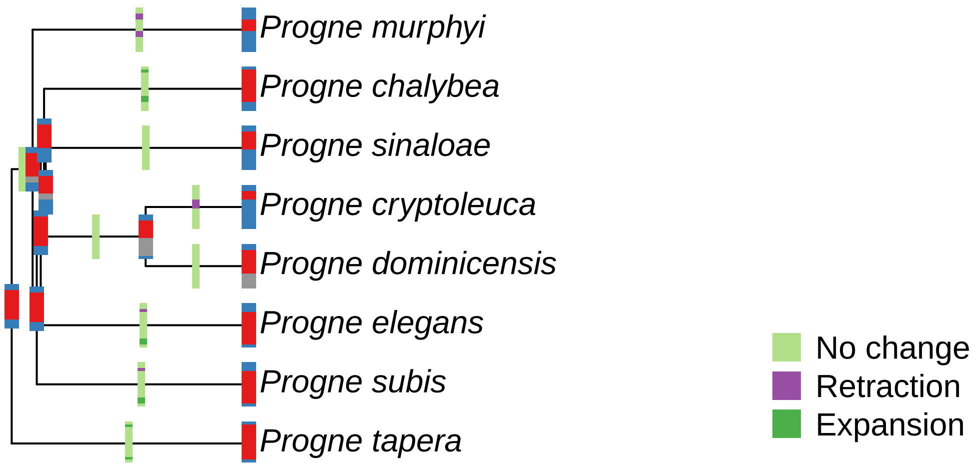


```
## [1] "Starting LevelPlot..."
```

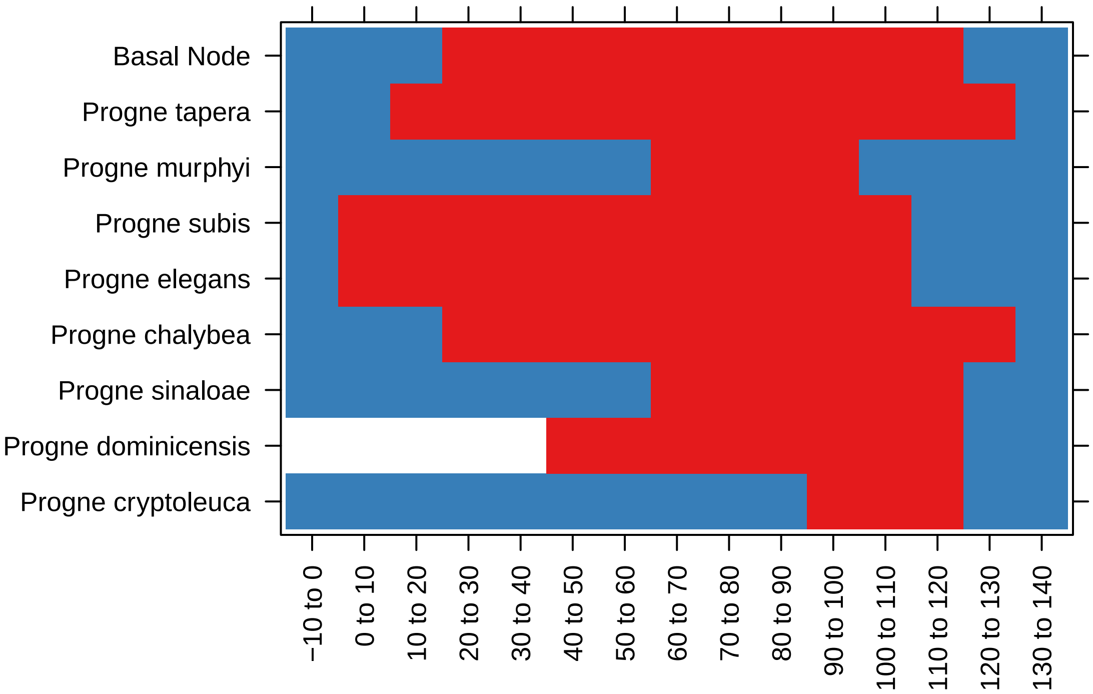

Figure D.7B: growingDegDays5

```
## [1] "Complete."
```

Figure D.8A: Max. Temp. of the Coldest Quarter

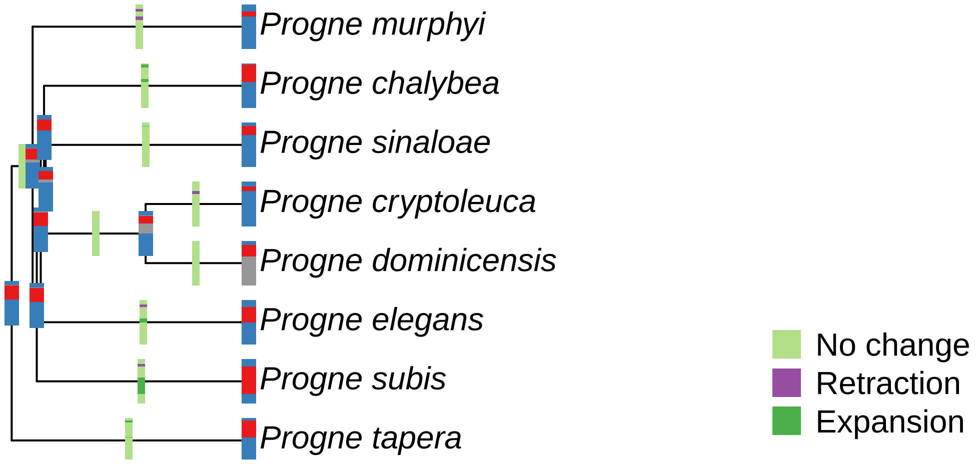


```
## [1] "Starting LevelPlot..."
```

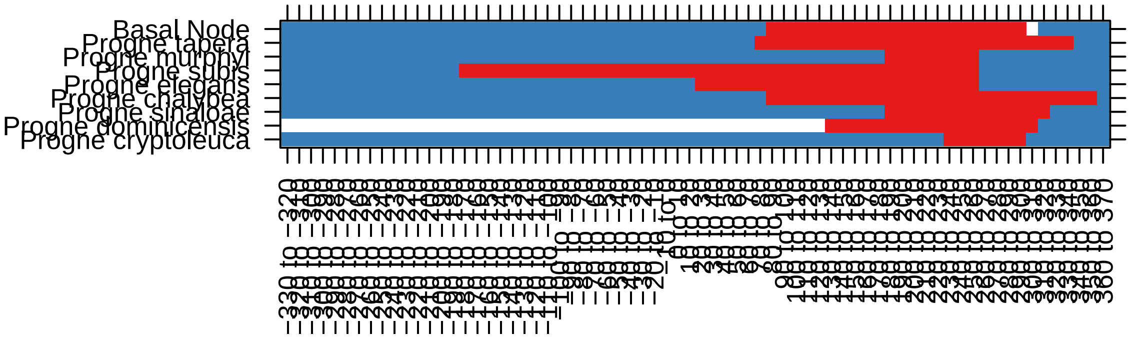

Figure D.8B: Max. Temp. of the Coldest Quarter

```
## [1] "Complete."
```

Figure D.9A: Min. Temp. of the Warmest Quarter

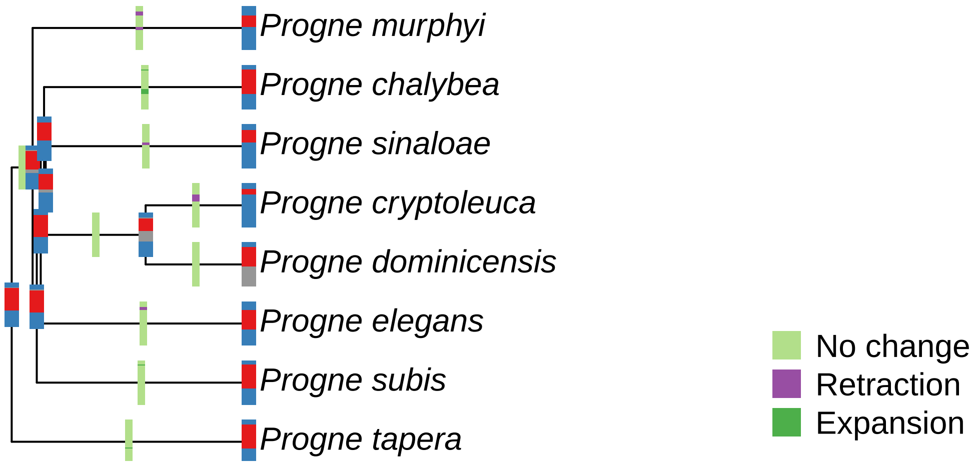


```
## [1] "Starting LevelPlot..."
```

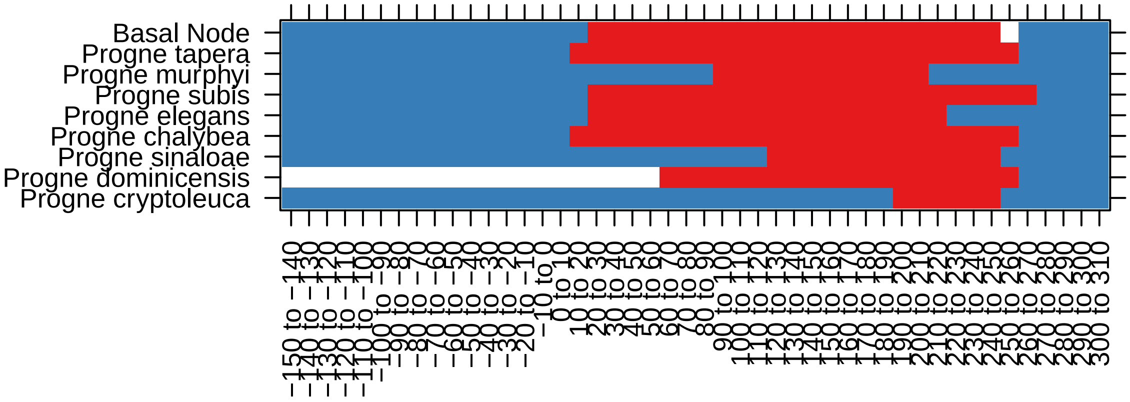

Figure D.9B: Min. Temp. of the Warmest Quarter

```
## [1] "Complete."
```

Figure D.10A: monthCountByTemp10

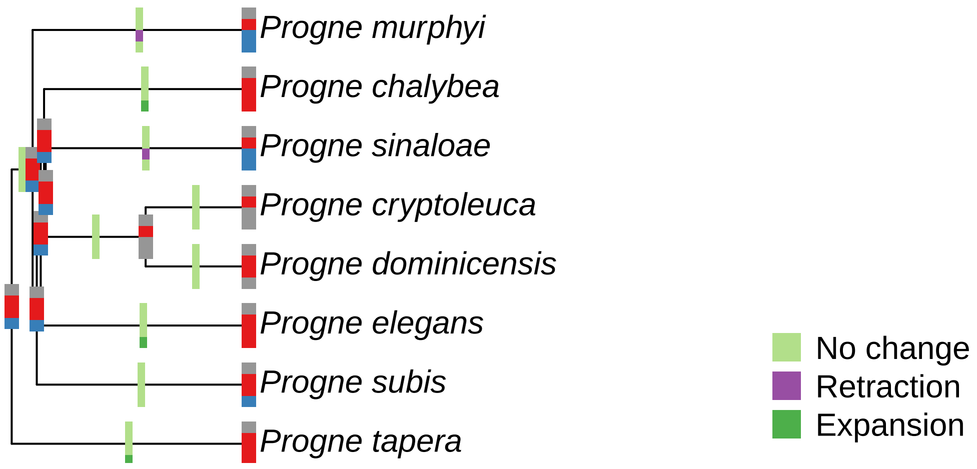


```
## [1] "Starting LevelPlot..."
```

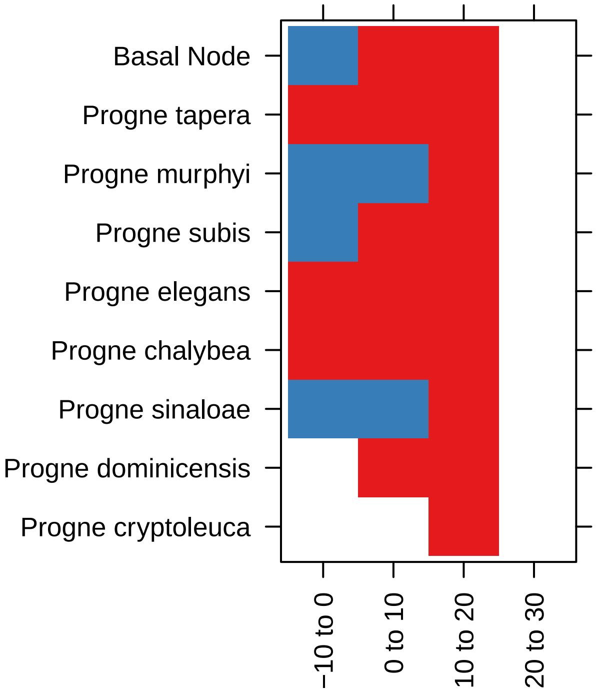

Figure D.10B: monthCountByTemp10

```
## [1] "Complete."
```

Figure D.11A: Coldest Quarter Potential Evapotranspiration

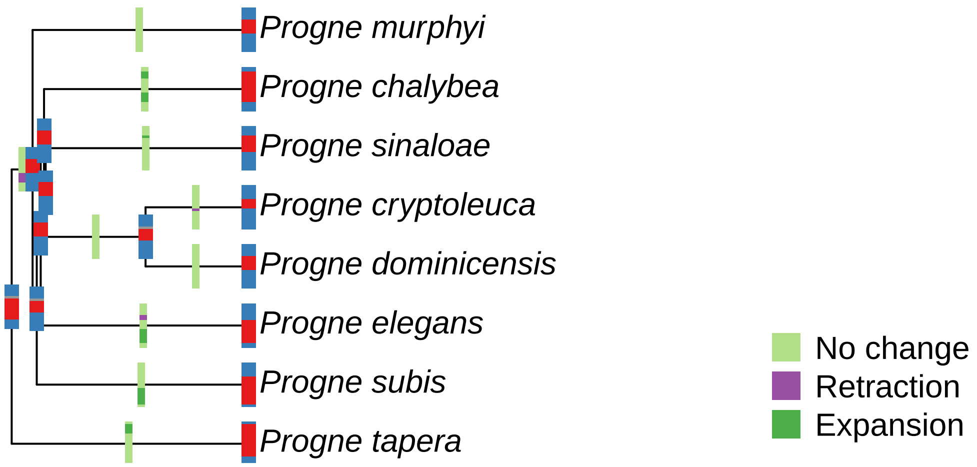


```
## [1] "Starting LevelPlot..."
```

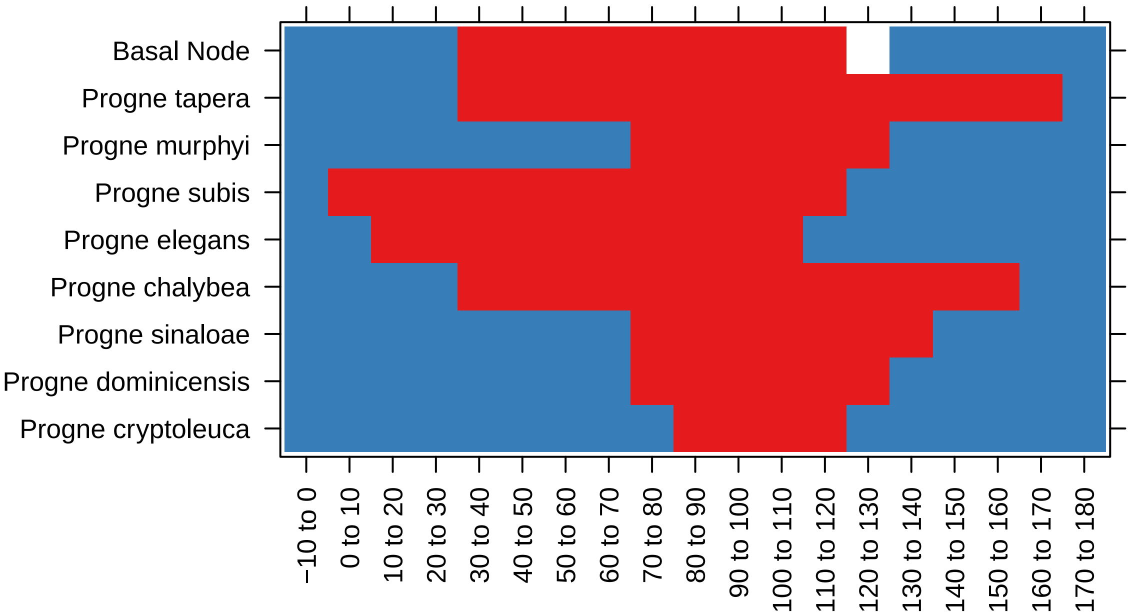

Figure D.11B: Coldest Quarter Potential Evapotranspiration

```
## [1] "Complete."
```

Figure D.12A: Driest Quarter Potential Evapotranspiration

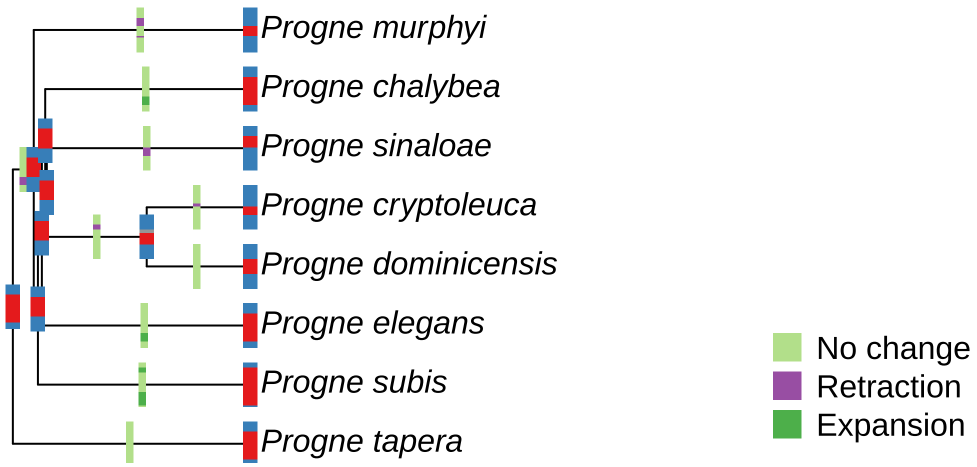


```
## [1] "Starting LevelPlot..."
```

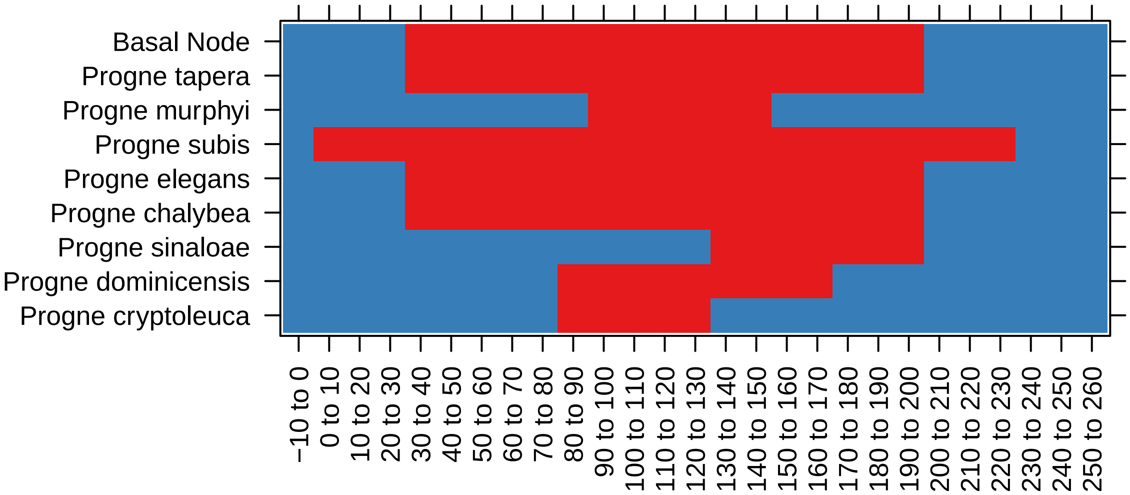

Figure D.12B: Driest Quarter Potential Evapotranspiration

```
## [1] "Complete."
```

Figure D.13A: Seasonality of Potential Evapotranspiration

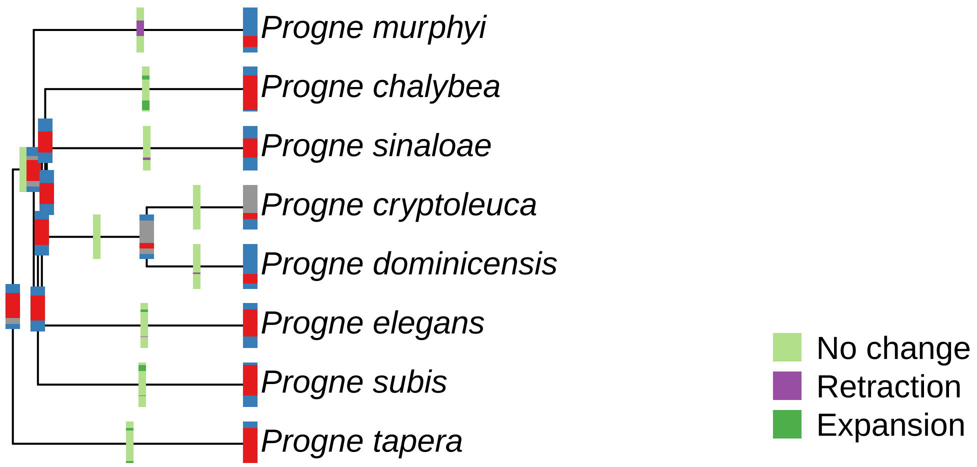


```
## [1] "Starting LevelPlot..."
```

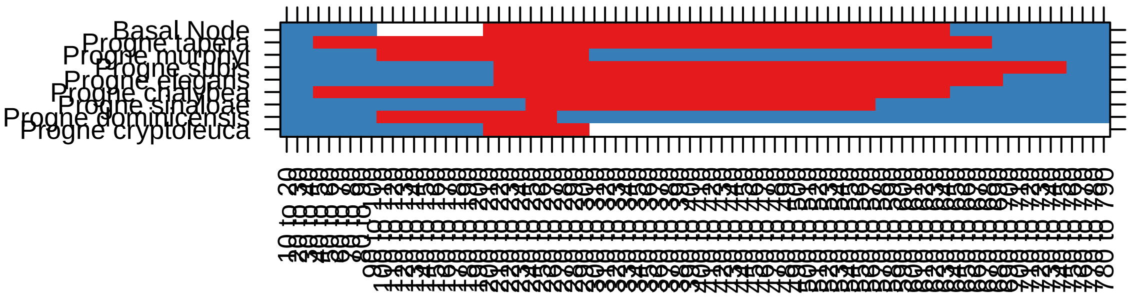

Figure D.13B: Seasonality of Potential Evapotranspiration

```
## [1] "Complete."
```

Figure D.14A: Warmest Quarter Potential Evapotranspiration

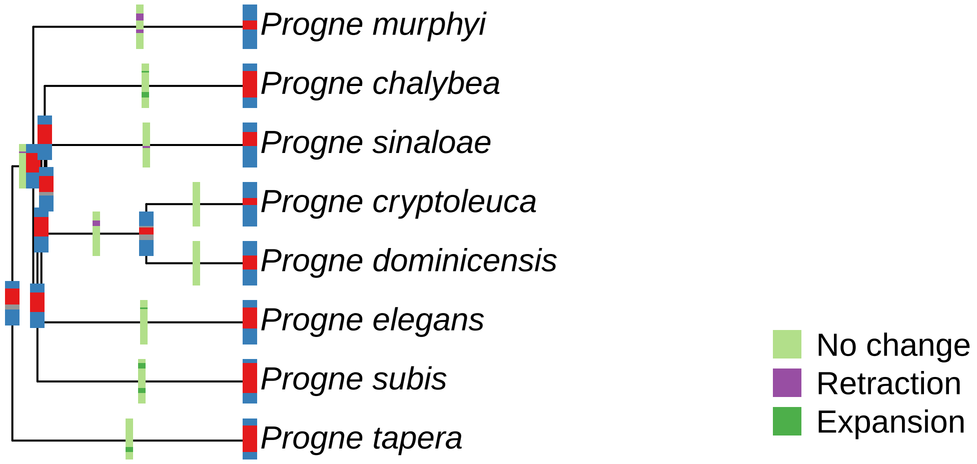

[1] "Starting LevelPlot..."

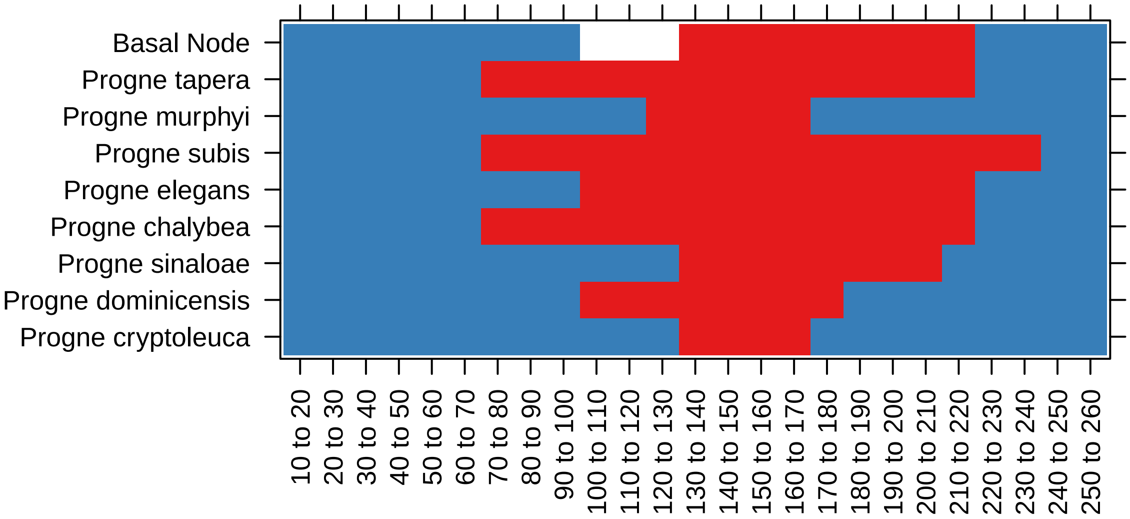

Figure D.14B: Warmest Quarter Potential Evapotranspiration

[1] "Complete."

Figure D.15A: Wettest Quarter Potential Evapotranspiration

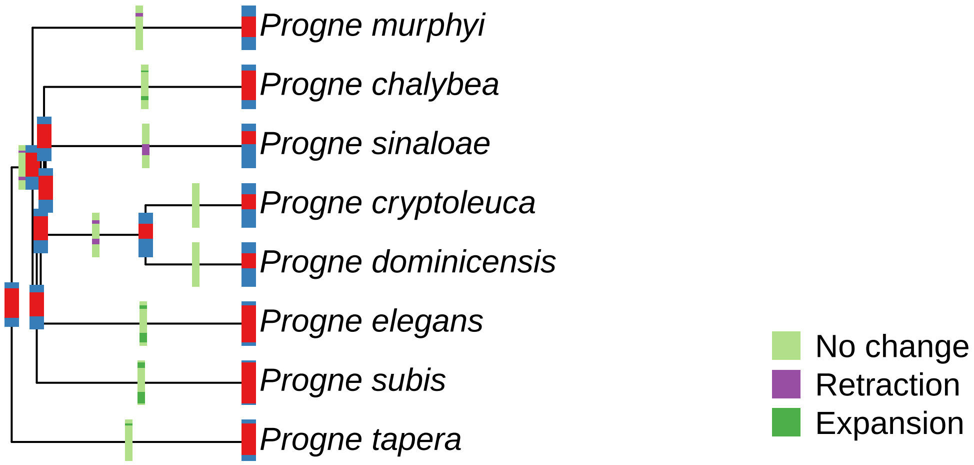

[1] "Starting LevelPlot..."

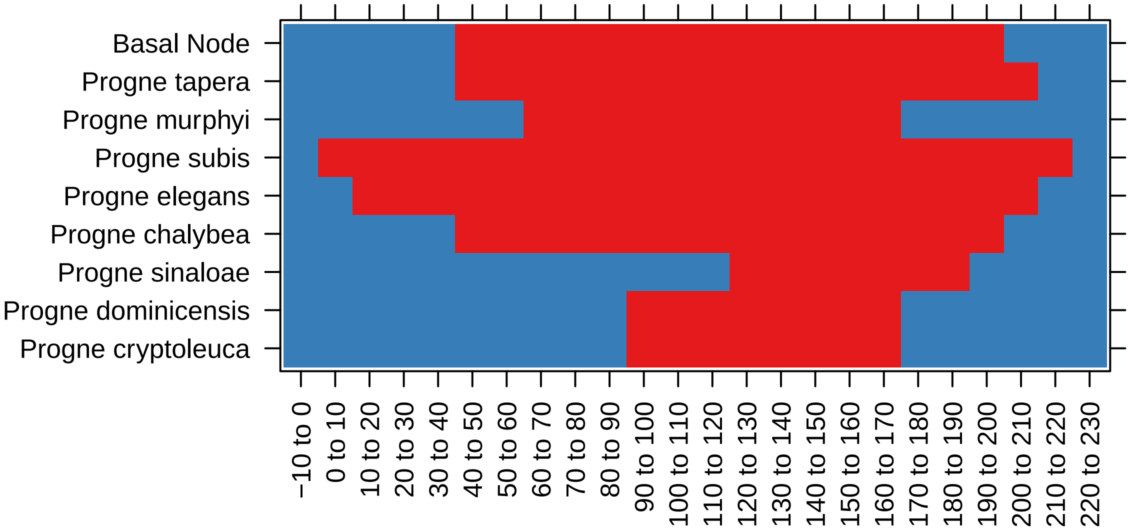

Figure D.15B: Wettest Quarter Potential Evapotranspiration

```
## [1] "Complete."
```

Figure D.16A: Thermicity Index

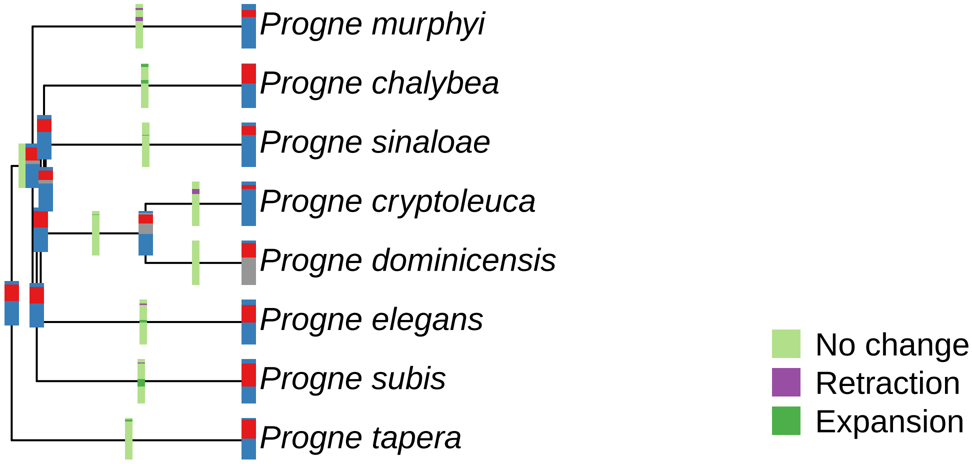


```
## [1] "Starting LevelPlot..."
```

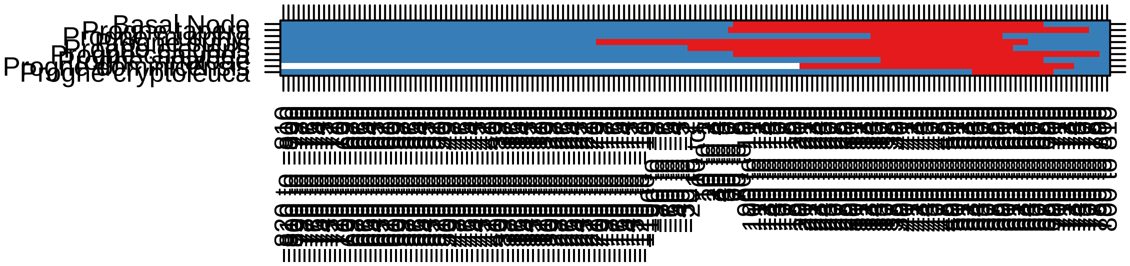

Figure D.16B: Thermicity Index

```
## [1] "Complete."
```
